# Supplementary material for: Transcriptome Sequencing of Broussonetia papyrifera Leaves Reveals Key Genes Involved in Flavonoids Biosynthesis
Source: Plants (Basel). 2023 Jan 26;12(3):563. doi: 10.3390/plants12030563 (PMC9920218; doi:10.3390/plants12030563)
Supplement: Supplementary file 1 [file plants-12-00563-s001.zip › Supplementary Files/Table S2-8.docx]

# Table S2 Primers used for qRT-PCR analysis in this study.

| Gene_ID | Forward primer | Reverse primer |
| --- | --- | --- |
| *Actin* | CCCAATCCAAGAGAGGTATCCTTAC | GTCATCTTTTCTCTGTTAGCCTTGG |
| Cluster-10108.5296 | TGAGTGGCTTGGCCTCAAAT | CGGACCAGAATGTAGACGGG |
| Cluster-10108.10116 | GTCTACAACCTCTCCCACGC | GAGGAAGCAGATCGGGTAGC |
| Cluster-10108.22335 | CGATGCAGAAGCGACAGCTA | CCGTAAGTAATCCGGTCGGT |
| Cluster-10108.7225 | TTGGACGACCTCTGTGAAGC | ACAGTGTGCACTAGCGATGT |
| Cluster-10108.14936 | GGCACAAAGTCGCCAATGTT | CTAGGCCGTGTCATGCTAGG |
| Cluster-10108.10698 | CCGAGCTAGTGAACCATCCC | ACTTGTACGTGTCTGGCTCG |
| Cluster-10108.13424 | CATGGCACCTTCCCTAGACG | CGAGGTGGGTGATCTTGGAC |
| Cluster-10108.14282 | TCTTCTGCCATGCAACTCGT | ATAGTGGGAGCCTCAGGGTT |
| Cluster-10108.7191 | CCTCCCTTCCGACCCTTTTC | CAGGGATTTCACCAGACGCT |
| Cluster-10108.4707 | ATTGTGCTGGCCCCTGATAC | GAGGCTGGAGCCAGCTTTTA |

# Table S3 The FPKM value and the 2−^ΔΔCt^ value of DEGs verified by qRT-PCR.

| ID | FPKM | | | | | | | | |  |
| --- | --- | --- | --- | --- | --- | --- | --- | --- | --- | --- |
|  | T1-1 | T1-2 | T1-3 | T3-1 | T3-2 | T3-3 | T4-1 | T4-2 | T4-3 | |
| Cluster-10108.5296 | 2.27 | 0.58 | 2.99 | 14.13 | 6.15 | 2.11 | 11.2 | 19.97 | 45.9 | |
| Cluster-10108.10116 | 15.9 | 11.21 | 13.02 | 21.01 | 16.05 | 12.42 | 23.96 | 28.51 | 38.73 | |
| Cluster-10108.22335 | 3.76 | 2.88 | 5.02 | 20.13 | 16.28 | 9.61 | 20.56 | 23.89 | 34.24 | |
| Cluster-10108.7225 | 10.45 | 7.12 | 11.04 | 24.71 | 21.04 | 17.16 | 25.75 | 32.97 | 49.05 | |
| Cluster-10108.12273 | 26.58 | 56.38 | 37.29 | 3.51 | 2.08 | 1.56 | 4.26 | 0.96 | 0.33 | |
| Cluster-10108.14936 | 72.76 | 64.81 | 48.28 | 36.29 | 20.38 | 31.92 | 17.45 | 16.84 | 11.72 | |
| Cluster-10108.10698 | 61.04 | 59.1 | 47.29 | 38.32 | 27.67 | 33.22 | 19.32 | 13.28 | 11.69 | |
| Cluster-10108.13424 | 682.5 | 569.13 | 630.23 | 475.38 | 444.4 | 525.3 | 362.67 | 227.81 | 86.78 | |
| Cluster-10108.14282 | 12 | 11.13 | 9.02 | 0.5 | 0.12 | 0.48 | 1.14 | 0.68 | 0.65 | |
| Cluster-10108.2078 | 6.93 | 6.54 | 5.61 | 25.3 | 1.17 | 1.94 | 1.46 | 0.96 | 1.53 | |
| Cluster-10108.7191 | 5.46 | 4.53 | 7.5 | 38.36 | 42.73 | 26.81 | 49.02 | 57.7 | 56.78 | |
| Cluster-10108.4707 | 3.24 | 2.02 | 3.44 | 9.24 | 8.91 | 5.18 | 14.14 | 18.06 | 14.7 | |
|  | 2^−ΔΔCt^ | | | | | | | | |  |
| Cluster-10108.5296 | 1.02 | 1.06 | 0.93 | 7.74 | 6.62 | 7.84 | 18.46 | 14.38 | 17.59 | |
| Cluster-10108.10116 | 1.02 | 0.96 | 1.02 | 2.76 | 2.36 | 2.67 | 9.28 | 7.67 | 8.54 | |
| Cluster-10108.22335 | 1.01 | 1.05 | 0.95 | 1.33 | 2.36 | 1.69 | 5.17 | 7.63 | 6.82 | |
| Cluster-10108.7225 | 0.99 | 0.99 | 1.02 | 5.46 | 5.33 | 5.06 | 22.74 | 18.66 | 20.49 | |
| Cluster-10108.12273 | 1.03 | 0.95 | 1.01 | 0.11 | 0.14 | 0.19 | 0.00 | 0.00 | 0.00 | |
| Cluster-10108.14936 | 0.99 | 1.02 | 0.99 | 0.28 | 0.27 | 0.27 | 0.16 | 0.14 | 0.14 | |
| Cluster-10108.10698 | 0.94 | 1.04 | 1.03 | 0.66 | 0.56 | 0.48 | 0.31 | 0.33 | 0.30 | |
| Cluster-10108.13424 | 0.96 | 1.03 | 1.01 | 0.44 | 0.40 | 0.39 | 0.20 | 0.15 | 0.17 | |
| Cluster-10108.14282 | 1.07 | 1.07 | 0.88 | 0.01 | 0.01 | 0.01 | 0.03 | 0.02 | 0.03 | |
| Cluster-10108.2078 | 0.96 | 1.25 | 0.83 | 0.56 | 0.65 | 0.52 | 0.40 | 0.31 | 0.45 | |
| Cluster-10108.7191 | 1.00 | 1.01 | 0.98 | 3.77 | 3.83 | 3.57 | 17.33 | 18.13 | 18.47 | |
| Cluster-10108.4707 | 1.03 | 1.10 | 0.88 | 1.27 | 1.70 | 1.56 | 4.31 | 4.48 | 3.20 | |

# Table S4 Putative Genes Related to Flavonoid Biosynthesis and Transport

| gene_id | T1VST3 | T1VST4 | T3VST4 | BP Description |
| --- | --- | --- | --- | --- |
| Cluster-10108.10289 | -1.2992 | -0.73516 | 0.58075 | oxidation-reduction process//obsolete peroxidase reaction//response to oxidative stress |
| Cluster-10108.1496 | -1.7865 | -0.79795 | 1.0044 | obsolete peroxidase reaction//oxidation-reduction process//response to oxidative stress |
| Cluster-10108.151 | -2.7597 | -3.3453 | -0.56863 | oxidation-reduction process |
| Cluster-10108.24118 | 3.286 | 5.6974 | 2.4207 | obsolete peroxidase reaction//oxidation-reduction process//response to oxidative stress |
| Cluster-10108.10116 | -0.62616 | -1.6322 | -0.98798 | -- |
| Cluster-10108.5998 | 1.0642 | 5.8166 | 4.7751 | response to oxidative stress//oxidation-reduction process//obsolete peroxidase reaction |
| Cluster-6939.0 | -0.29112 | -4.6955 | -4.3868 | response to oxidative stress//oxidation-reduction process//obsolete peroxidase reaction |
| Cluster-10108.11022 | -2.2746 | -2.5863 | -0.29488 | oxidation-reduction process//fatty acid metabolic process//tryptophan metabolic process//fatty acid biosynthetic process//valine catabolic process//leucine catabolic process//benzoate metabolic process//isoleucine catabolic process//lysine catabolic process |
| Cluster-10108.15792 | -0.27109 | -1.1114 | -0.8222 | carbohydrate metabolic process |
| Cluster-10108.7191 | -2.9572 | -3.6703 | -0.69568 | -- |
| Cluster-10108.5903 | -2.3725 | -3.407 | -1.0155 | response to oxidative stress//obsolete peroxidase reaction//oxidation-reduction process |
| Cluster-10108.2912 | -1.9926 | -3.3742 | -1.3619 | obsolete peroxidase reaction//oxidation-reduction process//response to oxidative stress |
| Cluster-10108.7741 | 0.59847 | 1.4865 | 0.90659 | intracellular protein transport//carbohydrate metabolic process |
| Cluster-10108.15329 | 0.50711 | 2.28 | 1.7893 | isoleucine catabolic process//lysine catabolic process//benzoate metabolic process//leucine catabolic process//valine catabolic process//tryptophan metabolic process//fatty acid biosynthetic process//oxidation-reduction process//fatty acid metabolic process |
| Cluster-10108.15142 | -1.1394 | -1.1671 | -0.0101 | -- |
| Cluster-10108.11256 | -2.671 | -3.0917 | -0.40563 | regulation of translational initiation |
| Cluster-10108.18527 | -0.80277 | -1.3194 | -0.49939 | estrogen metabolic process//C21-steroid hormone metabolic process//steroid biosynthetic process//oxidation-reduction process//androgen metabolic process |
| Cluster-10108.22651 | 2.0072 | 2.7112 | 0.72034 | response to oxidative stress//obsolete peroxidase reaction//oxidation-reduction process |
| Cluster-5887.0 | -1.866 | -3.7965 | -1.9097 | oxidation-reduction process//obsolete peroxidase reaction//response to oxidative stress |
| Cluster-10108.26415 | -4.0404 | -5.2857 | -1.2282 | -- |
| Cluster-10108.4707 | -1.7592 | -2.8841 | -1.1079 | -- |
| Cluster-10108.17539 | 0.66012 | 1.4028 | 0.76055 | proteolysis |
| Cluster-10108.14637 | 0.74874 | 2.6994 | 1.9679 | oxidation-reduction process//obsolete peroxidase reaction//response to oxidative stress |
| Cluster-10108.10725 | 1.015 | 3.0296 | 2.0311 | carbohydrate metabolic process |
| Cluster-10108.17269 | -0.60947 | -1.0454 | -0.41746 | carbohydrate metabolic process |
| Cluster-10108.4368 | -1.3138 | -1.5418 | -0.20896 | obsolete peroxidase reaction//oxidation-reduction process//response to oxidative stress |
| Cluster-10108.7081 | -1.1468 | -1.341 | -0.17625 | carbohydrate metabolic process |
| Cluster-10108.4959 | 0.87581 | 1.8744 | 1.0163 | response to oxidative stress//cellular amino acid metabolic process//obsolete peroxidase reaction//oxidation-reduction process |
| Cluster-10108.24598 | 0.20659 | 4.3209 | 4.1364 | -- |
| Cluster-10108.9090 | -2.6256 | -3.4163 | -0.77226 | oxidation-reduction process |
| Cluster-10108.14282 | 4.5639 | 3.2126 | -1.3401 | N-terminal protein amino acid methylation |
| Cluster-10108.14128 | 1.5474 | 2.4274 | 0.89938 | regulation of transcription, DNA-templated//carbohydrate metabolic process |
| Cluster-10108.2772 | -2.0051 | -2.7974 | -0.77806 | -- |
| Cluster-10108.10182 | -0.61402 | -1.2035 | -0.5716 | regulation of translational initiation |
| Cluster-10108.17635 | 0.54194 | 1.4605 | 0.93861 | oxidation-reduction process//RNA modification//oxidative phosphorylation//carbohydrate metabolic process//ubiquitin-dependent protein catabolic process//pseudouridine synthesis//obsolete electron transport |
| Cluster-10108.2607 | -2.6731 | -3.3749 | -0.68186 | carbohydrate metabolic process |
| Cluster-10108.3019 | 0.35083 | 2.5365 | 2.2056 | carbohydrate metabolic process |
| Cluster-10108.13866 | 0.92574 | 1.7798 | 0.87286 | axon guidance//oxidation-reduction process |
| Cluster-10108.23873 | -0.53472 | 2.0763 | 2.6302 | response to oxidative stress//obsolete peroxidase reaction//porphyrin-containing compound biosynthetic process//oxidation-reduction process |
| Cluster-10108.1150 | -3.7279 | -4.7168 | -0.96472 | -- |
| Cluster-10108.1151 | -1.8899 | -2.9795 | -1.0744 | regulation of transcription, DNA-templated |
| Cluster-10108.10698 | 0.44465 | 1.4709 | 1.0433 | positive regulation of release of cytochrome c from mitochondria//interstrand cross-link repair//positive regulation of apoptotic process//oxidation-reduction process//intrinsic apoptotic signaling pathway |
| Cluster-10108.12563 | 0.77995 | 2.7721 | 2.0119 | response to oxidative stress//obsolete peroxidase reaction//oxidation-reduction process |
| Cluster-10108.22048 | 0.22338 | 3.5428 | 3.3383 | carbohydrate metabolic process |
| Cluster-10108.14491 | 0.81378 | 1.2266 | 0.4303 | -- |
| Cluster-10108.17220 | 0.56491 | 2.8499 | 2.3021 | -- |
| Cluster-10108.17188 | 3.7899 | 9.3056 | 5.5526 | cell adhesion involved in single-species biofilm formation |
| Cluster-10108.12481 | 0.40517 | 1.3563 | 0.96975 | response to oxidative stress//obsolete peroxidase reaction//oxidation-reduction process |
| Cluster-10108.3907 | -1.3481 | -1.8379 | -0.47369 | -- |
| Cluster-10108.9733 | 0.31491 | 1.5136 | 1.2159 | oxidation-reduction process |
| Cluster-10108.9613 | 1.3948 | 2.9417 | 1.5649 | obsolete acyl-carrier-protein biosynthetic process//fatty acid biosynthetic process |
| Cluster-10108.24606 | -2.1206 | -2.6675 | -0.53136 | -- |
| Cluster-10108.13424 | 0.059032 | 1.0123 | 0.96903 | obsolete acyl-carrier-protein biosynthetic process//fatty acid biosynthetic process |
| Cluster-10108.22335 | -2.3177 | -3.2101 | -0.87426 | -- |
| Cluster-10108.20907 | 1.2503 | 2.8072 | 1.5768 | -- |
| Cluster-10108.19497 | 1.5363 | 2.4145 | 0.89781 | regulation of transcription, DNA-templated//intracellular protein transport//endoplasmic reticulum to Golgi vesicle-mediated transport |
| Cluster-10108.7225 | -1.4617 | -2.36 | -0.87964 | androgen metabolic process//oxidation-reduction process//C21-steroid hormone metabolic process//steroid biosynthetic process//estrogen metabolic process |
| Cluster-10108.5296 | -2.2591 | -4.166 | -1.8869 | oxidation-reduction process |
| Cluster-10108.4110 | -0.61439 | 1.2116 | 1.8459 | obsolete acyl-carrier-protein biosynthetic process//fatty acid biosynthetic process |
| Cluster-10108.19098 | -0.34646 | -1.1305 | -0.76536 | -- |
| Cluster-10108.3905 | 0.04703 | -1.1751 | -1.2035 | -- |
| Cluster-10108.12273 | 3.7515 | 3.9721 | 0.23415 | oxidation-reduction process |
| Cluster-10108.2078 | -0.85304 | 1.8113 | 2.6853 | oxidation-reduction process |
| Cluster-10108.14936 | 0.77322 | 1.5735 | 0.81861 | oxidation-reduction process |
| Cluster-10108.11147 | -3.2321 | -1.2007 | 2.0499 | oxidation-reduction process//obsolete peroxidase reaction//response to oxidative stress |
| Cluster-10108.6728 | -0.69427 | 0.50751 | 1.2186 | -- |
| Cluster-10108.2287 | -3.6747 | -5.3904 | -1.6959 | -- |
| Cluster-10108.5342 | 5.1958 | 5.9287 | 0.74927 | -- |
| Cluster-10108.10713 | -1.8593 | -3.2511 | -1.3738 | multicellular organism development//cell differentiation |
| Cluster-10108.16559 | 2.7365 | 7.0024 | 4.2778 | nucleobase-containing compound metabolic process//hemolysis by symbiont of host erythrocytes//pyrimidine nucleobase metabolic process |
| Cluster-10108.16648 | 1.4323 | 5.554 | 4.1384 | protein phosphorylation |
| Cluster-10108.15494 | 1.0119 | 2.3252 | 1.3308 | DNA replication initiation//cellular biogenic amine metabolic process |
| Cluster-10108.14806 | 1.1615 | 2.3581 | 1.2165 | transmembrane transport//tryptophan metabolic process//transport//Mo-molybdopterin cofactor biosynthetic process//L-phenylalanine biosynthetic process//ion transport//tyrosine biosynthetic process//potassium ion transport//tryptophan biosynthetic process |
| Cluster-10108.3699 | 1.2132 | 2.1463 | 0.95139 | transport//protein glycosylation//proton transmembrane transport |
| Cluster-10108.6447 | 0.62052 | 1.3498 | 0.74818 | proton transmembrane transport//transport |

# Table S5 Genes Related to Signal Transduction

| gene_id | T1VST3 | T1VST4 | T3VST4 | BP Description |
| --- | --- | --- | --- | --- |
| Cluster-10108.3210 | 1.2004 | 1.8164 |  | signal transduction//hydrotropism//regulation of pH//transmembrane transport//cation transport//fusion of virus membrane with host plasma membrane |
| Cluster-10108.17417 | 3.336 | 2.9662 |  | signal transduction |
| Cluster-10108.16511 | 1.8475 | 2.7043 |  | signal transduction |
| Cluster-10108.9816 | 1.369 | 1.7864 |  | signal transduction |
| Cluster-10108.17545 | 2.6193 | 4.3424 | 1.7416 | signal transduction//antigen processing and presentation//immune response//intracellular protein transport//transmembrane transport//defense response to bacterium |
| Cluster-10108.24696 | 3.2415 | 5.8712 |  | sperm individualization |
| Cluster-10108.5635 | 2.0073 |  | -1.0626 | phosphorelay signal transduction system |
| Cluster-10108.11802 | 1.9813 | 2.1095 |  | signal transduction//regulation of transcription, DNA-templated//androgen receptor signaling pathway |
| Cluster-10108.14138 | 1.7834 | 2.2414 |  | signal transduction |
| Cluster-10108.12409 | 1.4437 | 4.1196 | 2.6931 | signal transduction//protein targeting |
| Cluster-10108.6552 | 1.9436 | 2.3421 |  | signal transduction//regulation of transcription, DNA-templated |
| Cluster-10108.6252 | 1.4479 | 1.4172 |  | signal transduction//regulation of transcription, DNA-templated |
| Cluster-10108.10643 | 4.2249 | 5.0016 |  | signal transduction |
| Cluster-10108.4282 | 2.8791 | 3.541 |  | signal transduction//DNA integration//cell population proliferation//growth |
| Cluster-10108.20195 | 2.0028 | 2.2701 |  | signal transduction//positive regulation of transcription, DNA-templated//potassium ion transport |
| Cluster-10108.8783 | 1.0238 | 1.4509 |  | signal transduction//DNA integration//transposition, DNA-mediated |
| Cluster-10108.11934 | 1.5098 | 2.5968 |  | signal transduction//phosphorelay signal transduction system//dsRNA transport//nucleobase-containing compound transport//phosphorylation |
| Cluster-10108.9873 | 1.0174 | 1.135 |  | signal transduction//ammonium transport |
| Cluster-10108.18325 | 1.3291 | 1.4346 |  | signal transduction//sensory perception of chemical stimulus |
| Cluster-10108.2827 | 2.7964 |  |  | signal transduction//mRNA transport//endoplasmic reticulum inheritance//bacterial-type flagellum-dependent cell motility//protein polymerization//cell adhesion//platelet activation |
| Cluster-10108.14185 | 1.1549 | 3.537 | 2.3997 | signal transduction//oxidation-reduction process |
| Cluster-10108.18085 | 1.407 | 1.0897 |  | cell population proliferation//T-helper 17 cell lineage commitment//signal transduction//growth//immune response |
| Cluster-10108.12231 | 3.5146 | 5.052 |  | cell population proliferation//signal transduction//immune response//growth |
| Cluster-10108.9320 | 3.1783 | 5.4021 |  | double-strand break repair//signal transduction//platelet activation//cell motility//mRNA splicing, via spliceosome//protein polymerization |
| Cluster-10108.6035 | 1.6469 | 2.4242 |  | RNA catabolic process//DNA repair//signal transduction//chemotaxis//meiotic chromosome segregation |
| Cluster-10108.9656 | 2.8697 | 3.5086 |  | mitochondrial electron transport, cytochrome c to oxygen |
| Cluster-10108.7909 | 1.1427 | 1.3497 |  | mitochondrial electron transport, cytochrome c to oxygen |
| Cluster-10108.6572 | 6.1658 |  |  | pathogenesis//transport//mitochondrial electron transport, cytochrome c to oxygen |
| Cluster-10108.14303 | 1.0923 |  |  | androgen receptor signaling pathway//regulation of transcription, DNA-templated//signal transduction//female sex differentiation//mRNA processing//transcription initiation from RNA polymerase II promoter |
| Cluster-10108.11715 | 1.0874 | 1.1862 |  | intracellular signal transduction//regulation of transcription, DNA-templated |
| Cluster-10108.20923 | 1.1771 |  |  | intracellular signal transduction//oxidation-reduction process |
| Cluster-10108.22109 | 1.1044 | 1.4767 |  | intracellular signal transduction//transcription, DNA-templated |
| Cluster-10108.11140 | 3.1909 | 5.5892 | 2.4204 | phosphorelay signal transduction system//mitochondrial electron transport, cytochrome c to oxygen//microtubule-based movement//cell motility//cell adhesion//platelet activation//DNA catabolic process//signal transduction//microtubule-based process//protein polymerization//phosphorylation |
| Cluster-10108.21886 | 3.7253 | 5.8701 |  | multicellular organism development |
| Cluster-10108.10806 | 1.6672 | 1.9459 |  | malate transport//negative regulation of phosphoprotein phosphatase activity//regulation of response to reactive oxygen species//response to light stimulus//signal transduction//protein transport |
| Cluster-10108.14650 | 3.9462 | 5.6847 |  | G protein-coupled receptor signaling pathway |
| Cluster-10108.7139 | 1.7103 | 2.2997 |  | cell surface receptor signaling pathway |
| Cluster-10108.23408 | 2.5042 | 2.4592 |  | cell surface receptor signaling pathway |
| Cluster-10108.11260 | 1.0938 |  |  | DNA-templated transcription, termination//DNA replication//signal transduction//regulation of female receptivity, post-mating |
| Cluster-10108.6994 | 2.0204 | 2.4388 |  | cell population proliferation//signal transduction//growth |
| Cluster-10108.17994 | 1.4646 | 1.9385 |  | chemical synaptic transmission//signal transduction//proteolysis |
| Cluster-10108.19061 | 1.7572 | 2.2598 |  | G protein-coupled receptor signaling pathway |
| Cluster-10108.7725 | 1.2821 | 4.0138 | 2.7528 | G protein-coupled receptor signaling pathway |
| Cluster-10108.15373 | 1.6629 | 2.2866 |  | multicellular organism development//signal transduction//protein secretion//sensory organ development |
| Cluster-10108.6754 | 1.6776 | 3.0034 |  | immune response//growth//signal transduction//T-helper 17 cell lineage commitment//cell population proliferation |
| Cluster-10108.16092 | 1.2482 | 2.8582 | 1.6281 | neuronal signal transduction |
| Cluster-10108.9032 | 1.6573 | 2.7541 |  | protein secretion by the type II secretion system//signal transduction//neuropeptide signaling pathway |
| Cluster-10108.3653 | 3.7832 | 5.9231 |  | T cell receptor signaling pathway//growth//cell population proliferation//signal transduction//B cell receptor signaling pathway |
| Cluster-10108.4670 | 1.263 | 2.3469 |  | fusion of virus membrane with host plasma membrane//signal transduction |
| Cluster-10108.9625 | -3.7404 | -3.2047 |  | signal transduction//immune response//oxidation-reduction process//chemotaxis |
| Cluster-10108.17444 | -3.4493 | -2.2412 |  | signal transduction//cell population proliferation//gonad development//oxidation-reduction process//growth |
| Cluster-10108.15090 | -1.9995 | -2.7701 |  | signal transduction//ATP synthesis coupled proton transport//phosphorelay signal transduction system |
| Cluster-10108.20481 | -5.1657 | -6.0486 |  | signal transduction//chemotaxis |
| Cluster-10108.20801 | -2.9304 | -2.4808 |  | signal transduction |
| Cluster-10108.18549 | -2.0553 | -3.1158 | -1.0427 | signal transduction |
| Cluster-10108.14230 | -1.8094 | -2.3792 |  | signal transduction//receptor-mediated endocytosis |
| Cluster-10108.10896 | -1.149 | -1.6056 |  | signal transduction |
| Cluster-10108.10222 | -1.2243 | -1.8815 |  | signal transduction//protein phosphorylation |
| Cluster-10108.24714 | -6.9816 | -9.6369 | -2.6355 | signal transduction |
| Cluster-10108.4088 | -2.1394 | -2.4108 |  | signal transduction |
| Cluster-10108.20777 | -1.8306 | -2.3071 |  | signal transduction//protein phosphorylation |
| Cluster-10108.11057 | -1.3241 | -2.1003 |  | signal transduction//protein phosphorylation |
| Cluster-10108.1393 | -2.445 | -3.3338 |  | signal transduction//protein phosphorylation |
| Cluster-10108.222 | -6.925 | -8.588 |  | signal transduction//regulation of transcription by RNA polymerase II//lipid metabolic process |
| Cluster-10108.7024 | -2.0916 |  |  | signal transduction |
| Cluster-10108.17019 | -1.1297 | -1.3914 |  | signal transduction//regulation of transcription, DNA-templated//protein trimerization |
| Cluster-10108.7542 | -2.8206 | -2.9264 |  | signal transduction//response to light stimulus |
| Cluster-10108.4712 | -3.2942 | -5.1913 |  | signal transduction//regulation of transcription, DNA-templated//androgen receptor signaling pathway//transmembrane transport |
| Cluster-10108.5408 | -1.2104 | -1.5302 |  | signal transduction |
| Cluster-10108.25085 | -2.5653 | -2.3995 |  | signal transduction |
| Cluster-10108.534 | -2.3517 | -4.1698 | -1.8056 | signal transduction//malate transport//phosphorelay signal transduction system//phosphorylation |
| Cluster-10108.12457 | -1.063 | -1.7127 |  | signal transduction//amino acid transport//regulation of cell adhesion//mitotic spindle assembly checkpoint//regulation of cell migration//regulation of embryonic development |
| Cluster-10108.29127 | -2.6863 |  |  | signal transduction//transmembrane transport//metal ion transport |
| Cluster-10108.13689 | -1.6679 | -2.493 |  | regulation of transcription, DNA-templated//signal transduction//ubiquitin-dependent protein catabolic process |
| Cluster-10108.15359 | -1.2155 | -1.6452 |  | regulation of transcription, DNA-templated//signal transduction//protein phosphorylation |
| Cluster-10108.12343 | -1.6654 | -1.5756 |  | neuronal signal transduction |
| Cluster-10108.2965 | -3.4939 | -4.2335 |  | neuronal signal transduction |
| Cluster-10108.11762 | -2.1866 | -2.2079 |  | -- |
| Cluster-10108.14164 | -1.0003 | -1.1719 |  | transmembrane transport |
| Cluster-10108.12118 | -2.5558 | -2.4371 |  | G protein-coupled receptor signaling pathway |
| Cluster-10108.8709 | -1.5667 | -2.4605 |  | G protein-coupled receptor signaling pathway//potassium ion transport//oxidation-reduction process |
| Cluster-10108.6166 | -1.3503 | -1.2632 |  | G protein-coupled receptor signaling pathway//sensory perception of chemical stimulus//response to pheromone |
| Cluster-10108.24747 | -1.8379 | -1.9279 |  | G protein-coupled receptor signaling pathway//response to pheromone//sensory perception of chemical stimulus |
| Cluster-10108.16815 | -2.5218 | -2.9546 |  | G protein-coupled receptor signaling pathway//transmembrane transport//transport |
| Cluster-10108.22481 | -1.1568 |  |  | G protein-coupled receptor signaling pathway//sensory perception of chemical stimulus//response to pheromone |
| Cluster-10108.27839 | -5.1148 | -4.8345 |  | G protein-coupled receptor signaling pathway//heme transport |
| Cluster-10108.17125 | -1.7917 | -1.8417 |  | cell population proliferation//signal transduction//growth |
| Cluster-10108.5018 | -1.41 | -1.5834 |  | cell population proliferation//signal transduction//growth |
| Cluster-10108.16583 | -1.4853 | -1.8926 |  | protein phosphorylation//signal transduction//fusion of virus membrane with host plasma membrane |
| Cluster-10108.15723 | -2.4786 | -3.3054 |  | regulation of viral process//signal transduction//sensory perception of chemical stimulus |
| Cluster-10108.8115 | -3.8427 | -5.0016 | -1.1412 | pheromone-dependent signal transduction involved in conjugation with cellular fusion//signal transduction |
| Cluster-10108.7523 | -3.1631 | -4.686 | -1.5052 | regulation of female receptivity, post-mating//signal transduction |
| Cluster-10108.17120 | -1.2461 | -1.9694 |  | attachment of spindle microtubules to kinetochore//signal transduction//neuronal signal transduction |
| Cluster-10108.7163 | -1.327 | -2.0417 |  | response to light stimulus//signal transduction |
| Cluster-10108.26657 | -6.358 | -7.5149 |  | protein maturation//intracellular signal transduction |
| Cluster-10108.6362 | -3.5475 | -5.6658 | -2.101 | intracellular signal transduction//G protein-coupled receptor signaling pathway |
| Cluster-10108.3042 | -2.0851 |  | 1.7952 | intracellular signal transduction |
| Cluster-10108.2332 | -1.9832 |  | 2.225 | intracellular signal transduction |
| Cluster-10108.6877 | -1.6498 | -2.6374 |  | intracellular signal transduction |
| Cluster-10108.22140 | -1.6908 | -1.9587 |  | intracellular signal transduction//protein ubiquitination |
| Cluster-10108.6674 | -1.6432 | -2.3226 |  | phosphorelay signal transduction system |
| Cluster-10108.15145 | -1.1272 | -1.672 |  | phosphorelay signal transduction system |
| Cluster-10108.15826 | -1.2881 | -1.9562 |  | phosphorelay signal transduction system//neuronal signal transduction |
| Cluster-10108.7465 | -1.3372 | -2.3873 | -1.0317 | phosphorelay signal transduction system//protein phosphorylation//protein dephosphorylation//phosphorylation |
| Cluster-10108.14301 | -1.6113 | -2.1983 |  | phosphorelay signal transduction system//ribosome biogenesis |
| Cluster-10108.2389 | -2.1406 | -2.8335 |  | phosphorelay signal transduction system//DNA replication initiation//regulation of carbohydrate metabolic process//phosphorylation |
| Cluster-10108.27216 | -3.8206 | -5.0244 |  | phosphorelay signal transduction system |
| Cluster-10108.2516 | -4.3583 | -6.3171 |  | phosphorelay signal transduction system |
| Cluster-10108.24679 | -3.427 | -4.3687 |  | phosphorelay signal transduction system//phosphorylation//protein dephosphorylation |
| Cluster-10108.17701 | -1.3966 | -1.8224 |  | immune response//signal transduction//inflammatory response |
| Cluster-10108.4279 | -2.418 | -2.971 |  | cell population proliferation//phosphorelay signal transduction system//signal transduction//immune response//protein neddylation//growth |
| Cluster-10108.19036 | -1.3662 | -2.1969 |  | attachment of spindle microtubules to kinetochore//phosphorelay signal transduction system |
| Cluster-10108.12907 | -1.3957 | -2.0188 |  | negative regulation of double-strand break repair via nonhomologous end joining//signal transduction |
| Cluster-10108.20294 | -1.0234 | -2.1042 | -1.0618 | cell septum assembly//signal transduction//FtsZ-dependent cytokinesis//intra-Golgi vesicle-mediated transport |
| Cluster-10108.4587 | -3.6956 | -4.2058 |  | Wnt signaling pathway |
| Cluster-10108.6542 | -1.5676 | -1.8917 |  | Wnt signaling pathway//DNA integration |
| Cluster-10108.27797 | -2.722 |  |  | spermatogenesis |
| Cluster-10108.20356 | -2.2468 | -2.1391 |  | detection of chemical stimulus involved in sensory perception of taste//obsolete taste perception//G protein-coupled receptor signaling pathway, coupled to cyclic nucleotide second messenger//DNA integration |
| Cluster-10108.8778 | -1.1702 | -1.7247 |  | tachykinin receptor signaling pathway |
| Cluster-10108.22908 | -1.6529 | -2.3818 |  | androgen receptor signaling pathway//signal transduction//positive regulation of Golgi to plasma membrane protein transport//regulation of transcription, DNA-templated |
| Cluster-10108.19210 | -1.2952 | -3.0355 | -1.7218 | nitrogen fixation//G protein-coupled receptor signaling pathway//cell redox homeostasis |
| Cluster-10108.22333 | -1.1126 |  |  | antigen processing and presentation//immune response//signal transduction//intracellular protein transport |
| Cluster-10108.18231 | -1.0268 | -1.2347 |  | signal peptide processing |
| Cluster-10108.18108 | -1.1878 | -2.3951 |  | regulation of transcription by RNA polymerase II//phosphorelay signal transduction system |
| Cluster-10108.21291 | -1.4471 | -2.5504 |  | cell communication//signal transduction |
| Cluster-10108.17974 | | -1.0414 |  | intracellular signal transduction//phospholipid catabolic process//signal transduction//lipid metabolic process//transcription by RNA polymerase III//diacylglycerol metabolic process |
| Cluster-10108.26266 | | | 2.9507 | intracellular signal transduction |
| Cluster-10108.10840 | | 1.4122 |  | intracellular signal transduction |
| Cluster-10108.14628 | | 1.0138 |  | intracellular signal transduction |
| Cluster-10108.12003 | | 1.495 |  | intracellular signal transduction |
| Cluster-10108.15820 | | 1.0295 |  | intracellular signal transduction//negative regulation of T cell activation |
| Cluster-10108.4612 | | -3.0122 |  | intracellular signal transduction |
| Cluster-385.0 | | -6.4925 |  | G protein-coupled receptor signaling pathway |
| Cluster-10108.14994 | | 1.4588 |  | G protein-coupled receptor signaling pathway |
| Cluster-10108.9162 | | 1.1094 |  | G protein-coupled receptor signaling pathway |
| Cluster-10108.17117 | | 1.631 |  | G protein-coupled receptor signaling pathway |
| Cluster-10108.16089 | | 2.866 | 3.7514 | G protein-coupled receptor signaling pathway |
| Cluster-10108.5969 | | -1.2418 |  | G protein-coupled receptor signaling pathway |
| Cluster-10108.17840 | | 1.1684 |  | G protein-coupled receptor signaling pathway |
| Cluster-10108.12837 | | 1.3633 |  | G protein-coupled receptor signaling pathway//cellular amino acid biosynthetic process//cellular biogenic amine metabolic process |
| Cluster-10108.4868 | | 1.0955 | 1.7132 | G protein-coupled receptor signaling pathway//regulation of transcription, DNA-templated |
| Cluster-10108.9118 | | -1.3639 |  | G protein-coupled receptor signaling pathway//response to pheromone |
| Cluster-10108.13357 | | -1.0931 |  | G protein-coupled receptor signaling pathway//cellular biogenic amine metabolic process |
| Cluster-10108.19042 | | 1.9221 |  | G protein-coupled receptor signaling pathway, coupled to cyclic nucleotide second messenger//sensory perception of smell |
| Cluster-10108.11374 | | -1.6319 |  | G protein-coupled receptor signaling pathway//telomere maintenance//sensory perception of taste |
| Cluster-10108.10077 | | 2.1065 | 1.4475 | G protein-coupled receptor signaling pathway//response to pheromone |
| Cluster-10108.18494 | | -1.0086 |  | G protein-coupled receptor signaling pathway |
| Cluster-10108.8906 | | 1.9137 | 1.6657 | G protein-coupled receptor signaling pathway//amino acid transmembrane transport |
| Cluster-10108.3752 | | 1.8885 |  | G protein-coupled receptor signaling pathway |
| Cluster-10108.775 | | -6.1191 |  | G protein-coupled receptor signaling pathway |
| Cluster-10108.23034 | | -3.0296 |  | signal transduction |
| Cluster-10108.8024 | | -1.2708 |  | signal transduction |
| Cluster-10108.6519 | | 1.314 |  | signal transduction |
| Cluster-10108.22669 | | 2.9347 |  | signal transduction |
| Cluster-10108.16708 | | 1.8811 | 2.1176 | signal transduction//estrogen metabolic process//oxidation-reduction process//androgen metabolic process//C21-steroid hormone metabolic process//steroid biosynthetic process |
| Cluster-10108.4477 | | 2.9122 |  | signal transduction//regulation of transcription, DNA-templated |
| Cluster-10108.20585 | | 2.7755 | 2.2141 | signal transduction//protein phosphorylation |
| Cluster-10108.11538 | | -1.3893 |  | signal transduction//biosynthetic process |
| Cluster-10108.15907 | | 1.9794 | 1.8801 | signal transduction//regulation of transcription, DNA-templated//androgen receptor signaling pathway |
| Cluster-10108.15298 | | 1.2627 |  | signal transduction |
| Cluster-10108.713 | | -1.5007 |  | signal transduction |
| Cluster-10108.1241 | | -2.7876 |  | signal transduction |
| Cluster-10108.12707 | | 1.2795 |  | signal transduction//transmembrane transport//platelet activation//CAAX-box protein processing//protein polymerization//proteolysis |
| Cluster-10108.17044 | | -1.2893 |  | signal transduction//fusion of virus membrane with host plasma membrane |
| Cluster-9298.0 | | 4.8362 |  | signal transduction |
| Cluster-10108.13014 | | 1.7182 | 1.1482 | signal transduction |
| Cluster-10108.16124 | | 1.0913 |  | signal transduction//neuronal signal transduction |
| Cluster-10108.20589 | | 2.4247 |  | signal transduction |
| Cluster-10108.13878 | | 1.652 | 1.1408 | signal transduction//cell population proliferation//lipid biosynthetic process//growth//oxidation-reduction process//chitin catabolic process |
| Cluster-10108.17087 | | 1.7991 |  | signal transduction |
| Cluster-10108.20415 | | 1.3525 |  | signal transduction |
| Cluster-10108.21088 | | 1.5291 |  | signal transduction |
| Cluster-10108.3274 | | 3.7182 |  | signal transduction |
| Cluster-10108.13077 | | 1.3017 |  | signal transduction//regulation of female receptivity, post-mating |
| Cluster-10108.3010 | | 1.4582 |  | signal transduction |
| Cluster-10108.660 | | -1.848 |  | signal transduction |
| Cluster-10108.4708 | | 1.3673 |  | -- |
| Cluster-10108.4029 | | -1.5872 |  | Wnt signaling pathway |
| Cluster-10108.7728 | | -2.2587 |  | Wnt signaling pathway//response to auxin |
| Cluster-10108.14880 | | -1.3287 |  | Wnt signaling pathway |
| Cluster-10108.20157 | | -2.7463 |  | mRNA splicing, via spliceosome//regulation of store-operated calcium entry |
| Cluster-10108.17437 | | | 1.271 | regulation of immune response//protein phosphorylation//protein dephosphorylation//phosphorylation//positive regulation of phosphatidylinositol 3-kinase signaling//signal transduction//phosphorelay signal transduction system |
| Cluster-10108.22071 | | -1.7599 |  | lymphocyte activation |
| Cluster-10108.3453 | | -1.4531 |  | neuropeptide signaling pathway |
| Cluster-10108.13236 | | 1.561 |  | neuropeptide signaling pathway |
| Cluster-10108.10601 | | -1.189 |  | neuropeptide signaling pathway//oxidation-reduction process//tetrapyrrole biosynthetic process |
| Cluster-10108.29061 | | -2.5499 |  | neuropeptide signaling pathway |
| Cluster-10108.7573 | | -1.1907 |  | neuropeptide signaling pathway//protein ubiquitination |
| Cluster-10108.4390 | | -1.7149 |  | neuropeptide signaling pathway |
| Cluster-10108.10104 | | -1.9519 |  | smoothened signaling pathway//neuronal signal transduction |
| Cluster-10108.383 | | -4.148 | -2.469 | smoothened signaling pathway//defense response |
| Cluster-10108.18131 | | -1.1403 |  | smoothened signaling pathway |
| Cluster-10108.12537 | | -1.287 |  | SRP-dependent cotranslational protein targeting to membrane |
| Cluster-10108.21960 | | -1.605 |  | phosphorelay signal transduction system |
| Cluster-10108.16130 | | -1.472 |  | phosphorelay signal transduction system//signal transduction//phosphorylation |
| Cluster-10108.5006 | | -1.2194 |  | phosphorelay signal transduction system//DNA topological change |
| Cluster-10108.8003 | | -1.3248 |  | phosphorelay signal transduction system//regulation of transcription, DNA-templated//DNA damage checkpoint |
| Cluster-10108.8367 | | 3.6435 |  | phosphorelay signal transduction system |
| Cluster-10108.29148 | | -4.0526 |  | phosphorelay signal transduction system |
| Cluster-10108.13169 | | 1.2756 |  | phosphorelay signal transduction system//signal transduction//regulation of transcription, DNA-templated//phosphorylation//photosynthesis, dark reaction//bacteriochlorophyll biosynthetic process |
| Cluster-10108.19021 | | -1.5313 |  | phosphorelay signal transduction system |
| Cluster-10108.18559 | | 1.1373 | 1.5664 | phosphorelay signal transduction system//protein dephosphorylation//phosphorylation |
| Cluster-10108.18825 | | -1.5999 |  | signal peptide processing//ion transport//transmembrane transport |
| Cluster-10108.10349 | | 1.5189 |  | signal peptide processing |
| Cluster-10108.11306 | | 2.3619 |  | signal peptide processing |
| Cluster-10108.2152 | | -6.4976 |  | signal peptide processing |
| Cluster-10108.18042 | | -1.2695 |  | signal peptide processing//carbohydrate metabolic process |
| Cluster-10108.16281 | | -1.0405 |  | signal peptide processing//cell septum assembly//regulation of transcription, DNA-templated//intracellular protein transport//FtsZ-dependent cytokinesis |
| Cluster-10108.29526 | | -3.3166 |  | B cell activation//immune response-regulating signaling pathway |
| Cluster-10108.20736 | | 2.4406 |  | Group I intron splicing//signal transduction |
| Cluster-10108.12039 | | 1.1318 |  | phosphorylation//phosphorelay signal transduction system//signal transduction |
| Cluster-10108.21814 | | -1.2362 |  | protein dephosphorylation//phosphorylation//phosphorelay signal transduction system |
| Cluster-10108.20478 | | -3.0429 | -2.0925 | regulation of transcription, DNA-templated//phosphorelay signal transduction system//phosphorylation |
| Cluster-10108.12569 | | 1.2482 |  | phosphorylation//transcription, DNA-templated//phosphorelay signal transduction system//signal transduction |
| Cluster-10108.13196 | | 1.2049 |  | phosphorylation//regulation of carbohydrate metabolic process//isoleucine catabolic process//beta-alanine metabolic process//leucine catabolic process//phosphorelay signal transduction system//valine catabolic process |
| Cluster-10108.9814 | | 1.3759 |  | chemotaxis//signal transduction//intracellular protein transport |
| Cluster-10108.17977 | | -1.1187 |  | tRNA threonylcarbamoyladenosine modification//pheromone-dependent signal transduction involved in conjugation with cellular fusion//signal transduction |
| Cluster-10108.13574 | | 1.6272 | 1.1007 | lipid homeostasis//signal transduction |
| Cluster-10108.14517 | | -1.2215 |  | proteolysis//signal transduction |
| Cluster-10108.4758 | | -1.1654 |  | feeding behavior//neuropeptide signaling pathway |
| Cluster-10108.12554 | | -1.3489 |  | regulation of female receptivity, post-mating//signal transduction//pathogenesis |
| Cluster-10108.17560 | | 1.3162 |  | regulation of female receptivity, post-mating//signal transduction |
| Cluster-10108.17265 | | -1.5754 |  | regulation of female receptivity, post-mating//signal transduction//transcription, DNA-templated |
| Cluster-10108.16792 | | 2.8323 | 2.4342 | heme transport//signal transduction |
| Cluster-10108.6735 | | 1.5191 |  | innate immune response//transmembrane transport//intracellular transport//signal transduction |
| Cluster-10108.2942 | | -2.0934 | -1.2763 | protein homooligomerization//signal transduction |
| Cluster-10108.20354 | | -1.0954 |  | purine nucleobase metabolic process//purine nucleotide biosynthetic process//GMP biosynthetic process//glutamate metabolic process//multicellular organism development//signal transduction//regulation of transcription, DNA-templated//ubiquitin-dependent protein catabolic process |
| Cluster-10108.17115 | | 1.1901 | 1.0557 | chorion-containing eggshell formation//nodule morphogenesis//smoothened signaling pathway//kinetochore assembly//intracellular transport of virus//chromosome segregation |
| Cluster-10108.9127 | | -1.0658 |  | regulation of DNA-templated transcription, termination//protein prenylation//signal transduction//transcriptional attenuation by ribosome |
| Cluster-10108.18793 | | 1.0599 |  | protein prenylation |
| Cluster-10108.10561 | | 1.0599 |  | protein prenylation//signal transduction |
| Cluster-10108.17495 | | 1.9752 | 1.0526 | immune response//transcription initiation from RNA polymerase II promoter//chemotaxis//signal transduction |
| Cluster-10108.21283 | | -1.7229 |  | cellular amino acid biosynthetic process//neuronal signal transduction//methionine biosynthetic process//sensory perception of taste//transcription by RNA polymerase III |
| Cluster-10108.15450 | | -2.0623 | -1.2636 | negative regulation of transcription, DNA-templated//neuropeptide signaling pathway |
| Cluster-10108.12342 | | 1.3819 |  | receptor clustering//G protein-coupled acetylcholine receptor signaling pathway//ribosome biogenesis//translation |
| Cluster-10108.13726 | | -1.0022 |  | lipoprotein biosynthetic process//signal transduction//sensory perception of chemical stimulus |
| Cluster-10108.22156 | | -1.1119 |  | growth//cell population proliferation//signal transduction |
| Cluster-10108.28134 | | -2.4462 |  | cell population proliferation//signal transduction//growth |
| Cluster-10108.14689 | | 1.222 |  | defense response to other organism |
| Cluster-10108.10688 | | 1.0272 |  | hormone-mediated signaling pathway//signal transduction |
| Cluster-10108.1287 | | -3.6669 |  | tachykinin receptor signaling pathway |
| Cluster-10108.4247 | |  | 1.3633 | sucrose metabolic process//signal transduction//starch metabolic process//UDP-glucose metabolic process//cellulose biosynthetic process//archaeal or bacterial-type flagellum-dependent cell motility |
| Cluster-10108.11732 | | -1.0875 |  | mitigation of host immune response by virus |

# Table S6 Genes Related to Carbohydrate and Energy Metabolism

| gene_id | T1VST3 | T1VST4 | T3VST4 | BP Description |
| --- | --- | --- | --- | --- |
| Cluster-10108.10135 | 1.3063 | 1.7547 |  | carbohydrate metabolic process |
| Cluster-10108.15280 | 1.9377 | 2.7696 |  | cellular glucan metabolic process//carbohydrate metabolic process |
| Cluster-10108.10482 | 2.9982 | 3.5204 |  | mitotic spindle organization//carbohydrate metabolic process |
| Cluster-10108.16870 | 3.5131 | 8.3 | 4.8151 | carbohydrate metabolic process |
| Cluster-10108.4823 | 3.5724 | 5.6849 |  | protein homooligomerization//DNA repair//carbohydrate metabolic process |
| Cluster-10108.18355 | 1.8263 | 2.4375 |  | carbohydrate metabolic process |
| Cluster-10108.13519 | 1.2879 | 2.3274 | 1.0583 | carbohydrate metabolic process |
| Cluster-10108.16123 | 1.5326 | 2.6517 | 1.1376 | carbohydrate metabolic process |
| Cluster-10108.14128 | 1.5474 | 2.4274 |  | regulation of transcription, DNA-templated//carbohydrate metabolic process |
| Cluster-10108.9037 | 2.0948 | 3.2206 |  | vacuolar proton-transporting V-type ATPase complex assembly//carbohydrate metabolic process |
| Cluster-10108.16849 | 2.704 | 4.17 |  | carbohydrate metabolic process |
| Cluster-10108.15962 | 1.8526 | 1.6015 |  | carbohydrate metabolic process//purine nucleobase metabolic process//proteolysis |
| Cluster-10108.15618 | 3.7958 | 8.0031 |  | carbohydrate metabolic process//cellular glucan metabolic process |
| Cluster-10108.13244 | 1.2485 | 1.9973 |  | carbohydrate metabolic process//signal transduction |
| Cluster-10108.17738 | 3.973 | 8.3775 | 4.4158 | photosynthesis//carbohydrate metabolic process |
| Cluster-10108.19285 | 1.7489 | 2.9979 | 1.2692 | carbohydrate metabolic process |
| Cluster-10108.11120 | 1.1048 | 3.3618 | 2.2741 | carbohydrate metabolic process |
| Cluster-10108.11644 | 3.5364 | 7.2921 | 3.7845 | carbohydrate metabolic process |
| Cluster-10108.9287 | 1.052 | 1.6108 |  | carbohydrate metabolic process |
| Cluster-10108.13521 | 2.8375 | 6.351 | 3.5354 | plasma membrane fusion involved in cytogamy//carbohydrate metabolic process |
| Cluster-10108.20619 | 2.2126 | 2.0213 |  | peptidoglycan turnover//carbohydrate metabolic process |
| Cluster-10108.14384 | 2.079 | 5.8378 | 3.7762 | proteasome assembly//carbohydrate metabolic process |
| Cluster-10108.8810 | 1.2852 | 2.1531 |  | carbohydrate metabolic process |
| Cluster-10108.16665 | 1.5826 | 3.6867 | 2.1204 | glycosaminoglycan catabolic process//carbohydrate metabolic process//glycosphingolipid metabolic process//galactose metabolic process//glycerolipid metabolic process |
| Cluster-10108.9442 | 1.7602 | 3.2697 |  | carbohydrate metabolic process |
| Cluster-10108.13012 | 2.9451 | 6.2852 |  | cellular glucan metabolic process//carbohydrate metabolic process//endoplasmic reticulum to Golgi vesicle-mediated transport//intracellular protein transport |
| Cluster-10108.19412 | 1.1614 | 3.5132 | 2.3709 | carbohydrate metabolic process//ATP synthesis coupled proton transport//cellular glucan metabolic process |
| Cluster-10108.17704 | 1.9489 | 4.2766 | 2.3482 | photosynthesis, light reaction//carbohydrate metabolic process//obsolete electron transport |
| Cluster-10108.7932 | 1.3367 | 1.1538 |  | folic acid-containing compound biosynthetic process//carbohydrate metabolic process//carbohydrate derivative metabolic process//glyoxylate metabolic process |
| Cluster-10108.13016 | 1.0789 | 1.9833 |  | amine metabolic process//oxidation-reduction process//carbohydrate metabolic process |
| Cluster-10108.12375 | 1.1459 | 2.8358 | 1.7099 | carbohydrate metabolic process |
| Cluster-10108.12081 | 1.1786 | 2.5554 | 1.3955 | carbohydrate metabolic process |
| Cluster-10108.4457 | 1.7823 | 2.8066 |  | carbohydrate metabolic process |
| Cluster-10108.6226 | 1.6146 | 2.6648 |  | glycosaminoglycan catabolic process//carbohydrate metabolic process//galactose metabolic process//glycosphingolipid metabolic process//glycerolipid metabolic process |
| Cluster-10108.9391 | 1.353 | 2.2876 |  | carbohydrate metabolic process//oxidation-reduction process//lysine biosynthetic process via diaminopimelate//carbohydrate catabolic process//lysine biosynthetic process |
| Cluster-10108.20153 | 2.1086 | 1.518 |  | polysaccharide catabolic process//viral DNA genome replication//carbohydrate metabolic process//proteolysis |
| Cluster-10108.10725 | 1.015 | 3.0296 | 2.0311 | carbohydrate metabolic process |
| Cluster-10108.9895 | 1.2184 | 2.8331 | 1.6358 | carbohydrate metabolic process |
| Cluster-10108.19416 | 1.573 | 2.654 |  | regulation of translation//carbohydrate metabolic process |
| Cluster-10108.11890 | 1.0156 | 2.4949 | 1.4983 | carbohydrate metabolic process |
| Cluster-8937.0 | 2.3298 |  |  | carbohydrate metabolic process//signal transduction |
| Cluster-10108.9124 | 1.578 | 4.0792 | 2.5201 | carbohydrate metabolic process |
| Cluster-8079.0 | 5.4247 | 5.3693 |  | carbohydrate metabolic process |
| Cluster-10108.13428 | 1.3899 | 2.0402 |  | defense response |
| Cluster-10108.19188 | 2.3651 | 3.4133 |  | -- |
| Cluster-10108.16339 | 2.0817 | 2.9717 |  | -- |
| Cluster-10108.18600 | 1.6011 | 2.7133 |  | -- |
| Cluster-10108.10108 | 1.3438 | 2.5521 |  | -- |
| Cluster-10108.10883 | 1.1789 | 1.3997 |  | regulation of transcription, DNA-templated//photosynthesis |
| Cluster-10108.11654 | 1.7351 | 1.9195 |  | butyrate metabolic process |
| Cluster-10108.13420 | 2.5333 | 3.5424 |  | carbohydrate metabolic process |
| Cluster-10108.15636 | 1.3906 | 2.7588 | 1.3876 | carbohydrate metabolic process |
| Cluster-10108.20241 | 2.5327 | 3.4944 |  | carbohydrate metabolic process |
| Cluster-10108.10487 | 1.8685 | 2.2354 |  | carbohydrate metabolic process |
| Cluster-10108.18606 | 1.9019 | 2.4218 |  | carbohydrate metabolic process |
| Cluster-10108.8059 | 1.3516 | 1.5811 |  | carbohydrate metabolic process |
| Cluster-10108.15269 | 2.0715 | 1.9869 |  | sucrose metabolic process//vesicle-mediated transport//signal transduction//starch metabolic process//trehalose catabolic process//trehalose metabolic process |
| Cluster-10108.12785 | 2.0564 | 2.2791 |  | cell redox homeostasis//proteolysis//obsolete electron transport |
| Cluster-10108.8406 | 2.8266 | 3.3744 |  | DNA replication//pyrimidine nucleobase metabolic process//oxidation-reduction process//purine nucleobase metabolic process//deoxyribonucleoside diphosphate metabolic process |
| Cluster-10108.12306 | 1.3813 | 1.496 |  | oxidation-reduction process//proteolysis//androgen metabolic process//steroid biosynthetic process//carbohydrate biosynthetic process//C21-steroid hormone metabolic process//potassium ion transport//protein deubiquitination//lysine biosynthetic process//lysine biosynthetic process via diaminopimelate//estrogen metabolic process |
| Cluster-10108.16023 | 1.0041 | 1.4984 |  | fructose metabolic process//mannose metabolic process//gluconeogenesis//inositol metabolic process//glycolytic process//carbon utilization//pentose-phosphate shunt |
| Cluster-10108.19578 | 1.378 |  |  | photosynthesis |
| Cluster-10108.11027 | 3.3901 | 3.6372 |  | photosynthesis |
| Cluster-10108.3355 | 2.84 | 3.1861 |  | photosynthesis |
| Cluster-10108.11254 | 1.5081 | 3.4834 | 1.9925 | photosynthesis |
| Cluster-10108.7931 | 1.092 | 2.0852 |  | photosynthesis |
| Cluster-10108.21401 | 1.1348 | 1.7616 |  | photosynthesis |
| Cluster-10108.11194 | 2.7475 | 4.5769 | 1.8494 | protein phosphorylation//galactose metabolic process//starch metabolic process//sucrose metabolic process//cartilage development |
| Cluster-10108.10650 | 6.2943 | 7.2261 |  | carbohydrate metabolic process//starch metabolic process//sucrose metabolic process |
| Cluster-10108.9751 | 1.1766 | 1.8018 |  | carbohydrate metabolic process//mannose metabolic process |
| Cluster-10108.9728 | 1.2993 | 2.2251 |  | carbohydrate metabolic process//lipid glycosylation |
| Cluster-10108.23066 | 1.3466 | 2.9644 |  | carbohydrate metabolic process//sucrose metabolic process//regulation of transcription, DNA-templated//starch metabolic process |
| Cluster-10108.13838 | 2.6529 | 2.8219 |  | lipid glycosylation//carbohydrate metabolic process |
| Cluster-10108.16599 | 1.2837 | 2.8125 | 1.5468 | -- |
| Cluster-10108.6091 | 1.3712 | 1.4538 |  | protein phosphorylation |
| Cluster-10108.8701 | 1.8517 | 4.8945 | 3.0659 | glycerolipid metabolic process//galactose metabolic process//glycosphingolipid metabolic process//carbohydrate metabolic process//glycosaminoglycan catabolic process |
| Cluster-10108.17412 | 3.4957 | 4.8386 |  | -- |
| Cluster-10108.13765 | 3.2004 | 5.2639 |  | actin cytoskeleton organization//sucrose metabolic process//starch metabolic process//cellulose biosynthetic process//UDP-glucose metabolic process |
| Cluster-10108.5058 | 1.7763 | 2.6326 |  | photosynthesis, light reaction//obsolete electron transport |
| Cluster-10108.15526 | 1.824 | 2.3978 |  | phosphoenolpyruvate-dependent sugar phosphotransferase system//carbohydrate transport |
| Cluster-10108.12928 | 1.0747 | 1.8175 |  | globoside metabolic process//carbohydrate metabolic process//glycolipid biosynthetic process//cell wall biogenesis |
| Cluster-10108.6144 | 1.1543 | 1.6236 |  | -- |
| Cluster-10108.14466 | 1.2019 | 1.9494 |  | starch metabolic process//photosystem II assembly//sucrose metabolic process//(1->3)-beta-D-glucan biosynthetic process |
| Cluster-10108.19077 | 1.0499 | 1.7102 |  | starch metabolic process//nucleotide metabolic process//sucrose metabolic process//amine metabolic process//oxidation-reduction process//carbohydrate metabolic process |
| Cluster-10108.17259 | 1.6495 | 2.5263 |  | starch metabolic process//sucrose biosynthetic process//sucrose metabolic process//pathogenesis//base-excision repair |
| Cluster-10108.13693 | 1.4914 | 2.8483 | 1.3767 | starch metabolic process//sucrose metabolic process//cell wall modification |
| Cluster-10108.3987 | 4.0663 | 6.0826 |  | chlorophyll catabolic process//chlorophyll metabolic process |
| Cluster-10108.3376 | 2.8879 | 2.5469 |  | chlorophyll catabolic process//chlorophyll metabolic process |
| Cluster-10108.5701 | 1.4262 | 2.0959 |  | regulation of transcription by RNA polymerase II//carbohydrate metabolic process//nitrogen compound metabolic process//proteolysis |
| Cluster-10108.12299 | 1.3016 | 2.5548 | 1.2724 | viral genome replication//fructose metabolic process//purine nucleobase metabolic process//mannose metabolic process//gluconeogenesis//glycolytic process//galactose metabolic process//transcription, DNA-templated//pentose-phosphate shunt |
| Cluster-10108.8360 | 4.2827 | 7.4092 |  | sucrose metabolic process//starch metabolic process//UDP-glucose metabolic process//cellulose biosynthetic process |
| Cluster-10108.5648 | 1.5884 | 2.8774 |  | sucrose metabolic process//(1->3)-beta-D-glucan biosynthetic process//transmembrane transport//starch metabolic process |
| Cluster-10108.12901 | 1.2363 | 3.6084 | 2.3912 | sucrose metabolic process//starch metabolic process//carbohydrate metabolic process |
| Cluster-10108.12547 | 2.3369 | 2.2804 |  | energy reserve metabolic process//vacuolar proton-transporting V-type ATPase complex assembly |
| Cluster-10108.11011 | 1.5884 | 2.9082 | 1.3397 | carbon utilization//glycolytic process//gluconeogenesis//purine nucleobase metabolic process |
| Cluster-10108.4065 | 2.3378 | 4.5346 | 2.2152 | UDP-glucose metabolic process//cellulose biosynthetic process//sucrose metabolic process//starch metabolic process |
| Cluster-10108.18478 | 2.5119 | 5.4287 | 2.9375 | viral life cycle//sucrose metabolic process//obsolete acyl-carrier-protein biosynthetic process//cellulose biosynthetic process//UDP-glucose metabolic process//starch metabolic process |
| Cluster-10108.8777 | 2.1416 | 3.5081 |  | -- |
| Cluster-10108.20770 | 1.9164 | 3.161 |  | bone remodeling//nuclear-transcribed mRNA catabolic process, nonsense-mediated decay//ATP metabolic process//purine nucleobase metabolic process |
| Cluster-10108.20048 | 1.1835 | 2.0475 |  | chlorophyll biosynthetic process//chlorophyll metabolic process |
| Cluster-10108.13153 | -2.5014 | -3.8176 | -1.2984 | regulation of transcription, DNA-templated//obsolete electron transport//photosynthesis, light reaction//methionine metabolic process//carbohydrate metabolic process |
| Cluster-10108.13268 | -1.2498 | -2.023 |  | regulation of transcription, DNA-templated//phosphorelay signal transduction system//regulation of carbohydrate metabolic process//phosphorylation |
| Cluster-10108.16383 | -1.0254 | -1.96 |  | proteolysis//carbohydrate metabolic process//nitrogen compound metabolic process//regulation of catalytic activity |
| Cluster-10108.11107 | -1.0168 | -1.1838 |  | carbohydrate metabolic process |
| Cluster-10108.15217 | -2.6158 | -3.0221 |  | carbohydrate metabolic process |
| Cluster-10108.18294 | -1.797 | -2.8168 | -1.0017 | carbohydrate transport//phosphoenolpyruvate-dependent sugar phosphotransferase system//oxidation-reduction process |
| Cluster-10108.10352 | -1.6265 | -2.3725 |  | carbohydrate metabolic process |
| Cluster-10108.11872 | -2.3925 | -3.1379 |  | carbohydrate metabolic process//cellular glucan metabolic process |
| Cluster-10108.14450 | -2.0443 | -2.6396 |  | carbohydrate metabolic process//sodium ion export across plasma membrane//sodium ion transport//pyruvate metabolic process//arginine metabolic process//intracellular protein transport//vesicle-mediated transport//regulation of RNA metabolic process//proline metabolic process |
| Cluster-10108.14240 | -1.5529 | -1.5812 |  | carbohydrate metabolic process |
| Cluster-10108.8488 | -1.7054 | -2.0888 |  | carbohydrate metabolic process//G protein-coupled receptor signaling pathway//DNA repair//tRNA threonylcarbamoyladenosine modification//DNA recombination//SRP-dependent cotranslational protein targeting to membrane//defense response to Gram-positive bacterium |
| Cluster-10108.15778 | -2.0057 | -1.3437 |  | carbohydrate metabolic process//sucrose metabolic process//starch metabolic process |
| Cluster-10108.2272 | -3.3658 | -4.4559 |  | carbohydrate metabolic process//mannose metabolic process |
| Cluster-10108.9703 | -1.2526 | -1.1037 |  | carbohydrate metabolic process//nucleotide metabolic process//L-arabinose metabolic process//regulation of transcription, DNA-templated |
| Cluster-10108.1821 | -4.9498 | -5.7223 |  | carbohydrate metabolic process |
| Cluster-10108.4680 | -1.9822 | -1.1149 |  | carbohydrate metabolic process//lipid glycosylation//apoptotic process |
| Cluster-10108.17051 | -1.1783 |  | 1.7005 | carbohydrate metabolic process |
| Cluster-10108.11952 | -1.8271 | -1.5679 |  | carbohydrate metabolic process |
| Cluster-10108.13123 | -2.3068 | -3.4396 | -1.116 | carbohydrate metabolic process//DNA-templated transcription, initiation//regulation of transcription, DNA-templated |
| Cluster-10108.14665 | -1.5033 |  | 1.3371 | carbohydrate metabolic process//mannose metabolic process |
| Cluster-10108.14666 | -1.608 | -2.4513 |  | carbohydrate metabolic process//sucrose metabolic process//starch metabolic process |
| Cluster-10108.18090 | -1.6054 | -2.6666 | -1.0444 | carbohydrate metabolic process |
| Cluster-10108.17474 | -2.5514 | -3.4429 |  | carbohydrate metabolic process//GPI anchor biosynthetic process |
| Cluster-10108.11671 | -1.2437 |  |  | carbohydrate metabolic process//organic substance metabolic process |
| Cluster-10108.10161 | -1.1168 | -1.3012 |  | carbohydrate metabolic process |
| Cluster-10108.2392 | -3.3662 | -3.1594 |  | carbohydrate metabolic process//glyoxylate cycle//carboxylic acid metabolic process//capsule polysaccharide biosynthetic process |
| Cluster-10108.19878 | -2.4728 | -3.2907 |  | carbohydrate metabolic process |
| Cluster-10108.18966 | -1.5779 | -2.1005 |  | carbohydrate metabolic process//oxidation-reduction process//glycerol-3-phosphate catabolic process//glycerolipid metabolic process//glycerol-3-phosphate metabolic process//mitochondrial electron transport, cytochrome c to oxygen |
| Cluster-10108.19510 | -1.382 | -2.4981 | -1.0986 | carbohydrate metabolic process//mitigation of host immune response by virus |
| Cluster-10108.1764 | -6.9688 | -8.4827 |  | carbohydrate metabolic process |
| Cluster-10108.27305 | -8.0402 | -9.6465 |  | carbohydrate metabolic process//polysaccharide catabolic process |
| Cluster-10108.2209 | -5.0282 | -5.7409 |  | carbohydrate metabolic process |
| Cluster-10108.4745 | -4.8889 | -5.2543 |  | carbohydrate metabolic process |
| Cluster-10108.6975 | -1.3928 | -2.143 |  | carbohydrate metabolic process//protein trimerization//regulation of transcription, DNA-templated//protein import into nucleus//protein tetramerization |
| Cluster-10108.11436 | -2.2569 | -2.6445 |  | carbohydrate metabolic process |
| Cluster-10108.2874 | -2.659 |  |  | carbohydrate metabolic process |
| Cluster-10108.4367 | -2.6055 |  |  | carbohydrate metabolic process |
| Cluster-10108.13482 | -1.0552 | -1.59 |  | carbohydrate metabolic process//heme biosynthetic process//polysaccharide catabolic process//chlorophyll metabolic process//signal transduction//tetrapyrrole biosynthetic process//protein phosphorylation//guanosine tetraphosphate metabolic process |
| Cluster-10108.3152 | -4.4401 | -3.4904 |  | carbohydrate metabolic process |
| Cluster-10108.2666 | -1.4663 | -1.7785 |  | carbohydrate metabolic process |
| Cluster-10108.17008 | -1.104 | -2.102 |  | carbohydrate metabolic process |
| Cluster-10108.7991 | -1.4322 | -1.6585 |  | carbohydrate transport//phosphoenolpyruvate-dependent sugar phosphotransferase system |
| Cluster-10108.2607 | -2.6731 | -3.3749 |  | carbohydrate metabolic process |
| Cluster-10108.20620 | -1.157 | -1.7514 |  | carbohydrate metabolic process |
| Cluster-10108.4688 | -2.4469 | -3.5241 |  | carbohydrate metabolic process//polysaccharide catabolic process |
| Cluster-10108.2415 | -8.3601 |  |  | carbohydrate metabolic process |
| Cluster-10108.2177 | -4.5037 | -7.1028 | -2.5823 | carbohydrate metabolic process |
| Cluster-10108.21852 | -1.2483 | -1.8597 |  | carbohydrate metabolic process |
| Cluster-10108.764 | -3.4535 | -4.0608 |  | carbohydrate metabolic process |
| Cluster-10108.7081 | -1.1468 | -1.341 |  | carbohydrate metabolic process |
| Cluster-10108.540 | -4.7105 | -5.4534 |  | carbohydrate metabolic process//polysaccharide catabolic process |
| Cluster-10108.25796 | -11.558 | -11.396 |  | carbohydrate metabolic process//cell division |
| Cluster-10108.19752 | -1.3851 | -2.3854 |  | carbohydrate metabolic process//positive regulation of viral transcription//proteolysis//polysaccharide catabolic process |
| Cluster-10108.2895 | -3.307 | -3.141 |  | carbohydrate metabolic process |
| Cluster-10108.19864 | -1.1278 |  |  | carbohydrate metabolic process//starch metabolic process//sucrose metabolic process |
| Cluster-10108.1706 | -3.7457 | -5.7555 |  | carbohydrate metabolic process//starch metabolic process//sucrose metabolic process |
| Cluster-10108.2525 | -3.1979 | -5.8951 | -2.6805 | carbohydrate metabolic process//translation//ribosome biogenesis//lipid glycosylation |
| Cluster-10108.17928 | -1.3131 | -2.3193 |  | carbohydrate metabolic process |
| Cluster-10108.13402 | -1.9027 | -1.9197 |  | photosynthesis |
| Cluster-10108.14281 | -1.5786 | -1.9191 |  | photosystem II assembly |
| Cluster-10108.13099 | -2.1398 | -2.7041 |  | photosynthesis//mRNA 3'-end processing//transcription, DNA-templated//protein phosphorylation |
| Cluster-10108.11420 | -1.6429 | -1.5963 |  | photosynthesis |
| Cluster-10108.12860 | -1.9348 | -1.914 |  | photosynthesis |
| Cluster-10108.15975 | -2.3849 | -2.6265 |  | photosynthesis//proline metabolic process//sodium ion export across plasma membrane//pathogenesis//regulation of DNA replication//regulation of transcription, DNA-templated//arginine metabolic process//secretion//pyruvate metabolic process//sodium ion transport |
| Cluster-10108.9040 | -2.3335 | -3.2984 |  | photosynthesis//oxidation-reduction process |
| Cluster-10108.15211 | -2.2277 | -2.1465 |  | photosynthesis |
| Cluster-10108.16359 | -2.1238 | -2.5744 |  | photosynthesis, light reaction//obsolete electron transport |
| Cluster-10108.7533 | -1.8417 | -1.1266 |  | photosynthetic electron transport chain |
| Cluster-10108.17303 | -2.7831 | -2.9839 |  | photosynthesis//inositol phosphoceramide metabolic process |
| Cluster-10108.16857 | -2.0098 | -2.2347 |  | photosynthesis//G protein-coupled receptor signaling pathway//DNA topological change//transcription by RNA polymerase III//mitochondrial fusion |
| Cluster-10108.13676 | -2.1284 | -2.2901 |  | photosystem II stabilization//photosystem II assembly |
| Cluster-10108.13608 | -1.931 | -2.0453 |  | photosynthesis//regulation of phosphoprotein phosphatase activity |
| Cluster-10108.15256 | -1.9897 | -1.9281 |  | photosynthesis |
| Cluster-10108.12132 | -1.7498 | -1.4408 |  | photosynthesis |
| Cluster-10108.13580 | -2.3139 | -2.8737 |  | photosynthesis//DNA replication |
| Cluster-10108.14312 | -1.8635 | -1.7581 |  | photosynthesis |
| Cluster-10108.10377 | -2.1069 |  |  | photosynthesis |
| Cluster-10108.15172 | -1.8851 | -1.9734 |  | photosynthesis |
| Cluster-10108.13669 | -1.8073 | -1.9382 |  | photosynthesis//protein tetramerization//DNA replication |
| Cluster-10108.17122 | -3.7197 | -4.857 |  | photosynthesis//starch metabolic process//sucrose metabolic process |
| Cluster-10108.9242 | -1.4668 | -2.0598 |  | photosynthesis |
| Cluster-10108.3991 | -2.1631 | -2.4547 |  | photosynthesis |
| Cluster-10108.13803 | -1.9655 | -1.9248 |  | photosynthesis |
| Cluster-10108.14061 | -1.5793 | -1.6369 |  | photosynthesis//glycosaminoglycan catabolic process//photosystem II assembly//Wnt signaling pathway//glycerolipid metabolic process//glycosphingolipid metabolic process//galactose metabolic process |
| Cluster-10108.12744 | -1.5827 | -2.3352 |  | photosynthesis//lipid metabolic process |
| Cluster-10108.2840 | -2.102 | -1.9331 |  | photosynthesis, light reaction//photosynthesis |
| Cluster-10108.8699 | -2.5043 | -3.158 |  | photosynthesis |
| Cluster-10108.11193 | -1.7398 | -1.2587 |  | photosystem II assembly |
| Cluster-10108.5459 | -1.8939 | -1.7319 |  | photosynthesis |
| Cluster-10108.14699 | -1.314 | -1.1934 |  | photosynthesis//photosystem II assembly |
| Cluster-10108.19251 | -3.1981 | -5.3122 | -2.0939 | photosynthesis//restriction-modification system evasion by virus |
| Cluster-10108.8816 | -1.1497 |  |  | photosynthesis |
| Cluster-10108.15601 | -1.254 | -2.4006 | -1.1281 | photosynthesis |
| Cluster-10108.6959 | -1.3598 |  |  | photosynthesis |
| Cluster-10108.4314 | -2.3716 | -3.6637 |  | photosynthesis |
| Cluster-10108.3750 | -1.2472 |  |  | photosynthesis |
| Cluster-10108.3062 | -3.4767 | -4.1026 |  | chlorophyll catabolic process//chlorophyll metabolic process//proteolysis |
| Cluster-10108.13895 | -1.3867 | -1.1912 |  | chlorophyll metabolic process//biosynthetic process |
| Cluster-10108.10938 | -1.0306 | -1.4601 |  | chlorophyll metabolic process//heme biosynthetic process//regulation of transcription, DNA-templated |
| Cluster-10108.13737 | -2.6704 | -3.0347 |  | pentose-phosphate shunt//UDP-glucose metabolic process//hemolysis by symbiont of host erythrocytes//carbon utilization//inositol metabolic process//glycolytic process//mannose metabolic process//gluconeogenesis//fructose metabolic process |
| Cluster-10108.13679 | -1.8965 | -2.0379 |  | pentose-phosphate shunt//carbon utilization//mannose metabolic process//gluconeogenesis//inositol metabolic process//glycolytic process//proteolysis//fructose metabolic process |
| Cluster-10108.1273 | -1.7802 | -2.6167 |  | isoprenoid biosynthetic process//photosynthesis |
| Cluster-10108.13826 | -1.4531 | -1.0649 |  | -- |
| Cluster-10108.12796 | -3.4642 | -4.7786 | -1.2978 | -- |
| Cluster-10108.6327 | -1.1604 | -1.7987 |  | gluconeogenesis//mannose metabolic process//glycolytic process//fructose metabolic process//NADP biosynthetic process//photosynthetic electron transport chain//nicotinamide metabolic process//pentose-phosphate shunt//nicotinate nucleotide metabolic process//galactose metabolic process |
| Cluster-10108.5016 | -3.8163 | -5.7757 | -1.943 | polysaccharide catabolic process//sucrose metabolic process//starch metabolic process |
| Cluster-10108.25901 | -8.2451 | -10.11 |  | polysaccharide catabolic process//carbohydrate metabolic process |
| Cluster-10108.15079 | -1.24 | -1.1369 |  | polysaccharide catabolic process//carbohydrate metabolic process |
| Cluster-10108.24601 | -1.9256 | -2.3285 |  | polysaccharide catabolic process//carbohydrate metabolic process |
| Cluster-5920.0 | -5.5268 | -6.3718 |  | polysaccharide catabolic process//carbohydrate metabolic process |
| Cluster-10108.18896 | -1.1367 | -1.3289 |  | nitrogen compound metabolic process//carbohydrate metabolic process |
| Cluster-10108.11425 | -3.9652 | -5.2726 | -1.2918 | -- |
| Cluster-10108.14036 | -2.1145 | -2.0905 |  | -- |
| Cluster-10108.13769 | -1.4237 | -1.9712 |  | lipid catabolic process//carbohydrate metabolic process//mannose metabolic process//digestion |
| Cluster-10108.18745 | -2.2514 | -2.7335 |  | fructose metabolic process//nucleoside metabolic process//mannose metabolic process//gluconeogenesis//glycolytic process//inositol metabolic process//carbon utilization//coenzyme A biosynthetic process//pantothenate biosynthetic process//pentose-phosphate shunt |
| Cluster-10108.24060 | -4.8823 | -6.5167 | -1.619 | fructose metabolic process//mannose metabolic process//G protein-coupled receptor signaling pathway |
| Cluster-10108.20913 | -1.9667 | -1.8588 |  | negative regulation of microtubule motor activity//lipid glycosylation//carbohydrate metabolic process |
| Cluster-10108.12688 | -2.373 | -3.273 |  | -- |
| Cluster-10108.8885 | -1.1969 | -1.8969 |  | -- |
| Cluster-10108.17796 | -1.2812 | -1.8911 |  | -- |
| Cluster-10108.3865 | -2.0037 | -2.8446 |  | response to nitrate//nitrate transport |
| Cluster-10108.12219 | -2.1631 | -2.1435 |  | -- |
| Cluster-10108.13184 | -1.6826 | -1.8775 |  | isoprenoid biosynthetic process |
| Cluster-10108.10245 | -1.1167 | -1.6341 |  | isoprenoid biosynthetic process |
| Cluster-10108.13118 | -2.0947 | -1.7701 |  | -- |
| Cluster-10108.8479 | -2.2612 | -3.5282 | -1.2501 | mitochondrion organization//mitochondrion inheritance |
| Cluster-10108.10058 | -2.1229 | -2.4288 |  | -- |
| Cluster-10108.10550 | -1.3117 | -1.648 |  | mitochondrial respiratory chain complex III assembly |
| Cluster-10108.18496 | -1.181 | -1.0669 |  | DNA methylation//DNA methylation on adenine//carbohydrate metabolic process |
| Cluster-10108.13969 | -1.4528 | -1.6999 |  | -- |
| Cluster-10108.12051 | -1.4036 | -1.7604 |  | biotin transport//starch metabolic process//sucrose metabolic process//carbohydrate metabolic process |
| Cluster-10108.7255 | -1.6129 | -1.4242 |  | -- |
| Cluster-10108.11274 | -1.662 | -1.5059 |  | pentose-phosphate shunt//glycolytic process//gluconeogenesis//sucrose metabolic process//starch metabolic process |
| Cluster-10108.17146 | -2.0573 | -2.7086 |  | -- |
| Cluster-10108.22747 | -4.1983 | -4.3834 |  | -- |
| Cluster-10108.15771 | -1.2723 | -1.6902 |  | chorion-containing eggshell formation |
| Cluster-10108.10659 | -2.5443 | -2.968 |  | regulation of protein secretion//carbohydrate metabolic process//cellular glucan metabolic process |
| Cluster-10108.7218 | -1.9315 | -1.2865 |  | -- |
| Cluster-10108.15175 | -1.3562 |  |  | -- |
| Cluster-10108.12355 | -1.4282 | -1.9575 |  | guanosine tetraphosphate metabolic process//photosynthesis |
| Cluster-10108.17162 | -1.5289 | -1.5548 |  | reductive tricarboxylic acid cycle//carbon fixation//gluconeogenesis//tricarboxylic acid cycle |
| Cluster-10108.12197 | -1.8751 | -2.0865 |  | carbon fixation//reductive tricarboxylic acid cycle//tricarboxylic acid cycle//gluconeogenesis |
| Cluster-10108.15603 | -1.7521 | -1.5484 |  | chemical synaptic transmission//proteasome assembly//photosynthesis//regulation of GTPase activity//ion transport//signal transduction |
| Cluster-10108.6487 | -1.1246 |  |  | chemical synaptic transmission//signal transduction//photosynthesis |
| Cluster-10108.10090 | -2.2515 | -2.0823 |  | -- |
| Cluster-10108.21189 | -2.2988 | -2.4948 |  | -- |
| Cluster-10108.9353 | -1.1735 | -1.1474 |  | -- |
| Cluster-10108.5875 | -2.5466 | -3.7653 |  | L-arabinose metabolic process//carbohydrate metabolic process//nucleotide metabolic process |
| Cluster-10108.13721 | -1.0298 |  | 1.084 | -- |
| Cluster-10108.6520 | -1.9547 | -2.8138 |  | vesicle-mediated transport |
| Cluster-10108.17645 | -1.08 | -1.7182 |  | regulation of transcription, DNA-templated//DNA replication initiation//intracellular protein transport//vesicle-mediated transport |
| Cluster-10108.25472 | -1.6862 | -1.764 |  | DNA integration |
| Cluster-10108.8674 | -1.1694 |  |  | cation transport |
| Cluster-10108.9007 | -1.5844 | -2.492 |  | tetrapyrrole biosynthetic process//NAD biosynthetic process//chlorophyll metabolic process |
| Cluster-10108.14453 | -1.1073 | -1.1476 |  | pentose-phosphate shunt//carbon utilization//pentose-phosphate shunt, non-oxidative branch |
| Cluster-10108.15015 | -1.2177 |  |  | -- |
| Cluster-10108.274 | -5.2699 | -4.5375 |  | -- |
| Cluster-10108.28159 |  | -6.4976 |  | mitochondrial electron transport, cytochrome c to oxygen |
| Cluster-315.0 |  | -6.6222 |  | mitochondrial electron transport, cytochrome c to oxygen |
| Cluster-10108.17829 |  | 1.5199 |  | sulfur compound metabolic process//Wnt signaling pathway//mitochondrial electron transport, cytochrome c to oxygen |
| Cluster-10108.18300 |  | 1.0625 |  | cell adhesion//mitochondrial electron transport, cytochrome c to oxygen//oxidation-reduction process |
| Cluster-10108.11159 |  | 1.931 | 1.2492 | mitochondrial electron transport, cytochrome c to oxygen |
| Cluster-645.0 |  | -6.4504 |  | mitochondrial electron transport, cytochrome c to oxygen |
| Cluster-10108.21659 |  | 1.8076 |  | mitochondrial electron transport, cytochrome c to oxygen//regulation of translation |
| Cluster-10108.1832 |  | -2.4466 |  | carbohydrate metabolic process//DNA-templated transcription, initiation//polysaccharide transport//polysaccharide catabolic process |
| Cluster-10108.721 |  | -4.878 |  | carbohydrate metabolic process//sucrose metabolic process//starch metabolic process |
| Cluster-10108.19482 |  | -2.9885 | -2.2678 | carbohydrate metabolic process |
| Cluster-10108.19050 |  | 1.6638 |  | carbohydrate metabolic process |
| Cluster-10108.10493 |  | 1.9302 |  | carbohydrate metabolic process |
| Cluster-10108.10832 |  | 1.4312 |  | carbohydrate metabolic process//intracellular signal transduction |
| Cluster-10108.19041 |  | -1.1926 |  | carbohydrate metabolic process//transcription, DNA-templated//protein phosphorylation |
| Cluster-10108.13784 |  | 1.9532 | 1.6595 | carbohydrate metabolic process//starch metabolic process//sucrose metabolic process//angiotensin-activated signaling pathway |
| Cluster-10108.10563 |  | -1.1819 |  | carbohydrate metabolic process//sucrose metabolic process//starch metabolic process |
| Cluster-10108.10873 |  | -1.0271 |  | carbohydrate metabolic process//globoside metabolic process//cell wall biogenesis//glycolipid biosynthetic process//regulation of RNA metabolic process//tRNA threonylcarbamoyladenosine modification |
| Cluster-10108.10735 |  | -1.1292 |  | carbohydrate metabolic process |
| Cluster-10108.11094 |  | 1.3912 |  | carbohydrate metabolic process |
| Cluster-10108.8091 |  | -1.1123 |  | carbohydrate metabolic process//regulation of transcription, DNA-templated |
| Cluster-10108.17269 |  | -1.0454 |  | carbohydrate metabolic process |
| Cluster-10108.5283 |  | -3.5325 |  | carbohydrate metabolic process//defense response to bacterium |
| Cluster-10108.21506 |  | 4.4694 | 3.2684 | carbohydrate metabolic process |
| Cluster-10108.13057 |  | 1.688 | 1.2818 | carbohydrate metabolic process//(1->3)-beta-D-glucan biosynthetic process//sucrose metabolic process//starch metabolic process |
| Cluster-10108.3469 |  | 2.9268 |  | carbohydrate metabolic process |
| Cluster-10108.22723 |  | 4.1987 |  | carbohydrate metabolic process |
| Cluster-10108.5715 |  | -1.0449 |  | carbohydrate metabolic process |
| Cluster-10108.5303 |  | -1.8228 |  | carbohydrate metabolic process |
| Cluster-10108.14328 |  | -1.2398 |  | carbohydrate metabolic process//polysaccharide catabolic process//polysaccharide transport |
| Cluster-10108.7114 |  | 1.0871 |  | carbohydrate metabolic process//starch metabolic process//sucrose metabolic process |
| Cluster-10108.19710 |  | 1.9542 | 1.713 | carbohydrate metabolic process |
| Cluster-10108.6736 |  | 1.4184 |  | carbohydrate metabolic process |
| Cluster-10108.3564 |  | 9.2438 |  | carbohydrate metabolic process |
| Cluster-10108.4471 |  | 3.2024 |  | carbohydrate metabolic process |
| Cluster-10108.24185 |  | 3.364 |  | carbohydrate metabolic process |
| Cluster-10108.4552 |  | 1.0379 |  | carbohydrate metabolic process |
| Cluster-10108.12931 |  | 1.9097 |  | carbohydrate metabolic process |
| Cluster-10108.6351 |  | 2.2385 |  | carbohydrate metabolic process//polysaccharide catabolic process//DNA-templated transcription, initiation |
| Cluster-10108.9307 |  | 1.543 | 1.3043 | carbohydrate metabolic process |
| Cluster-10108.20371 |  | -1.1718 |  | carbohydrate metabolic process//transmembrane transport//sodium ion export across plasma membrane//arginine metabolic process//polysaccharide catabolic process//sodium ion transport//pyruvate metabolic process//proline metabolic process |
| Cluster-10108.20026 |  | 1.4078 |  | carbohydrate metabolic process |
| Cluster-10108.19532 |  | -1.3649 |  | carbohydrate metabolic process |
| Cluster-10108.5642 |  | 1.7041 |  | carbohydrate metabolic process//sucrose metabolic process//starch metabolic process//regulation of transcription, DNA-templated//organic phosphonate catabolic process |
| Cluster-10108.10573 |  | 2.2581 | 1.8647 | carbohydrate metabolic process |
| Cluster-10108.15792 |  | -1.1114 |  | carbohydrate metabolic process |
| Cluster-10108.13309 |  | -1.8594 | -1.0487 | carbohydrate metabolic process |
| Cluster-10108.22048 |  | 3.5428 |  | carbohydrate metabolic process |
| Cluster-10108.20809 |  | 1.7646 | 1.0207 | carbohydrate metabolic process//cellulose microfibril organization |
| Cluster-10108.13589 |  | -1.4183 |  | carbohydrate metabolic process//glycosphingolipid metabolic process |
| Cluster-10108.5017 |  | 2.478 |  | carbohydrate metabolic process |
| Cluster-10108.5383 |  | -1.4854 | -1.3843 | carbohydrate metabolic process//protein complex oligomerization |
| Cluster-10108.4728 |  | 2.3811 |  | carbohydrate metabolic process//multivesicular body sorting pathway//regulation of translation |
| Cluster-10108.16408 |  | 1.5583 | 1.375 | carbohydrate metabolic process |
| Cluster-10108.11277 |  | -1.0618 |  | carbohydrate metabolic process//transmembrane transport |
| Cluster-10108.14673 |  | 2.358 | 1.4538 | carbohydrate metabolic process |
| Cluster-10108.16838 |  | 1.134 |  | carbohydrate metabolic process//tRNA aminoacylation for protein translation//tRNA aminoacylation |
| Cluster-10108.4060 |  | 3.1207 | 2.1705 | carbohydrate metabolic process |
| Cluster-10108.10186 |  | 2.3809 |  | carbohydrate metabolic process |
| Cluster-10108.29509 |  | -1.0284 |  | carbohydrate metabolic process |
| Cluster-10108.3019 |  | 2.5365 |  | carbohydrate metabolic process |
| Cluster-10108.23082 |  | 5.6369 |  | carbohydrate metabolic process |
| Cluster-10108.3465 |  | 4.1639 |  | carbohydrate metabolic process |
| Cluster-10108.17693 |  | 1.2299 | 1.5342 | sodium ion export across plasma membrane//arginine metabolic process//sodium ion transport//pyruvate metabolic process//protein phosphorylation//proline metabolic process |
| Cluster-10108.14187 |  | 1.7805 |  | fructose metabolic process//sodium ion export across plasma membrane//glycolytic process//gluconeogenesis//pyruvate metabolic process//sodium ion transport//arginine metabolic process//mannose metabolic process//proline metabolic process//galactose metabolic process//pentose-phosphate shunt |
| Cluster-10108.22950 |  | -2.1394 |  | obsolete electron transport//photosynthetic electron transport in photosystem II//proline metabolic process//photosynthesis, light reaction//transcription by RNA polymerase I//sodium ion export across plasma membrane//photosynthetic electron transport chain//arginine metabolic process//ATP-dependent chromatin remodeling//sodium ion transport//pyruvate metabolic process |
| Cluster-10108.4162 |  | -1.0147 |  | sodium ion export across plasma membrane//regulation of transcription, DNA-templated//proline metabolic process//arginine metabolic process//pyruvate metabolic process//sodium ion transport |
| Cluster-10108.20721 |  | 1.8956 | 2.0714 | sodium ion export across plasma membrane//pyruvate metabolic process//sodium ion transport//arginine metabolic process//protein phosphorylation//proline metabolic process |
| Cluster-10628.0 |  | -5.2476 |  | protein phosphorylation//phosphorylation//serine family amino acid metabolic process |
| Cluster-10108.10958 |  | 2.0356 | 1.4185 | electron transport chain//protein phosphorylation |
| Cluster-10108.29468 |  | -3.6032 |  | recognition of pollen//protein phosphorylation |
| Cluster-10108.13227 |  | -1.491 |  | L-phenylalanine biosynthetic process//glutamate metabolic process//aromatic amino acid family catabolic process//proteolysis//tryptophan biosynthetic process//alkaloid biosynthetic process//2-oxoglutarate metabolic process//tyrosine biosynthetic process |
| Cluster-10108.25645 |  | -3.9227 |  | -- |
| Cluster-10108.13556 |  | -1.3823 |  | protein phosphorylation |
| Cluster-10108.11281 |  | 1.3648 |  | DNA repair//Golgi organization//proteolysis//DNA recombination//cell motility |
| Cluster-10108.21091 |  | 2.2141 |  | -- |
| Cluster-10108.5605 |  | -1.4123 |  | recognition of pollen//protein phosphorylation |
| Cluster-10108.11489 |  | 1.407 |  | protein phosphorylation |
| Cluster-10108.16692 |  | 1.9718 | 1.1576 | -- |
| Cluster-10108.8930 |  | -1.5519 |  | -- |
| Cluster-10108.15009 |  | 2.2972 | 1.38 | -- |
| Cluster-10108.14869 |  | -1.4048 |  | transport//transmembrane transport//neuropeptide signaling pathway//regulation of vasoconstriction |
| Cluster-10108.157 |  | 5.3775 |  | metal ion transport |
| Cluster-10108.15523 |  | 1.9899 |  | purine nucleobase biosynthetic process//purine nucleobase metabolic process |
| Cluster-10108.20586 |  | -2.1252 |  | protein phosphorylation |
| Cluster-10108.8807 |  | 1.1684 |  | protein phosphorylation//serine family amino acid metabolic process//phosphorylation |
| Cluster-10108.14508 |  | -1.1189 |  | recognition of pollen//protein phosphorylation//serine family amino acid metabolic process//phosphorylation |
| Cluster-10108.7286 |  | 1.3475 |  | -- |
| Cluster-10108.13508 |  | -1.0474 |  | chromatin remodeling |
| Cluster-10108.19874 |  | 2.1405 | 1.9344 | protein phosphorylation |
| Cluster-10108.10926 |  |  | 1.3221 | protein phosphorylation//C4-dicarboxylate transport |
| Cluster-10108.4865 |  | -1.6415 |  | oxidation-reduction process//protein phosphorylation |
| Cluster-10108.1917 |  | -6.459 |  | ATP metabolic process//viral DNA genome replication//proton transmembrane transport//modulation by virus of host cellular process |
| Cluster-10108.19149 |  | -1.5258 |  | chromatin remodeling |
| Cluster-10108.16095 |  | 3.6932 | 2.499 | -- |
| Cluster-10108.21252 |  | -2.1855 |  | protein phosphorylation//phosphorylation//serine family amino acid metabolic process |
| Cluster-10108.14009 |  | 1.0048 |  | protein phosphorylation |
| Cluster-10108.23966 |  | -2.7139 |  | regulation of transcription by RNA polymerase II |
| Cluster-10108.5226 |  |  | 1.5084 | protein phosphorylation//regulation of transcription, DNA-templated |
| Cluster-10108.16087 |  | -1.0658 |  | pathogenesis//protein phosphorylation |
| Cluster-10108.19628 |  | 3.3855 | 2.925 | chromatin remodeling |
| Cluster-10108.8363 |  | 1.6157 | 1.1063 | protein phosphorylation//mycotoxin biosynthetic process//nucleosome assembly |
| Cluster-10108.28377 |  | -1.4627 |  | protein phosphorylation//recognition of pollen//serine family amino acid metabolic process//phosphorylation |
| Cluster-10108.29709 |  | -2.9818 |  | protein phosphorylation |
| Cluster-10108.1930 |  | -2.954 |  | protein phosphorylation |
| Cluster-10108.8772 |  | 1.0407 |  | biosynthetic process |
| Cluster-8247.0 |  | -2.7311 |  | protein phosphorylation |
| Cluster-10108.7832 |  | 1.2618 |  | cellular biogenic amine metabolic process |
| Cluster-10108.17707 |  | 1.539 |  | protein phosphorylation |
| Cluster-10108.16798 |  | -1.1314 |  | protein phosphorylation//signal transduction |
| Cluster-10108.13962 |  | -1.1856 |  | protein phosphorylation//copper ion transport//copper ion transmembrane transport |
| Cluster-10108.27093 |  | -2.3839 |  | chromosome organization//metal ion transport |
| Cluster-10108.1916 |  | -6.0534 |  | protein folding |
| Cluster-10108.9194 |  | -1.5572 |  | protein phosphorylation |
| Cluster-10108.20670 |  | 2.0115 |  | protein phosphorylation |
| Cluster-10108.11251 |  | -1.6467 | -1.2425 | regulation of transcription, DNA-templated |
| Cluster-10108.12953 |  | 1.5105 |  | protein phosphorylation |
| Cluster-10108.1075 |  | -6.095 |  | ATP metabolic process//proton transmembrane transport |
| Cluster-10108.5868 |  | 2.3598 |  | proton transmembrane transport//oxidative phosphorylation |
| Cluster-10108.2633 |  | 1.5679 |  | DNA replication//signal transduction//DNA repair//DNA recombination//cell adhesion |
| Cluster-10108.21878 |  | -1.1991 |  | protein phosphorylation |
| Cluster-10108.18958 |  | -1.3925 |  | protein phosphorylation//cell population proliferation//signal transduction//growth |
| Cluster-10108.3652 |  | -2.6224 |  | protein phosphorylation//B cell receptor signaling pathway//T cell receptor signaling pathway |
| Cluster-10108.4137 |  | -2.1351 |  | protein phosphorylation//transport//pathogenesis |
| Cluster-10108.2748 |  | 2.3181 |  | protein phosphorylation |
| Cluster-10108.17117 |  | 1.631 |  | G protein-coupled receptor signaling pathway |
| Cluster-10108.4908 |  | -4.5409 |  | chromosome organization |
| Cluster-10108.9826 |  | -2.334 |  | protein phosphorylation |
| Cluster-10108.8977 |  | -1.0431 |  | protein phosphorylation |
| Cluster-10108.8574 |  | 1.0126 |  | arginine metabolic process//DNA replication//tRNA threonylcarbamoyladenosine modification//DNA repair//DNA recombination |
| Cluster-10108.17646 |  | -1.1478 |  | pantothenate biosynthetic process//coenzyme A biosynthetic process |
| Cluster-10108.5054 |  | 1.0545 |  | protein phosphorylation |
| Cluster-10108.16372 |  | 1.7083 |  | protein phosphorylation |
| Cluster-6828.0 |  | -3.7926 | -3.4851 | protein phosphorylation |
| Cluster-10108.14266 |  | -1.4952 |  | protein phosphorylation |
| Cluster-10108.9023 |  | 1.9617 | 1.3081 | protein phosphorylation |
| Cluster-10108.29517 |  | -2.4345 |  | protein phosphorylation |
| Cluster-10108.2153 |  | -6.2405 |  | fructose metabolic process//mannose metabolic process |
| Cluster-10108.17970 |  | -1.316 |  | protein phosphorylation |
| Cluster-10108.8067 |  | -1.0755 |  | L-phenylalanine biosynthetic process//phenylalanyl-tRNA aminoacylation//cellular biogenic amine metabolic process//protein transport//tryptophan biosynthetic process//tyrosine biosynthetic process//purine nucleobase metabolic process//ATP metabolic process |
| Cluster-10108.20805 |  | -1.8206 |  | protein phosphorylation//phosphorylation//serine family amino acid metabolic process |
| Cluster-6058.0 |  | -5.3626 |  | response to auxin//IMP biosynthetic process |
| Cluster-10108.6116 |  | 1.6304 | 1.743 | asparagine biosynthetic process//protein phosphorylation//alanine metabolic process//aspartate metabolic process |
| Cluster-10108.3426 |  | -1.4254 |  | protein phosphorylation |
| Cluster-10108.12869 |  | 1.7782 |  | -- |
| Cluster-10108.4335 |  | 3.3804 | 2.5098 | protein phosphorylation |
| Cluster-10108.10887 |  | 1.4675 |  | protein phosphorylation |
| Cluster-10108.13014 |  | 1.7182 | 1.1482 | signal transduction |
| Cluster-10108.12598 |  | 1.3661 | 1.0084 | transcription, DNA-templated//transport//transmembrane transport |
| Cluster-10108.17046 |  | -1.8445 |  | transport//protein phosphorylation//retrograde vesicle-mediated transport, Golgi to endoplasmic reticulum |
| Cluster-10108.6631 |  | 1.323 |  | ATP synthesis coupled proton transport//proton transmembrane transport//ATP metabolic process |
| Cluster-10108.9142 |  | -1.265 |  | starch metabolic process//galactose metabolic process//streptomycin biosynthetic process//protein phosphorylation//glucose 6-phosphate metabolic process//phosphatidylinositol metabolic process//sucrose metabolic process//gluconeogenesis//glycolytic process |
| Cluster-10108.21661 |  | -1.2934 |  | protein phosphorylation |
| Cluster-10108.18293 |  | -2.4075 |  | recognition of pollen//protein phosphorylation |
| Cluster-10108.18316 |  | 3.1677 | 2.3558 | protein phosphorylation |
| Cluster-10108.15983 |  | -1.0487 |  | protein phosphorylation//sodium ion transport//ubiquinone biosynthetic process//mitochondrial electron transport, NADH to ubiquinone |
| Cluster-10108.1712 |  | -3.7789 |  | glycyl-tRNA aminoacylation//L-serine metabolic process//glycine metabolic process//threonine metabolic process |
| Cluster-10108.16876 |  | 1.4317 |  | protein phosphorylation |
| Cluster-10108.19749 |  | 1.056 |  | protein phosphorylation |
| Cluster-10108.20807 |  | -2.916 |  | protein phosphorylation//phosphorylation//serine family amino acid metabolic process |
| Cluster-10108.18278 |  | -1.113 |  | DNA packaging//protein phosphorylation//regulation of autophagy |
| Cluster-10108.4992 |  | -1.9164 |  | protein phosphorylation |
| Cluster-10108.10759 |  | 1.4266 |  | -- |
| Cluster-10108.10033 |  | 2.6693 | 2.0599 | protein phosphorylation |
| Cluster-10108.3694 |  | -1.1313 |  | protein phosphorylation |
| Cluster-10108.6007 |  | 2.2059 | 1.8671 | chromatin remodeling |
| Cluster-10108.1894 |  | -6.5298 |  | -- |
| Cluster-10108.12985 |  | 1.1488 |  | protein phosphorylation//rRNA processing |
| Cluster-10108.22147 |  | -1.0611 |  | -- |
| Cluster-10108.15906 |  | 1.9834 |  | protein phosphorylation |
| Cluster-796.0 |  | -6.6222 |  | transport//transmembrane transport |
| Cluster-10108.19843 |  | -1.0624 |  | pyrimidine nucleobase metabolic process//microtubule anchoring//nucleobase-containing compound metabolic process |
| Cluster-10108.18050 |  | -1.9483 |  | protein phosphorylation |
| Cluster-10108.5932 |  | -1.1944 |  | protein phosphorylation//recognition of pollen |
| Cluster-10108.11661 |  | -1.2461 |  | protein phosphorylation |
| Cluster-10108.11959 |  | 2.1799 | 1.4823 | protein phosphorylation//cartilage development |
| Cluster-10108.21086 |  | -1.3995 |  | protein phosphorylation//recognition of pollen//cell adhesion |
| Cluster-10108.26189 |  | -5.3845 |  | protein phosphorylation//organic substance metabolic process |
| Cluster-10108.20890 |  | 1.1227 |  | protein phosphorylation |
| Cluster-8789.2 |  | 3.8547 |  | telomere maintenance//DNA repair//signal transduction//protein folding |
| Cluster-10108.10211 |  | 3.0652 | 1.9191 | D-alanine metabolic process//peptidoglycan biosynthetic process |
| Cluster-10108.25974 |  | -2.9529 |  | TMP biosynthetic process//pyrimidine nucleobase metabolic process |
| Cluster-6526.0 |  | -2.9556 |  | protein phosphorylation |
| Cluster-10108.25305 |  | -3.2864 |  | protein phosphorylation//phosphorylation//serine family amino acid metabolic process |
| Cluster-10108.15224 |  | -1.2808 |  | protein phosphorylation//chromatin remodeling//regulation of transcription, DNA-templated//histone exchange |
| Cluster-10108.18573 |  | -1.4419 | -1.0163 | protein phosphorylation//serine family amino acid metabolic process//phosphorylation |
| Cluster-10108.22295 |  | 3.5116 |  | protein phosphorylation |
| Cluster-10108.16232 |  | 1.2127 |  | proteolysis//virion attachment to host cell//phytochelatin biosynthetic process//response to metal ion |
| Cluster-10108.23192 |  | 2.3735 |  | protein phosphorylation |
| Cluster-10108.14260 |  | -1.9119 | -1.1663 | mating type determination//sodium ion transport//protein import//positive regulation of mating-type specific transcription, DNA-templated |
| Cluster-10108.18435 |  | 1.1742 |  | protein phosphorylation//transmembrane receptor protein serine/threonine kinase signaling pathway//ubiquinone biosynthetic process//serine family amino acid metabolic process//phosphorylation |
| Cluster-10108.20503 |  | -3.5998 |  | protein phosphorylation |
| Cluster-10108.19316 |  | -1.0561 |  | -- |
| Cluster-10108.6494 |  | 1.9375 |  | DNA replication termination |
| Cluster-10108.17428 |  | 1.4976 |  | DNA repair |
| Cluster-10108.6069 |  | -1.9165 |  | -- |
| Cluster-10108.10109 |  | 1.2288 |  | oxidation-reduction process |
| Cluster-10108.24987 |  | -1.8701 |  | protein phosphorylation |
| Cluster-10108.15564 |  | 1.5092 | 1.0074 | regulation of transcription, DNA-templated |
| Cluster-10108.21499 |  | 2.0558 | 1.4872 | -- |
| Cluster-10108.3550 |  | 4.1027 |  | recognition of pollen//protein phosphorylation//fumarate metabolic process |
| Cluster-10108.13207 |  | 1.599 |  | -- |
| Cluster-10108.11947 |  | -1.239 |  | proteolysis |
| Cluster-10108.13624 |  | 2.9065 | 2.1036 | galactose metabolic process//starch metabolic process//protein phosphorylation//sucrose metabolic process//transmembrane transport |
| Cluster-10108.11141 |  | -1.2282 |  | protein phosphorylation//ATP synthesis coupled proton transport//pathogenesis |
| Cluster-10713.0 |  | -5.1381 |  | protein phosphorylation |
| Cluster-10108.3900 |  |  | -1.058 | viral genome packaging//protein phosphorylation |
| Cluster-10108.10723 |  |  | -1.1019 | protein phosphorylation |
| Cluster-10108.175 |  | -4.1345 |  | protein phosphorylation |
| Cluster-10108.26677 |  | -2.5508 |  | protein phosphorylation |
| Cluster-10108.18681 |  |  | 1.0135 | protein phosphorylation//DNA recombination |
| Cluster-10108.12574 |  | 1.4168 |  | regulation of protein catabolic process//nitrogen fixation |
| Cluster-10108.1858 |  | -5.7148 |  | -- |
| Cluster-10108.7400 | 2.352 | 2.4808 |  | DNA repair//DNA replication |
| Cluster-10108.17449 | 2.1679 | 3.8447 |  | protein phosphorylation//regulation of transcription, DNA-templated |
| Cluster-10108.11269 | 1.0749 | 1.2125 |  | DNA replication//tRNA wobble uridine modification//Mo-molybdopterin cofactor biosynthetic process//DNA recombination//DNA repair |
| Cluster-10108.11379 | 3.2401 | 2.8321 |  | regulation of transcription, DNA-templated |
| Cluster-10108.15393 | 3.0091 | 2.3849 |  | -- |
| Cluster-10108.11941 | 2.4959 | 4.6337 | 2.1569 | regulation of transcription, DNA-templated |
| Cluster-10108.9587 | 1.5176 | 2.0718 |  | -- |
| Cluster-10108.13250 | 1.5434 | 2.1248 |  | tRNA wobble uridine modification |
| Cluster-10108.15977 | 1.8252 | 2.0385 |  | -- |
| Cluster-10108.18459 | 3.6393 | 4.1004 |  | -- |
| Cluster-10108.13058 | 1.4877 | 1.9936 |  | positive regulation of canonical Wnt signaling pathway//negative regulation of transforming growth factor beta receptor signaling pathway |
| Cluster-10108.10017 | 1.6245 | 2.8517 |  | regulation of transcription, DNA-templated |
| Cluster-10108.8755 | 2.27 | 2.4523 |  | defense response to other organism//nucleobase-containing compound metabolic process |
| Cluster-10108.9995 | 1.9645 | 1.9991 |  | spindle assembly//microtubule organizing center organization//histone deacetylation//Golgi organization |
| Cluster-10108.14393 | 3.7868 | 6.2128 | 2.4458 | protein phosphorylation |
| Cluster-10108.22041 | 2.8894 | 3.0808 |  | protein phosphorylation |
| Cluster-10108.5253 | 1.5436 |  |  | protein phosphorylation |
| Cluster-10108.20447 | 1.0636 | 1.8996 |  | protein phosphorylation |
| Cluster-10108.7240 | 3.7428 | 6.9466 |  | protein phosphorylation |
| Cluster-10108.15711 | 1.5131 | 2.8229 | 1.328 | protein phosphorylation |
| Cluster-10108.5462 | 1.8983 | 3.9701 | 2.0923 | protein phosphorylation |
| Cluster-10108.15126 | 1.0359 | 2.322 | 1.3037 | protein phosphorylation |
| Cluster-10108.8909 | 2.1988 | 3.6773 |  | protein phosphorylation |
| Cluster-10108.20523 | 1.9576 | 2.4991 |  | protein phosphorylation |
| Cluster-10108.18610 | 1.8127 | 2.9686 |  | protein phosphorylation |
| Cluster-10108.6815 | 1.316 | 3.1283 | 1.8308 | protein phosphorylation |
| Cluster-10108.12644 | 1.4145 | 3.0856 | 1.6908 | protein phosphorylation |
| Cluster-10108.10430 | 2.9026 | 4.9584 |  | protein phosphorylation |
| Cluster-10108.5247 | 1.9235 | 2.3173 |  | protein phosphorylation |
| Cluster-10108.10932 | 1.5856 | 3.6825 | 2.1161 | protein phosphorylation |
| Cluster-10108.8282 | 3.4384 | 4.8882 |  | protein phosphorylation |
| Cluster-10108.15839 | 1.0565 | 3.3117 | 2.2725 | protein phosphorylation |
| Cluster-10108.16617 | 3.2005 | 4.1994 |  | regulation of transcription, DNA-templated//transmembrane transport |
| Cluster-10108.16017 | 1.225 | 1.0391 |  | regulation of transcription, DNA-templated |
| Cluster-10108.11949 | 2.771 | 4.9631 | 2.2101 | protein phosphorylation//transcription by RNA polymerase I |
| Cluster-10108.13294 | 3.3114 | 4.7065 |  | maturation of SSU-rRNA from tricistronic rRNA transcript (SSU-rRNA, 5.8S rRNA, LSU-rRNA)//DNA topological change |
| Cluster-10108.19547 | 3.6991 | 4.902 |  | -- |
| Cluster-10108.13815 | 2.4194 | 3.6211 |  | biosynthetic process |
| Cluster-10108.10223 | 2.7995 | 4.5721 |  | DNA replication//oxidation-reduction process |
| Cluster-10108.12646 | 2.3745 | 3.9738 |  | regulation of transcription, DNA-templated//translation//ribosome biogenesis |
| Cluster-10108.11144 | 3.8891 | 5.9178 |  | DNA replication initiation//regulation of transcription, DNA-templated |
| Cluster-10108.15166 | 4.1044 | 3.2351 |  | pathogenesis//regulation of transcription, DNA-templated |
| Cluster-10108.13317 | 1.0407 |  |  | mismatch repair//protein folding |
| Cluster-10108.8759 | 1.2822 |  |  | mismatch repair |
| Cluster-10108.7359 | 1.0608 | 1.4216 |  | mismatch repair//DNA replication initiation |
| Cluster-10108.11892 | 1.3296 | 1.062 |  | protein phosphorylation//regulation of transcription, DNA-templated |
| Cluster-10108.9730 | 3.4188 | 3.9947 |  | regulation of transcription, DNA-templated//protein ubiquitination |
| Cluster-10108.20838 | 1.8478 | 2.3545 |  | positive regulation of transcription elongation from RNA polymerase II promoter//histone deacetylation//transcription initiation from RNA polymerase II promoter |
| Cluster-10108.16609 | 1.3089 | 2.2422 |  | DNA recombination//DNA repair |
| Cluster-10108.17902 | 3.1961 | 4.9621 |  | DNA recombination//DNA repair |
| Cluster-10108.16455 | 1.0966 | 1.5649 |  | tRNA threonylcarbamoyladenosine modification//cellular biogenic amine metabolic process//DNA repair//DNA recombination//microtubule-based movement//spliceosomal complex assembly//microtubule-based process//regulation of transcription, DNA-templated |
| Cluster-10108.7804 | 1.9431 | 2.6272 |  | tRNA threonylcarbamoyladenosine modification//DNA repair//DNA recombination//viral genome replication//regulation of transcription, DNA-templated |
| Cluster-10108.12710 | 1.2744 | 1.8108 |  | tRNA threonylcarbamoyladenosine modification//DNA repair//DNA recombination//regulation of transcription, DNA-templated//DNA replication |
| Cluster-10108.4295 | 1.8743 | 2.8806 |  | DNA metabolic process |
| Cluster-10108.7369 | 2.0841 | 2.0819 |  | pyrimidine nucleotide biosynthetic process//DNA repair//pyrimidine nucleobase metabolic process |
| Cluster-10108.13492 | 1.0746 | 2.1077 |  | tRNA aminoacylation for protein translation//tRNA aminoacylation |
| Cluster-10108.13379 | 1.277 | 2.106 |  | base-excision repair//protein folding |
| Cluster-10108.1431 | -3.5258 | -4.5078 |  | DNA integration//positive regulation of Golgi to plasma membrane protein transport//protein phosphorylation |
| Cluster-10108.12898 | -1.9781 | -2.354 |  | regulation of transcription, DNA-templated |
| Cluster-10108.16434 | -2.2875 | -3.2222 |  | regulation of transcription, DNA-templated |
| Cluster-10108.23309 | -1.8667 |  | 1.8451 | regulation of transcription, DNA-templated//tRNA threonylcarbamoyladenosine modification |
| Cluster-10108.613 | -2.7009 | -3.6813 |  | regulation of transcription, DNA-templated//protein phosphorylation |
| Cluster-10108.20630 | -1.2726 | -1.677 |  | regulation of transcription, DNA-templated//signal transduction//obsolete acyl-carrier-protein biosynthetic process |
| Cluster-10108.12453 | -1.2225 | -1.6835 |  | sporulation resulting in formation of a cellular spore//mismatch repair |
| Cluster-10108.12864 | -3.3481 | -4.0468 |  | -- |
| Cluster-10108.16215 | -2.4841 | -2.8219 |  | -- |
| Cluster-10108.7522 | -2.1246 | -2.6448 |  | -- |
| Cluster-10108.12850 | -2.2013 | -2.626 |  | -- |
| Cluster-10108.1170 | -4.3788 | -4.7451 |  | double-strand break repair via nonhomologous end joining//phosphorylation//serine family amino acid metabolic process |
| Cluster-10108.14519 | -1.6209 | -1.766 |  | regulation of transcription, DNA-templated//phage shock//transport//transmembrane transport |
| Cluster-10108.16503 | -1.9843 | -2.598 |  | protein phosphorylation |
| Cluster-10108.13662 | -1.1426 |  |  | -- |
| Cluster-10108.16040 | -1.3465 |  |  | cytochrome complex assembly//nucleotide-excision repair//DNA repair//proteasome-mediated ubiquitin-dependent protein catabolic process//transport//heme transport//transmembrane transport |
| Cluster-10108.2782 | -2.9824 | -2.8324 |  | protein phosphorylation |
| Cluster-10108.6543 | -1.3024 | -1.6439 |  | protein phosphorylation |
| Cluster-10108.25409 | -3.9212 | -4.3741 |  | mitotic cell cycle//kinetochore assembly//protein phosphorylation |
| Cluster-10108.19822 | -1.9102 | -2.7296 |  | deoxyribonucleoside diphosphate metabolic process//purine nucleobase metabolic process//oxidation-reduction process//pyrimidine nucleobase metabolic process//DNA replication |
| Cluster-10108.14395 | -1.7431 | -2.3771 |  | transport//transmembrane transport |
| Cluster-10108.19690 | -1.9123 | -2.3612 |  | protein phosphorylation//recognition of pollen//phosphorylation//serine family amino acid metabolic process |
| Cluster-10108.5235 | -2.7671 | -3.0766 |  | protein phosphorylation//phosphorylation//serine family amino acid metabolic process |
| Cluster-10108.22482 | -3.627 | -5.166 | -1.5213 | protein phosphorylation//regulation of transcription, DNA-templated//DNA repair |
| Cluster-10108.12804 | -1.5219 | -2.1396 |  | -- |
| Cluster-10108.6947 | -1.2625 |  |  | protein phosphorylation//viral process//serine family amino acid metabolic process//phosphorylation |
| Cluster-10108.6769 | -1.486 | -1.5306 |  | amino acid transmembrane transport//regulation of transcription, DNA-templated//DNA replication initiation//signal transduction//cell adhesion//regulation of DNA replication |
| Cluster-10108.5731 | -1.5928 | -2.4364 |  | protein phosphorylation |
| Cluster-10108.20256 | -1.4788 | -2.2325 |  | protein phosphorylation |
| Cluster-10108.17084 | -1.581 | -1.7724 |  | pilus assembly//transcription, DNA-templated//protein phosphorylation |
| Cluster-10108.4489 | -3.9784 | -5.945 |  | protein phosphorylation//serine family amino acid metabolic process//phosphorylation |
| Cluster-10108.17154 | -2.8729 | -3.768 |  | protein phosphorylation |
| Cluster-10108.25404 | -2.8603 | -3.6232 |  | recognition of pollen//protein phosphorylation |
| Cluster-10108.18894 | -3.9777 | -5.9173 |  | mismatch repair |
| Cluster-10108.13725 | -1.0078 | -1.4364 |  | protein prenylation//protein phosphorylation |
| Cluster-10108.1439 | -3.596 | -4.3229 |  | protein phosphorylation//cell adhesion//tissue regeneration |
| Cluster-10108.12560 | -1.0337 | -1.4027 |  | protein phosphorylation |
| Cluster-10108.10301 | -1.4421 | -2.6009 | -1.142 | -- |
| Cluster-10108.7465 | -1.3372 | -2.3873 | -1.0317 | phosphorelay signal transduction system//protein phosphorylation//protein dephosphorylation//phosphorylation |
| Cluster-10108.17131 | -1.0197 | -1.3776 |  | protein phosphorylation |
| Cluster-10108.11057 | -1.3241 | -2.1003 |  | signal transduction//protein phosphorylation |
| Cluster-10108.19937 | -1.1378 | -1.4439 |  | tRNA aminoacylation for protein translation |
| Cluster-10108.21824 | -1.5049 | -3.1694 | -1.6482 | protein phosphorylation//phosphorylation//serine family amino acid metabolic process |
| Cluster-10108.12263 | -1.9752 | -2.9639 |  | menaquinone biosynthetic process |
| Cluster-10108.20345 | -1.7087 | -1.8015 |  | protein phosphorylation |
| Cluster-10108.18643 | -1.0365 |  |  | -- |
| Cluster-10108.3009 | -2.7692 | -3.6723 |  | protein phosphorylation |
| Cluster-10108.7093 | -2.3205 |  | 2.2286 | tRNA processing |
| Cluster-10108.10239 | -1.3906 | -2.3918 |  | nicotinate nucleotide metabolic process//DNA replication//NADP biosynthetic process//tRNA aminoacylation for protein translation//nicotinamide metabolic process |
| Cluster-10108.23990 | -1.2341 | -1.6688 |  | protein phosphorylation//phosphorylation//serine family amino acid metabolic process |
| Cluster-10108.23559 | -2.0474 | -2.4902 |  | protein phosphorylation//phosphorylation//serine family amino acid metabolic process |
| Cluster-10108.20513 | -1.9553 | -3.7774 | -1.8037 | protein phosphorylation |
| Cluster-10108.24701 | -2.0551 | -2.9922 |  | protein phosphorylation//phosphorylation//serine family amino acid metabolic process |
| Cluster-10108.9527 | -1.113 | -2.0521 |  | protein phosphorylation |
| Cluster-10108.2821 | -1.4775 | -2.028 |  | protein phosphorylation |
| Cluster-10108.12503 | -1.7108 | -2.1638 |  | recognition of pollen//protein phosphorylation//phosphorylation//serine family amino acid metabolic process |
| Cluster-10108.2389 | -2.1406 | -2.8335 |  | phosphorelay signal transduction system//DNA replication initiation//regulation of carbohydrate metabolic process//phosphorylation |
| Cluster-10108.11817 | -1.3257 | -1.8528 |  | protein phosphorylation |
| Cluster-10108.11250 | -1.0707 | -1.6418 |  | protein phosphorylation |
| Cluster-10108.7202 | -1.4726 | -2.4586 |  | protein phosphorylation//regulation of actin filament polymerization//Arp2/3 complex-mediated actin nucleation |
| Cluster-10108.19076 | -1.3562 | -2.7173 | -1.3428 | protein phosphorylation//defense response to virus//signal transduction//RNA polyadenylation//positive regulation of immune response//receptor signaling pathway via JAK-STAT |
| Cluster-10108.3844 | -1.121 | -1.5587 |  | protein phosphorylation |
| Cluster-10108.25142 | -1.8061 | -2.2732 |  | protein phosphorylation |
| Cluster-10108.1806 | -1.1662 | -1.5181 |  | protein phosphorylation |
| Cluster-10108.7732 | -1.9981 | -2.443 |  | protein phosphorylation |
| Cluster-10108.2918 | -1.4913 | -1.5507 |  | protein phosphorylation//recognition of pollen |
| Cluster-10108.1755 | -5.0004 | -6.8623 |  | protein phosphorylation |
| Cluster-10108.26059 | -8.2926 |  |  | cellular biogenic amine metabolic process |
| Cluster-10108.29538 | -1.3267 | -2.0415 |  | coenzyme A biosynthetic process//pantothenate biosynthetic process//pyrimidine nucleobase metabolic process//nucleobase-containing compound metabolic process |
| Cluster-10108.4550 | -2.3465 | -2.9438 |  | protein phosphorylation//DNA packaging//recognition of pollen//serine family amino acid metabolic process//phosphorylation |
| Cluster-10108.24366 | -2.1599 | -3.6094 |  | protein phosphorylation |
| Cluster-10108.24405 | -2.1879 | -3.5302 |  | protein phosphorylation |
| Cluster-10108.3562 | -1.3447 | -2.3383 |  | protein phosphorylation//serine family amino acid metabolic process//phosphorylation |
| Cluster-10108.2982 | -1.2318 |  |  | protein phosphorylation |
| Cluster-10108.23211 | -1.2137 | -1.6881 |  | protein phosphorylation |
| Cluster-10108.4311 | -1.4094 |  |  | protein phosphorylation |
| Cluster-10108.13902 | -1.8201 | -1.7582 |  | -- |
| Cluster-10108.3251 | -2.4563 | -3.0684 |  | -- |
| Cluster-10108.5951 | -1.5394 | -1.3123 |  | -- |
| Cluster-10108.17580 | -2.0776 | -2.3085 |  | DNA repair//DNA recombination |
| Cluster-10108.4502 | -1.6 | -2.5337 |  | -- |
| Cluster-10108.387 | -3.1551 | -1.9796 |  | -- |
| Cluster-10108.20308 | -1.2249 | -1.7043 |  | DNA recombination//DNA repair |
| Cluster-10108.22019 | -1.7527 | -1.96 |  | tRNA threonylcarbamoyladenosine modification |
| Cluster-10108.21391 | -1.0076 | -1.9426 |  | double-strand break repair via single-strand annealing, removal of nonhomologous ends//intracellular signal transduction |
| Cluster-10108.6304 | -2.1887 | -2.4216 |  | arginine metabolic process//peptidyl-proline hydroxylation to 4-hydroxy-L-proline//proline metabolic process//oxidation-reduction process |
| Cluster-10108.3927 |  | -1.1894 |  | transport |
| Cluster-10108.15768 |  | 1.1237 |  | regulation of transcription, DNA-templated//DNA repair//tRNA threonylcarbamoyladenosine modification//DNA recombination |
| Cluster-10108.20608 |  | -1.5707 |  | regulation of transcription, DNA-templated//conjugation |
| Cluster-10108.6938 |  | 1.6524 |  | negative regulation of biosynthetic process//viral DNA genome packaging |
| Cluster-10108.1728 |  | -5.4558 |  | visual perception//purine nucleobase metabolic process |
| Cluster-10108.13753 |  | 1.4632 |  | oxidation-reduction process |
| Cluster-10108.4611 |  | 1.4738 |  | transposition, DNA-mediated//intracellular protein transport//proteolysis |
| Cluster-10108.14394 |  | 1.3059 |  | -- |
| Cluster-10108.19745 |  | 1.531 |  | transport//transmembrane transport |
| Cluster-10108.2040 |  | -6.2939 |  | spermatogenesis |
| Cluster-10108.22028 |  | 2.7734 |  | -- |
| Cluster-10108.29578 |  | -4.1214 |  | -- |
| Cluster-10108.18723 |  | -1.3082 |  | tRNA threonylcarbamoyladenosine modification//transport//transmembrane transport//regulation of apoptotic process |
| Cluster-10108.13655 |  | -1.0758 |  | angiotensin-activated signaling pathway |
| Cluster-10108.16081 |  | -1.4295 |  | chromosome segregation//chromosome condensation//oxidation-reduction process//SRP-dependent cotranslational protein targeting to membrane//nitrogen compound metabolic process |
| Cluster-10108.26793 |  | 2.0618 |  | tRNA processing//RNA modification |
| Cluster-10108.15528 |  | 1.0555 |  | fat cell differentiation//mitigation of host immune response by virus//G protein-coupled receptor signaling pathway |
| Cluster-10108.12793 |  | 1.2648 |  | proton transmembrane transport//ATP metabolic process//ATP biosynthetic process |
| Cluster-536.0 |  | -6.5224 |  | -- |
| Cluster-10108.12085 |  | 4.7824 | 3.727 | rRNA methylation//RNA processing//pathogenesis//rRNA modification |
| Cluster-10108.1247 |  | -4.3799 | -1.9095 | arginine metabolic process//pyruvate metabolic process//sodium ion transport//proline metabolic process//intracellular protein transport//sodium ion export across plasma membrane |
| Cluster-10108.14264 |  | 1.0658 |  | -- |
| Cluster-7876.0 |  | 5.1539 |  | chromatin remodeling |
| Cluster-10108.9252 |  | 2.0263 | 1.3849 | cellular biogenic amine metabolic process//DNA replication initiation |
| Cluster-10108.9174 |  | 1.3843 |  | ATP synthesis coupled proton transport |
| Cluster-10108.12222 |  | -1.0655 |  | -- |
| Cluster-10108.19738 |  | -2.768 |  | -- |
| Cluster-10108.8854 |  | 2.729 | 1.8475 | selenocysteine incorporation//regulation of translational elongation |
| Cluster-10108.13375 |  | 1.297 |  | obsolete electron transport//photosynthesis |
| Cluster-10108.10128 |  | 1.6328 |  | -- |
| Cluster-10108.8895 |  | -1.2459 |  | DNA recombination//meiotic DNA double-strand break formation//asparagine metabolic process |
| Cluster-4382.0 |  | -5.3522 |  | -- |
| Cluster-10108.14789 |  | -1.0623 |  | -- |
| Cluster-10108.2055 |  | -9.0946 |  | tRNA threonylcarbamoyladenosine modification//proton transmembrane transport//ATP metabolic process |
| Cluster-10108.16093 |  | 1.9783 | 1.4112 | regulation of transcription, DNA-templated//proteolysis//DNA recombination//DNA repair |
| Cluster-10108.7007 | 2.1189 | 1.7066 |  | tRNA threonylcarbamoyladenosine modification |
| Cluster-10108.22144 | 3.9218 | 3.4281 |  | tRNA threonylcarbamoyladenosine modification |
| Cluster-10108.17277 | 1.5954 | 2.0832 |  | -- |
| Cluster-10108.16121 | 1.968 | 4.541 | 2.5941 | -- |
| Cluster-10108.11448 | 1.3335 | 2.7465 | 1.4328 | -- |
| Cluster-10108.8962 | 1.0229 | 1.4039 |  | -- |
| Cluster-10108.17275 | 1.5607 | 4.3332 | 2.7918 | -- |
| Cluster-10108.16012 | 1.2333 | 1.8469 |  | phosphate ion transport |
| Cluster-10108.17634 | 1.1821 | 2.0244 |  | Mo-molybdopterin cofactor biosynthetic process |
| Cluster-10108.14076 | -1.4056 | -1.6844 |  | tRNA processing |
| Cluster-10108.8021 | -1.5911 | -1.6515 |  | -- |
| Cluster-10108.15829 | -1.5823 | -1.7053 |  | mRNA processing//regulation of transcription, DNA-templated |
| Cluster-10108.16833 | -1.5213 | -2.0409 |  | -- |
| Cluster-10108.13600 | -1.772 | -2.3851 |  | transmembrane transport//transport |
| Cluster-10108.8376 | -1.8037 | -2.1626 |  | tRNA threonylcarbamoyladenosine modification |
| Cluster-10108.12155 |  | 1.2871 |  | ATP synthesis coupled proton transport |
| Cluster-839.0 |  | -6.5551 |  | ATP synthesis coupled proton transport |
| Cluster-10108.25805 |  | -5.7997 |  | ATP synthesis coupled proton transport |
| Cluster-10108.13174 |  | -1.423 |  | ATP synthesis coupled proton transport//cilium assembly//transcription elongation from RNA polymerase II promoter//cilium movement involved in cell motility//regulation of transcription, DNA-templated//histone modification//proteolysis//transcription by RNA polymerase III//neuronal signal transduction |
| Cluster-10108.7748 |  | -1.4548 |  | ATP synthesis coupled proton transport//phosphorylation//serine family amino acid metabolic process//protein phosphorylation |
| Cluster-10108.10137 |  | 1.0923 |  | ATP synthesis coupled electron transport//mitochondrial electron transport, NADH to ubiquinone//ubiquinone biosynthetic process//sodium ion transport |
| Cluster-10108.15695 |  | 2.2869 | 1.4718 | ATP synthesis coupled proton transport//intracellular protein transport//endoplasmic reticulum to Golgi vesicle-mediated transport |
| Cluster-10108.26282 |  | -6.6045 |  | ATP synthesis coupled proton transport |
| Cluster-10108.2678 |  | 3.3974 |  | ATP synthesis coupled proton transport//photosynthesis |
| Cluster-10108.26103 |  | -6.1174 |  | ATP synthesis coupled proton transport |
| Cluster-10108.14874 |  | -1.0614 |  | ATP synthesis coupled proton transport//regulation of transcription, DNA-templated |
| Cluster-10108.19094 |  | -1.5075 |  | ATP synthesis coupled proton transport |
| Cluster-7242.0 |  | -4.8862 |  | ATP synthesis coupled proton transport |
| Cluster-10108.14555 |  | 1.2905 | 1.5118 | ATP synthesis coupled proton transport//sodium ion export across plasma membrane//arginine metabolic process//sodium ion transport//pyruvate metabolic process//cell redox homeostasis//protein transport//proline metabolic process |
| Cluster-522.0 |  | -6.5607 |  | ATP synthesis coupled proton transport |
| Cluster-10108.18883 |  | -1.6559 |  | ATP synthesis coupled proton transport |
| Cluster-10108.7477 |  | -1.513 |  | ATP synthesis coupled proton transport |
| Cluster-10108.155 |  | -6.2531 |  | ATP synthesis coupled proton transport |
| Cluster-10108.18505 |  | -2.1075 |  | ATP synthesis coupled proton transport |
| Cluster-10108.20549 |  | -1.2873 |  | ATP synthesis coupled proton transport//oxidative phosphorylation//proton transmembrane transport |
| Cluster-10108.8822 |  | 1.0753 |  | ATP synthesis coupled proton transport//carbohydrate metabolic process |
| Cluster-10108.15890 |  | -1.4421 |  | ATP synthesis coupled proton transport |
| Cluster-10108.21642 |  | 1.1729 |  | ATP synthesis coupled proton transport |
| Cluster-10108.22495 |  | 3.5278 |  | ATP synthesis coupled proton transport |
| Cluster-10108.4543 |  | -1.2275 |  | ATP synthesis coupled proton transport |
| Cluster-10108.15617 |  | -1.0207 |  | ATP synthesis coupled proton transport |
| Cluster-10108.14640 |  | 1.8255 | 1.0276 | ATP synthesis coupled proton transport//signal transduction |
| Cluster-10108.21095 | -2.2658 | -2.3927 |  | ATP synthesis coupled proton transport |
| Cluster-10108.15335 | -2.084 | -2.3673 |  | ATP synthesis coupled proton transport |
| Cluster-10108.7178 | -1.977 | -2.6973 |  | ATP synthesis coupled proton transport//transmembrane transport//metal ion transport |
| Cluster-10108.2251 | -3.1576 | -4.3058 |  | ATP synthesis coupled proton transport |
| Cluster-10108.23208 | -1.3946 | -1.5524 |  | ATP synthesis coupled proton transport |
| Cluster-10108.18299 | -1.4078 | -1.491 |  | ATP synthesis coupled proton transport//ATP metabolic process//proton transmembrane transport |
| Cluster-10108.988 | -7.9414 | -7.2607 |  | ATP synthesis coupled proton transport |
| Cluster-10108.26380 | -2.2349 | -3.0125 |  | ATP synthesis coupled proton transport |
| Cluster-10108.11315 | -2.0802 | -2.944 |  | ATP synthesis coupled proton transport |
| Cluster-10108.3695 | 3.0726 | 4.1064 |  | ATP synthesis coupled proton transport//regulation of DNA-templated transcription, termination//transcriptional attenuation by ribosome |
| Cluster-10108.19754 | 1.2034 | 3.4257 | 2.2402 | ATP synthesis coupled proton transport//sucrose metabolic process//cell wall modification//starch metabolic process |
| Cluster-10108.18913 | 3.5188 | 5.3915 |  | ATP synthesis coupled proton transport |
| Cluster-10108.21774 | 1.8682 | 3.2465 |  | ATP synthesis coupled proton transport |
| Cluster-10108.4768 | 2.351 | 4.8243 |  | ATP synthesis coupled proton transport |
| Cluster-10108.12414 | 1.0606 | 1.7696 |  | ATP synthesis coupled proton transport |
| Cluster-10108.19479 | 1.6963 | 2.5496 |  | ATP synthesis coupled proton transport |
| Cluster-10108.18977 | 1.4416 | 2.2191 |  | ATP synthesis coupled proton transport |
| Cluster-10108.407 |  | -3.4501 |  | polyketide biosynthetic process |
| Cluster-10108.6665 |  | -1.0573 |  | polyketide biosynthetic process//obsolete electron transport//photosynthesis, light reaction |
| Cluster-10108.7536 |  | -1.4397 |  | transformation of host cell by virus//photosynthesis//photosynthesis, light reaction |
| Cluster-10108.11765 |  | -1.3305 |  | photosynthesis |
| Cluster-10108.21657 |  | -2.4785 |  | photosynthesis//photosystem II stabilization |
| Cluster-10108.8997 |  | -1.1242 |  | photosynthesis//photosynthesis, light reaction//gamete generation |
| Cluster-10108.11406 |  | 1.4166 |  | photosynthesis//photosystem II assembly |
| Cluster-10108.14952 |  | 1.0437 |  | photosynthesis//ribosome biogenesis//translation//regulation of transcription, DNA-templated |
| Cluster-10108.5449 |  | 2.8917 |  | photosynthesis//signal transduction |
| Cluster-10108.12698 |  | 1.0712 |  | photosynthesis |
| Cluster-10108.24984 |  | 5.2487 | 4.2368 | photosynthesis//photosystem II stabilization |
| Cluster-10108.23728 |  | 2.0923 | 2.3919 | photosynthesis |
| Cluster-10108.8812 |  | -1.3158 |  | photosynthesis//obsolete acyl-carrier-protein biosynthetic process//lipid metabolic process |
| Cluster-10108.24994 |  | 3.912 |  | photosynthesis//gluconeogenesis//ammonium transport |
| Cluster-10108.13746 |  | 1.459 |  | photosynthesis |
| Cluster-10108.23400 |  | -4.8884 |  | photosynthesis |
| Cluster-10108.4191 |  | 4.8705 |  | photosynthesis//photosystem II stabilization |
| Cluster-10108.17263 |  | -1.508 |  | photosynthesis//transformation of host cell by virus |
| Cluster-10108.20998 |  | 3.1042 | 2.6065 | photosynthesis |
| Cluster-10108.12742 |  | -2.4843 | -1.5574 | photosynthesis//rRNA modification//RNA processing//rRNA processing |
| Cluster-10108.28517 |  | -2.7934 |  | photosynthesis |
| Cluster-10108.53 |  | -4.8353 |  | -- |
| Cluster-10108.12534 |  | 2.5369 | 1.6486 | carbon utilization//oxidation-reduction process//carbohydrate metabolic process//glycolytic process//gluconeogenesis//purine nucleobase metabolic process |
| Cluster-10108.15403 |  | 2.2859 | 1.627 | carbon utilization//signal transduction//glycolytic process//gluconeogenesis//purine nucleobase metabolic process |
| Cluster-10108.84 |  | -4.5008 |  | photosystem II stabilization//photosynthesis |
| Cluster-10108.16641 |  | 1.2292 |  | photosystem II assembly |
| Cluster-10108.2833 |  | -1.2 |  | photosynthetic electron transport chain//photosynthesis, light reaction |
| Cluster-10108.10238 |  | 1.2628 |  | DNA repair//DNA replication |
| Cluster-10108.24536 |  | -1.5095 |  | tRNA threonylcarbamoyladenosine modification |
| Cluster-10108.1711 |  | -3.0776 |  | -- |
| Cluster-10108.23214 |  | -1.1487 |  | -- |
| Cluster-10108.22036 |  | -1.6457 |  | arginine metabolic process//peptidyl-proline hydroxylation to 4-hydroxy-L-proline//DNA repair//proline metabolic process//oxidation-reduction process//DNA recombination |
| Cluster-10108.19835 |  | 1.3391 |  | -- |
| Cluster-10108.17388 |  | 1.2091 |  | -- |
| Cluster-10108.6241 |  | 1.0405 |  | obsolete acyl-carrier-protein biosynthetic process |
| Cluster-10108.7437 |  | -2.2965 |  | arginine catabolic process//proline metabolic process//phosphorelay signal transduction system//obsolete acyl-carrier-protein biosynthetic process |
| Cluster-10108.19179 |  | 1.2019 |  | -- |
| Cluster-10108.11810 |  | -1.4737 |  | ion transport//transmembrane transport//transport |
| Cluster-10108.8981 |  | 1.155 |  | pyrimidine nucleobase metabolic process//DNA repair//nucleobase-containing compound metabolic process//DNA recombination |
| Cluster-10108.13341 |  | 1.2486 | 1.0993 | DNA repair//pathogenesis//DNA recombination//quorum sensing//proteolysis |
| Cluster-10108.6540 |  | -2.69 |  | viral process |
| Cluster-473.0 |  | -5.518 |  | -- |
| Cluster-10108.16181 |  | 2.0211 |  | cellular biogenic amine metabolic process//DNA replication initiation |
| Cluster-10108.11362 |  | 3.0154 |  | -- |
| Cluster-10108.5983 |  | 1.8583 |  | DNA repair//DNA recombination |
| Cluster-10108.11537 |  | -1.443 |  | -- |
| Cluster-10108.15560 |  | -1.1154 |  | pyrimidine nucleobase metabolic process//nucleobase-containing compound metabolic process |
| Cluster-10108.4714 |  | 1.9723 |  | chondroitin sulfate biosynthetic process |
| Cluster-10108.12312 |  | -1.2431 |  | -- |
| Cluster-10108.12775 |  | 1.0577 |  | -- |
| Cluster-10108.5439 |  | 1.9289 |  | -- |
| Cluster-10108.4741 |  | 2.4706 |  | -- |
| Cluster-10108.4766 |  | 1.2725 |  | -- |
| Cluster-10108.10324 |  | -1.3978 |  | -- |
| Cluster-10108.11707 |  | -1.0991 |  | -- |
| Cluster-10108.12388 |  | 1.2574 | 1.2795 | intrinsic apoptotic signaling pathway//magnesium ion transport//fatty acid biosynthetic process//pyruvate metabolic process |
| Cluster-10108.15600 |  | -1.228 |  | -- |
| Cluster-10108.13301 |  | 1.0046 |  | gluconeogenesis//phosphorylation//defense response to Gram-positive bacterium//glycolytic process//carbon utilization//phosphorelay signal transduction system |
| Cluster-10108.12674 |  | -1.1488 |  | amine metabolic process//chorion-containing eggshell formation//oxidation-reduction process//mannose metabolic process//carbohydrate metabolic process |
| Cluster-10108.3216 |  | 2.902 |  | nitrogen compound metabolic process |
| Cluster-10108.3481 |  | 3.5424 |  | nitrogen compound metabolic process |
| Cluster-10108.9561 |  | 1.5241 |  | nitrogen compound metabolic process//oxidation-reduction process |
| Cluster-10108.20675 |  | 1.7461 |  | nitrogen compound metabolic process//carbohydrate metabolic process |
| Cluster-10108.20071 |  | -1.4581 |  | -- |
| Cluster-10108.18803 |  | 1.6169 | 1.0734 | glucose catabolic process//gluconeogenesis//glycolytic process |
| Cluster-10108.16448 |  | 1.6454 |  | -- |
| Cluster-10108.20082 |  | 1.1734 |  | glycosphingolipid metabolic process//galactose metabolic process//glycerolipid metabolic process//glycosaminoglycan catabolic process//biosynthetic process//carbohydrate metabolic process |
| Cluster-10108.16581 |  | 1.3782 |  | glycosphingolipid metabolic process//galactose metabolic process//glycerolipid metabolic process//glycosaminoglycan catabolic process//nuclear-transcribed mRNA catabolic process, nonsense-mediated decay//carbohydrate metabolic process |
| Cluster-10108.7171 |  | 1.7467 |  | light absorption |
| Cluster-10108.11476 |  | 1.5079 | 1.0839 | -- |
| Cluster-10108.12690 |  | 2.0398 | 1.2496 | chlorophyll metabolic process//chlorophyll catabolic process |
| Cluster-10108.10226 |  | 1.2713 | 1.1821 | chlorophyll metabolic process//porphyrin-containing compound biosynthetic process//activation of cysteine-type endopeptidase activity involved in apoptotic process//apoptotic process |
| Cluster-10108.15688 |  | 2.7445 |  | -- |
| Cluster-10108.6493 |  | 1.3434 |  | -- |
| Cluster-10108.8412 |  | 2.0791 |  | oligosaccharide biosynthetic process |
| Cluster-10108.4439 |  | -1.0592 |  | oligosaccharide biosynthetic process |
| Cluster-2771.0 |  | -6.17 |  | -- |
| Cluster-10108.22622 |  | 3.5262 |  | -- |
| Cluster-10108.8873 |  | 1.211 |  | -- |
| Cluster-10108.11109 |  | 1.0357 |  | -- |
| Cluster-10108.17870 |  | 1.5028 |  | -- |
| Cluster-10108.27291 |  | -6.495 |  | mitochondrial electron transport, ubiquinol to cytochrome c |
| Cluster-10108.8663 |  | -1.0311 |  | DNA topological change//phosphoenolpyruvate-dependent sugar phosphotransferase system//carbohydrate transport//protein processing |
| Cluster-10108.9892 |  | -1.0699 |  | -- |
| Cluster-10108.6380 |  | 1.8396 |  | -- |
| Cluster-10108.20080 |  | -2.0239 |  | -- |
| Cluster-10108.12696 |  | -1.179 |  | negative regulation of ATPase activity |
| Cluster-10108.10064 |  | 1.0523 |  | bacteriochlorophyll biosynthetic process//photosynthesis, dark reaction |
| Cluster-10108.17436 |  | 1.7105 |  | carbon utilization//malate metabolic process//oxidation-reduction process//tricarboxylic acid cycle//pyruvate metabolic process |
| Cluster-10108.28229 |  | -2.9342 |  | cellular glucan metabolic process |
| Cluster-10108.13577 |  | -1.6535 |  | -- |
| Cluster-10108.3668 | 3.7514 | 4.0908 |  | regulation of GTPase activity |
| Cluster-10108.10615 | 1.0059 | 1.6032 |  | regulation of GTPase activity//small GTPase mediated signal transduction |
| Cluster-10108.21836 | 1.1556 | 1.7916 |  | regulation of GTPase activity |

# Table S7 Genes Related to Protein and Amino Acid Metabolism

| gene_id | T1VST3 | T1vsT4 | T3VST4 | BP Description |
| --- | --- | --- | --- | --- |
| Cluster-10108.11026 | 2.5966 | 4.4923 | 1.9158 | ribosome biogenesis//pseudouridine synthesis |
| Cluster-10108.10589 | 1.5367 | 2.2795 | 0.76305 | protein targeting//intracellular protein transport//response to antibiotic |
| Cluster-10108.9189 | 4.873 | 5.6886 | 0.83303 | ribosome biogenesis//translation |
| Cluster-10108.9366 | 2.8996 | 3.4511 | 0.57088 | translation//ribosome biogenesis |
| Cluster-10108.16770 | 3.7524 | 4.7972 | 1.0657 | ribosome biogenesis//translation |
| Cluster-10108.10120 | 1.3721 | 1.796 | 0.44358 | ribosome biogenesis |
| Cluster-10108.7266 | 1.2034 | 1.7972 | 0.61214 | translation//ribosome biogenesis |
| Cluster-10108.8353 | 1.4277 | 2.1937 | 0.78645 | ribosome biogenesis//translation |
| Cluster-10108.14354 | 1.9228 | 2.4283 | 0.52366 | ribosome biogenesis//translation |
| Cluster-10108.8861 | 1.0846 | 1.3768 | 0.31176 | ribosome biogenesis//translation |
| Cluster-10108.17373 | 1.2245 | 2.1207 | 0.91369 | ribosome biogenesis//translation |
| Cluster-10108.14730 | 1.4227 | 2.3882 | 0.98651 | translation//ribosome biogenesis |
| Cluster-10108.13097 | 1.2216 | 1.8914 | 0.6894 | ribosome biogenesis//translation |
| Cluster-10108.12154 | 1.1022 | 1.8866 | 0.80482 | translation//ribosome biogenesis |
| Cluster-10108.12317 | 1.176 | 1.6685 | 0.51228 | ribosome biogenesis//translation |
| Cluster-10108.12844 | 1.6147 | 2.911 | 1.3172 | ribosome biogenesis//translation |
| Cluster-10108.14434 | 1.4607 | 2.5376 | 1.0978 | ribosome biogenesis//translation |
| Cluster-10108.11880 | 1.2998 | 2.2177 | 0.93764 | translation//ribosome biogenesis |
| Cluster-10108.13663 | 1.1573 | 2.0155 | 0.87786 | translation//ribosome biogenesis |
| Cluster-10108.9519 | 1.1403 | 3.4252 | 2.3024 | protein trimerization//ribosome biogenesis//translation |
| Cluster-10108.15232 | 1.2866 | 2.2376 | 0.97063 | translation//ribosome biogenesis |
| Cluster-10108.16444 | 1.2656 | 1.9347 | 0.68938 | translation//ribosome biogenesis |
| Cluster-10108.7850 | 1.3679 | 2.0763 | 0.72931 | ribosome biogenesis//translation |
| Cluster-10108.17817 | 1.0971 | 1.8821 | 0.80434 | ribosome biogenesis//RNA interference//translation//RNA transport |
| Cluster-10108.13500 | 1.3967 | 2.6172 | 1.2416 | ribosome biogenesis//translation |
| Cluster-10108.13541 | 1.2796 | 2.1278 | 0.86784 | translation//ribosome biogenesis |
| Cluster-10108.20116 | 1.0628 | 1.7477 | 0.70394 | ribosome biogenesis//translation |
| Cluster-10108.13129 | 1.2289 | 1.9302 | 0.72135 | translation//ribosome biogenesis |
| Cluster-10108.11080 | 1.2931 | 2.4956 | 1.2222 | translation//ribosome biogenesis |
| Cluster-10108.13733 | 1.1584 | 1.9829 | 0.8442 | ribosome biogenesis//translation |
| Cluster-10108.9417 | 1.2457 | 2.1661 | 0.94064 | translation//ribosome biogenesis |
| Cluster-10108.11960 | 1.204 | 1.9101 | 0.72692 | translation//ribosome biogenesis |
| Cluster-10108.9389 | 1.6198 | 2.1675 | 0.56895 | translation//ribosome biogenesis |
| Cluster-10108.2852 | 1.4418 | 2.5173 | 1.0953 | ribosome biogenesis//translation |
| Cluster-10108.14000 | 1.2425 | 2.1306 | 0.90803 | ribosome biogenesis//translation |
| Cluster-10108.11804 | 1.1395 | 1.9259 | 0.80607 | ribosome biogenesis//translation |
| Cluster-10108.14143 | 1.2206 | 2.03 | 0.82999 | translation//ribosome biogenesis |
| Cluster-10108.13422 | 1.3493 | 2.4311 | 1.1025 | translation//ribosome biogenesis |
| Cluster-10108.12628 | 1.1841 | 2.2459 | 1.0812 | ribosome biogenesis//translation |
| Cluster-10108.2979 | 2.2372 | 2.4616 | 0.24243 | translation//ribosome biogenesis |
| Cluster-10108.6983 | 1.3232 | 1.3381 | 0.035237 | ribosome biogenesis//translation |
| Cluster-10108.14117 | 1.0874 | 1.6462 | 0.57929 | translation//ribosome biogenesis |
| Cluster-10108.15748 | 1.0579 | 1.9127 | 0.87379 | ribosome biogenesis//translation//cellular metabolic process |
| Cluster-10108.12067 | 1.1734 | 2.1658 | 1.0127 | ribosome biogenesis//translation |
| Cluster-10108.14756 | 1.1475 | 2.1878 | 1.0601 | ribosome biogenesis//translation |
| Cluster-10108.14759 | 1.0679 | 1.6634 | 0.61532 | ribosome biogenesis//translation |
| Cluster-10108.6859 | 1.0312 | 0.7473 | -0.26523 | ribosome biogenesis//translation |
| Cluster-10108.12601 | 1.2099 | 2.492 | 1.3024 | ribosome biogenesis//translation |
| Cluster-10108.12407 | 1.0386 | 1.914 | 0.8949 | ribosome biogenesis//translation |
| Cluster-10108.13296 | 1.1393 | 2.0111 | 0.89273 | ribosome biogenesis//translation |
| Cluster-10108.5165 | 2.321 | 4.6305 | 2.3278 | ribosome biogenesis//translation |
| Cluster-10108.14115 | 1.3024 | 2.7898 | 1.5076 | ribosome biogenesis//translation |
| Cluster-10108.12283 | 1.0171 | 1.9555 | 0.95878 | ribosome biogenesis//translation |
| Cluster-10108.13616 | 1.1696 | 2.0289 | 0.87953 | ribosome biogenesis//translation |
| Cluster-10108.11621 | 1.0457 | 1.874 | 0.84774 | ribosome biogenesis//translation |
| Cluster-10108.13665 | 1.1277 | 2.0826 | 0.97469 | translation//ribosome biogenesis |
| Cluster-10108.12902 | 1.0829 | 2.0249 | 0.96231 | translation//ribosome biogenesis |
| Cluster-10108.15098 | 2.8049 | 4.5486 | 1.7534 | translation//ribosome biogenesis |
| Cluster-10108.14188 | 1.1434 | 2.2455 | 1.1226 | ribosome biogenesis//translation |
| Cluster-10108.23582 | 1.2731 | 1.8431 | 0.58895 | ribosome biogenesis//translation |
| Cluster-10108.14222 | 1.1585 | 2.384 | 1.2452 | translation//ribosome biogenesis |
| Cluster-10108.10995 | 1.008 | 1.9946 | 1.0068 | ribosome biogenesis//translation//calcium-mediated signaling |
| Cluster-10108.12791 | 2.0731 | 2.1342 | 0.079391 | ribosome biogenesis//translation//ubiquitin-dependent protein catabolic process |
| Cluster-10108.8562 | 4.179 | 5.235 | 1.0715 | protein ubiquitination |
| Cluster-10108.9631 | 1.0943 | 2.0577 | 0.98252 | protein ubiquitination |
| Cluster-10108.8139 | 4.3987 | 5.1014 | 0.72216 | protein phosphorylation |
| Cluster-10108.14818 | 1.1074 | 1.7097 | 0.6199 | protein phosphorylation |
| Cluster-10108.9879 | 1.3373 | 1.5944 | 0.27545 | protein phosphorylation |
| Cluster-10108.13071 | 1.0624 | 1.6018 | 0.55784 | protein phosphorylation |
| Cluster-10108.7698 | 1.4311 | 2.6828 | 1.2709 | protein phosphorylation |
| Cluster-10108.9216 | 1.9674 | 4.0223 | 2.0727 | protein phosphorylation |
| Cluster-10108.10290 | 1.4184 | 4.342 | 2.944 | protein phosphorylation |
| Cluster-10108.9198 | 3.197 | 4.0002 | 0.8231 | protein phosphorylation |
| Cluster-10108.8604 | 1.8165 | 2.8583 | 1.0595 | protein phosphorylation |
| Cluster-10108.20610 | 1.7243 | 3.0348 | 1.3246 | protein phosphorylation |
| Cluster-10108.7610 | 4.0028 | 6.1107 | 2.1342 | protein phosphorylation |
| Cluster-10108.11115 | 3.4326 | 4.1556 | 0.74432 | protein phosphorylation |
| Cluster-10108.11327 | 3.0524 | 3.4335 | 0.39949 | protein phosphorylation |
| Cluster-10108.9479 | 1.2364 | 2.354 | 1.1354 | protein phosphorylation |
| Cluster-10108.5074 | 3.4566 | 5.9349 | 2.4903 | protein phosphorylation |
| Cluster-10108.9065 | 2.4149 | 2.3024 | -0.09413 | protein phosphorylation |
| Cluster-10108.9138 | 1.6748 | 3.0114 | 1.3546 | protein phosphorylation |
| Cluster-10108.15254 | 2.0191 | 3.1417 | 1.1418 | protein phosphorylation |
| Cluster-10108.17140 | 1.577 | 3.0993 | 1.5396 | protein phosphorylation |
| Cluster-10108.14866 | 1.5055 | 2.8479 | 1.361 | protein phosphorylation |
| Cluster-10108.13540 | 1.6439 | 2.7741 | 1.1479 | protein phosphorylation |
| Cluster-10108.6789 | 3.7611 | 5.6546 | 1.919 | protein phosphorylation |
| Cluster-10108.5736 | 2.6679 | 3.9365 | 1.2896 | protein phosphorylation |
| Cluster-10108.14578 | 1.5905 | 1.8004 | 0.22903 | protein phosphorylation |
| Cluster-10108.18848 | 2.0179 | 3.5426 | 1.5431 | protein phosphorylation |
| Cluster-10108.16661 | 1.5398 | 3.4057 | 1.8846 | protein phosphorylation |
| Cluster-10108.14435 | 2.8395 | 7.0804 | 4.2706 | protein phosphorylation |
| Cluster-10108.6091 | 1.3712 | 1.4538 | 0.1011 | protein phosphorylation |
| Cluster-10108.14692 | 1.4624 | 2.0421 | 0.59838 | protein phosphorylation |
| Cluster-10108.15113 | 1.173 | 3.5174 | 2.3618 | protein phosphorylation |
| Cluster-10108.16648 | 1.4323 | 5.554 | 4.1384 | protein phosphorylation |
| Cluster-10108.6586 | 3.4448 | 2.846 | -0.57988 | protein deubiquitination |
| Cluster-10108.4823 | 3.5724 | 5.6849 | 2.1346 | protein homooligomerization//DNA repair//carbohydrate metabolic process |
| Cluster-10108.16269 | 1.1287 | 1.4215 | 0.3109 | protein methylation//lysine catabolic process//histone lysine methylation |
| Cluster-10108.10148 | 2.1523 | 2.2744 | 0.14173 | translational initiation |
| Cluster-10108.11637 | 2.3742 | 2.6821 | 0.32761 | protein maturation |
| Cluster-10108.9793 | 2.2038 | 4.8937 | 2.7118 | protein maturation |
| Cluster-10108.15251 | 1.674 | 2.5061 | 0.85284 | translation//ribosome biogenesis |
| Cluster-10108.16486 | 2.0303 | 3.0142 | 1.0041 | translation//ribosome biogenesis |
| Cluster-10108.14697 | 1.4309 | 2.5059 | 1.095 | translation//ribosome biogenesis |
| Cluster-10108.12960 | 1.3172 | 2.4578 | 1.1609 | translation//ribosome biogenesis |
| Cluster-10108.15230 | 1.1008 | 2.157 | 1.0754 | translation//ribosome biogenesis |
| Cluster-10108.23540 | 7.0508 | 6.9937 | -0.03599 | protein ubiquitination |
| Cluster-10108.18507 | 1.535 | 3.0696 | 1.5528 | ribosome biogenesis//translation |
| Cluster-10108.12420 | 1.4622 | 1.8687 | 0.42586 | protein peptidyl-prolyl isomerization |
| Cluster-10108.11437 | 1.2642 | 1.8534 | 0.60927 | protein peptidyl-prolyl isomerization |
| Cluster-10108.8808 | 1.8867 | 2.1278 | 0.25978 | protein ubiquitination |
| Cluster-10108.12251 | 1.4875 | 1.325 | -0.1438 | protein ubiquitination |
| Cluster-10108.4988 | 1.3282 | 1.372 | 0.06217 | protein ubiquitination |
| Cluster-10108.18425 | 2.72 | 4.1628 | 1.4635 | protein ubiquitination |
| Cluster-10108.17233 | 3.2586 | 3.6767 | 0.4356 | protein ubiquitination |
| Cluster-10108.19987 | 1.0565 | 0.88752 | -0.14991 | protein ubiquitination//apoptotic process//regulation of cytokinesis |
| Cluster-10108.3229 | 1.4855 | 2.7859 | 1.3187 | protein ubiquitination |
| Cluster-10108.6471 | 3.7406 | 4.5105 | 0.78721 | regulation of protein kinase activity |
| Cluster-10108.14848 | 1.4917 | 2.2734 | 0.8017 | protein folding |
| Cluster-10108.7723 | 1.2447 | 1.6174 | 0.39187 | protein prenylation//regulation of mitotic metaphase/anaphase transition |
| Cluster-10108.12806 | 2.7902 | 4.3931 | 1.6247 | protein processing |
| Cluster-10108.16702 | 1.5865 | 3.6848 | 2.1161 | protein processing |
| Cluster-10108.20448 | 2.6293 | 4.9355 | 2.3274 | protein processing |
| Cluster-10108.20079 | 1.5074 | 2.4963 | 1.0085 | protein processing |
| Cluster-10108.18309 | 2.7801 | 5.0279 | 2.2716 | translational initiation//regulation of GTPase activity//negative regulation of translation |
| Cluster-10108.4930 | 1.456 | 1.7367 | 0.29894 | protein glycosylation |
| Cluster-10108.16482 | 1.6513 | 3.1734 | 1.5412 | protein glycosylation |
| Cluster-10108.19557 | 4.1231 | 5.8144 | 1.7098 | aspartate metabolic process//alanine metabolic process//alanyl-tRNA aminoacylation |
| Cluster-10108.13819 | 2.9103 | 10.549 | 7.655 | cellular response to amino acid starvation//negative regulation of TOR signaling//protein processing |
| Cluster-10108.10797 | 1.239 | 1.8399 | 0.6205 | regulation of translational initiation |
| Cluster-10108.24070 | 3.7985 | 5.7109 | 1.9302 | regulation of translational initiation |
| Cluster-10108.9609 | 2.197 | 3.3779 | 1.202 | protein folding |
| Cluster-10108.11705 | 2.1021 | 3.1421 | 1.0613 | protein folding |
| Cluster-10108.12198 | 1.2238 | 2.1082 | 0.90211 | protein folding |
| Cluster-10108.19376 | 1.1768 | 2.1849 | 1.026 | protein N-linked glycosylation |
| Cluster-10108.21277 | 2.1239 | 3.7943 | 1.6892 | protein O-linked mannosylation |
| Cluster-10108.24503 | 1.1928 | 0.71348 | -0.46125 | translational termination//chromosome segregation//protein ubiquitination//lysosome localization//cell division//cristae formation |
| Cluster-10108.5069 | 1.5215 | 2.1422 | 0.64091 | regulation of translation |
| Cluster-10108.11556 | 1.3658 | 2.4504 | 1.1051 | regulation of protein catabolic process |
| Cluster-10108.14175 | 1.0214 | 1.4926 | 0.49128 | translational elongation//regulation of translational elongation//mRNA transport |
| Cluster-10108.18394 | 1.4543 | 2.7058 | 1.2729 | regulation of translational elongation//rRNA processing//translational elongation |
| Cluster-10108.9406 | -2.8563 | -1.5321 | 1.3368 | protein glycosylation |
| Cluster-10108.2568 | -3.1026 | -4.5836 | -1.463 | protein ubiquitination |
| Cluster-10108.9760 | -2.4771 | -3.7624 | -1.2663 | protein ubiquitination |
| Cluster-10108.2937 | -2.8509 | -3.0208 | -0.15301 | protein ubiquitination |
| Cluster-10108.21340 | -1.6716 | -2.2136 | -0.52471 | protein ubiquitination |
| Cluster-10108.13648 | -2.1133 | -2.5951 | -0.46244 | protein ubiquitination |
| Cluster-10108.8738 | -1.3801 | -0.31229 | 1.0855 | protein ubiquitination |
| Cluster-10108.23095 | -2.0021 | -3.4416 | -1.4218 | protein ubiquitination |
| Cluster-10108.1978 | -2.5267 | -2.7738 | -0.22828 | protein ubiquitination |
| Cluster-10108.6979 | -2.2534 | -3.3986 | -1.1293 | protein ubiquitination |
| Cluster-10108.21744 | -2.2402 | -2.1441 | 0.11552 | protein ubiquitination |
| Cluster-10108.936 | -3.3698 | -4.7976 | -1.4128 | protein ubiquitination |
| Cluster-10108.18957 | -2.6169 | -3.9239 | -1.2884 | protein ubiquitination |
| Cluster-10108.19918 | -1.4959 | -1.8393 | -0.32722 | protein ubiquitination |
| Cluster-10108.6820 | -1.5405 | -2.1452 | -0.58677 | protein ubiquitination//carboxylic acid metabolic process |
| Cluster-10108.504 | -2.2051 | -0.21541 | 2.0053 | protein ubiquitination//protein maturation |
| Cluster-10108.26885 | -3.042 | -5.3784 | -2.3159 | protein ubiquitination |
| Cluster-10108.18598 | -1.6722 | -0.31037 | 1.3783 | protein ubiquitination |
| Cluster-10108.25863 | -2.3958 | -3.7354 | -1.3174 | protein ubiquitination |
| Cluster-10108.21925 | -1.7779 | -3.1315 | -1.3355 | protein ubiquitination |
| Cluster-8283.0 | -3.5579 | -3.8121 | -0.2342 | protein ubiquitination |
| Cluster-10108.24537 | -1.0195 | -0.65047 | 0.38633 | protein glycosylation//oxidation-reduction process |
| Cluster-10108.2169 | -5.8752 | -7.6265 | -1.7318 | protein phosphorylation |
| Cluster-10108.19083 | -3.028 | -4.4707 | -1.4252 | ribosome biogenesis//translation//oxidation-reduction process//evasion of host immune response//transport of virus in host, cell to cell |
| Cluster-10108.15325 | -1.0132 | -0.73041 | 0.30044 | protein ubiquitination |
| Cluster-10108.8259 | -1.7563 | -2.5708 | -0.79705 | protein phosphorylation |
| Cluster-10108.22095 | -4.1545 | -5.7415 | -1.5679 | ribosome biogenesis//translation |
| Cluster-10108.2165 | -4.3129 | -5.579 | -1.2495 | protein phosphorylation |
| Cluster-10108.15620 | -1.1388 | -1.2442 | -0.08806 | protein phosphorylation |
| Cluster-10108.3326 | -1.3562 | -1.3759 | -0.00128 | protein ubiquitination |
| Cluster-10108.16627 | -1.2302 | -1.8538 | -0.60635 | protein phosphorylation |
| Cluster-10108.6053 | -2.7159 | -2.3492 | 0.38424 | protein phosphorylation |
| Cluster-10108.18959 | -1.5056 | -2.5174 | -0.99391 | protein phosphorylation |
| Cluster-10108.16456 | -1.1617 | -1.5975 | -0.41853 | protein phosphorylation |
| Cluster-10108.15885 | -1.1563 | -1.7023 | -0.5277 | protein ubiquitination |
| Cluster-10108.2793 | -2.0276 | -2.5694 | -0.52575 | translational initiation |
| Cluster-10108.7964 | -1.0743 | -1.6133 | -0.52263 | protein secretion//signal transduction |
| Cluster-10108.7750 | -1.662 | -1.5741 | 0.10739 | protein phosphorylation//DNA methylation |
| Cluster-10108.16612 | -1.1448 | -1.3998 | -0.2377 | protein phosphorylation//recognition of pollen |
| Cluster-10108.26670 | -4.6839 | -6.2615 | -1.561 | protein phosphorylation |
| Cluster-10108.14218 | -2.6743 | -2.3563 | 0.33468 | protein phosphorylation |
| Cluster-10108.14252 | -1.7997 | -2.1713 | -0.35472 | protein N-linked glycosylation//protein phosphorylation |
| Cluster-10108.20955 | -1.8965 | -3.1165 | -1.2025 | protein phosphorylation |
| Cluster-10108.12485 | -1.2195 | -1.3577 | -0.1209 | protein phosphorylation |
| Cluster-10108.16046 | -1.6343 | -2.2636 | -0.61181 | protein phosphorylation |
| Cluster-10108.5924 | -2.9424 | -3.0443 | -0.08451 | regulation of protein kinase activity//protein phosphorylation |
| Cluster-10108.1944 | -3.1008 | -4.1697 | -1.0513 | protein phosphorylation |
| Cluster-10108.11803 | -1.9296 | -2.8776 | -0.92983 | protein phosphorylation |
| Cluster-10108.26285 | -4.7212 | -6.341 | -1.6016 | protein phosphorylation |
| Cluster-10108.27222 | -3.1153 | -4.9939 | -1.86 | protein phosphorylation |
| Cluster-10108.8749 | -2.8457 | -3.7283 | -0.86802 | protein phosphorylation |
| Cluster-10108.8455 | -3.5755 | -5.0723 | -1.4798 | protein phosphorylation |
| Cluster-10108.6856 | -1.4722 | -1.5809 | -0.09116 | protein phosphorylation |
| Cluster-10108.4443 | -1.7229 | -2.3508 | -0.61091 | protein phosphorylation |
| Cluster-10108.6317 | -2.5036 | -3.6828 | -1.161 | protein phosphorylation//signal transduction |
| Cluster-10108.28062 | -2.1647 | -2.7902 | -0.60818 | protein phosphorylation |
| Cluster-10108.4342 | -1.8614 | -2.9385 | -1.0579 | protein phosphorylation |
| Cluster-10108.19548 | -1.5111 | -0.81291 | 0.71721 | protein phosphorylation |
| Cluster-10108.4842 | -1.3223 | -1.7786 | -0.43826 | protein phosphorylation |
| Cluster-10108.3920 | -1.2798 | -1.3554 | -0.05684 | protein phosphorylation |
| Cluster-10108.8439 | -1.2196 | -1.9266 | -0.68983 | protein phosphorylation |
| Cluster-10108.15578 | -1.1581 | -0.66015 | 0.51715 | protein phosphorylation |
| Cluster-10108.6214 | -1.4162 | -2.2141 | -0.77954 | protein phosphorylation |
| Cluster-10108.4924 | -1.3137 | -0.60593 | 0.72363 | protein phosphorylation |
| Cluster-10108.17496 | -1.2798 | 0.24041 | 1.5368 | protein phosphorylation |
| Cluster-10108.16799 | -1.2869 | -1.9617 | -0.65551 | protein phosphorylation |
| Cluster-10108.140 | -4.9271 | -6.1323 | -1.193 | protein phosphorylation |
| Cluster-10108.16602 | -3.0044 | -4.3158 | -1.2923 | protein phosphorylation |
| Cluster-8263.0 | -2.2253 | -2.2878 | -0.04329 | protein phosphorylation |
| Cluster-10108.25801 | -1.3147 | -2.436 | -1.1034 | protein phosphorylation |
| Cluster-10108.26134 | -1.9163 | -4.4606 | -2.5245 | protein phosphorylation |
| Cluster-10108.23363 | -1.3934 | -2.2674 | -0.85697 | protein phosphorylation |
| Cluster-10108.5838 | -2.4068 | -3.7058 | -1.2784 | protein phosphorylation |
| Cluster-9988.0 | -2.7467 | -2.4425 | 0.32434 | protein phosphorylation |
| Cluster-10108.1584 | -2.4021 | -4.2392 | -1.8172 | protein phosphorylation |
| Cluster-10108.25789 | -1.7485 | -2.492 | -0.72827 | protein phosphorylation |
| Cluster-10108.28125 | -3.4285 | -4.9545 | -1.5035 | protein phosphorylation//recognition of pollen |
| Cluster-10108.11521 | -1.4774 | -2.2367 | -0.73933 | protein maturation//protein ubiquitination |
| Cluster-10108.11733 | -1.8312 | -1.9703 | -0.12178 | protein processing |
| Cluster-10108.4875 | -1.6157 | -2.2926 | -0.65791 | regulation of translational initiation//translational initiation |
| Cluster-10108.19343 | -2.1722 | -3.934 | -1.7437 | regulation of translational initiation//translational initiation |
| Cluster-10108.16957 | -1.124 | -0.75858 | 0.38267 | regulation of translational initiation//positive regulation of viral transcription//oxidation-reduction process//translational initiation |
| Cluster-10108.3687 | -1.1883 | -0.29967 | 0.90544 | regulation of translational initiation |
| Cluster-10108.18448 | -1.9667 | -2.1538 | -0.17072 | regulation of protein kinase activity |
| Cluster-10108.6272 | -2.9551 | -4.5067 | -1.5337 | regulation of protein kinase activity |
| Cluster-10108.18007 | -1.1139 | -1.5562 | -0.42413 | regulation of protein kinase activity |
| Cluster-10108.9539 | -1.2861 | -0.96253 | 0.34003 | protein peptidyl-prolyl isomerization |
| Cluster-10108.16522 | -1.0255 | -0.75315 | 0.28907 | protein peptidyl-prolyl isomerization |
| Cluster-10108.8231 | -1.8722 | -2.7879 | -0.89815 | protein phosphorylation//protein ubiquitination |
| Cluster-10108.16583 | -1.4853 | -1.8926 | -0.3897 | protein phosphorylation//signal transduction//fusion of virus membrane with host plasma membrane |
| Cluster-10108.19075 | -1.5947 | -1.155 | 0.45666 | protein phosphorylation//oxidation-reduction process//mitochondrial electron transport, NADH to ubiquinone |
| Cluster-10108.18501 | -1.4861 | -2.2991 | -0.7947 | protein phosphorylation//serine family amino acid metabolic process//phosphorylation//carbohydrate metabolic process |
| Cluster-10108.6941 | -1.5053 | -1.7432 | -0.22052 | protein phosphorylation |
| Cluster-10108.4917 | -2.5254 | -3.1419 | -0.59843 | protein phosphorylation//serine family amino acid metabolic process//phosphorylation//recognition of pollen |
| Cluster-10108.22697 | -1.564 | -0.72809 | 0.85271 | protein phosphorylation//ribosome biogenesis//translation |
| Cluster-10108.16413 | -1.0052 | -0.93163 | 0.091459 | protein phosphorylation//oxidation-reduction process |
| Cluster-10108.18873 | -1.669 | -2.8 | -1.1137 | protein phosphorylation//ribosome biogenesis//translation |
| Cluster-10108.4208 | -3.2684 | -5.2252 | -1.9384 | protein phosphorylation |
| Cluster-10108.22113 | -1.2353 | -1.7363 | -0.48219 | protein phosphorylation |
| Cluster-10108.20474 | -1.1363 | -2.3463 | -1.1913 | protein phosphorylation//serine family amino acid metabolic process//phosphorylation |
| Cluster-10108.15440 | -1.8404 | -2.5649 | -0.70688 | ribosome biogenesis//translation |
| Cluster-10108.14189 | -1.3607 | -1.6446 | -0.26722 | ribosome biogenesis//translation |
| Cluster-10108.9742 | -2.0225 | -1.9708 | 0.067767 | ribosome biogenesis//translation//ATP synthesis coupled proton transport |
| Cluster-10108.11998 | -1.6424 | -1.7478 | -0.08865 | ribosome biogenesis//translation |
| Cluster-10108.19287 | -2.1266 | -3.0952 | -0.95089 | ribosome biogenesis//translation//phospholipid transport |
| Cluster-10108.20025 | -1.1242 | -1.4925 | -0.35086 | ribosome biogenesis//translation//oxidation-reduction process |
| Cluster-10108.19810 | -1.3587 | -0.86611 | 0.50888 | translation//ribosome biogenesis |
| Cluster-10108.15629 | -1.1932 | -0.57567 | 0.63394 | ribosome biogenesis//translation |
| Cluster-10108.17329 | -1.2224 | -1.8954 | -0.65558 | ribosome biogenesis//translation |
| Cluster-10108.7876 | -1.1327 | -1.5597 | -0.4093 | ribosome biogenesis//translation |
| Cluster-10108.26466 | -7.6568 | -8.5226 | -0.85062 | translation//ribosome biogenesis//magnesium ion transport |
| Cluster-10108.28969 | -2.2364 | -4.0581 | -1.8053 | translational initiation//regulation of translational initiation//ribosome biogenesis//translation |
| Cluster-10108.27174 | -8.5043 | -9.8431 | -1.3294 | ribosome biogenesis//translation |
| Cluster-10108.2778 | -2.293 | -4.1052 | -1.7954 | ribosome biogenesis//translation |
| Cluster-10108.20034 | -2.4256 | -2.5244 | -0.08081 | ribosome biogenesis//translation |
| Cluster-10108.1071 | -7.4319 | -8.1482 | -0.70397 | translation//ribosome biogenesis |
| Cluster-10108.298 | -6.1144 | -8.2641 | -2.1441 | ribosome biogenesis//translation |
| Cluster-10108.12812 | -2.146 | -2.9171 | -0.75306 | ribosome biogenesis |
| Cluster-10108.9576 | -1.5993 | -1.6999 | -0.08358 | protein folding//cell motility |
| Cluster-10108.16995 | -1.6427 | -1.8596 | -0.19946 | glutamate metabolic process//L-phenylalanine biosynthetic process//tyrosine biosynthetic process//2-oxoglutarate metabolic process//alkaloid biosynthetic process//tryptophan biosynthetic process//aromatic amino acid family catabolic process |
| Cluster-10108.17867 | -6.8493 | -8.9382 | -2.0691 | aspartate metabolic process//alanine metabolic process//asparagine biosynthetic process |
| Cluster-10108.19364 | -1.6014 | -1.3519 | 0.26761 | cellular response to amino acid starvation//negative regulation of TOR signaling |
| Cluster-10108.18823 | -1.7298 | -2.4716 | -0.72399 | protein catabolic process |
| Cluster-10108.9473 | -1.6957 | -2.755 | -1.0421 | protein catabolic process |
| Cluster-10108.6097 | -1.2207 | -1.9444 | -0.70633 | protein prenylation |
| Cluster-10108.13219 | -1.5466 | -2.5039 | -0.93982 | regulation of protein catabolic process |
| Cluster-10108.13824 | -1.1457 | -0.55572 | 0.60571 | translation//ribosome biogenesis//oxidation-reduction process |
| Cluster-10108.16466 | -1.1355 | -1.3022 | -0.14875 | regulation of translational termination//translational termination |
| Cluster-10108.9515 | -1.2591 | -1.4131 | -0.13688 | protein N-linked glycosylation |
| Cluster-10108.399 | -1.9209 | -1.9179 | 0.018509 | protein N-linked glycosylation via asparagine |
| Cluster-10108.11409 | -1.4326 | -0.98364 | 0.46519 | protein complex oligomerization//histone acetylation |
| Cluster-10108.6304 | -2.1887 | -2.4216 | -0.21412 | arginine metabolic process//peptidyl-proline hydroxylation to 4-hydroxy-L-proline//proline metabolic process//oxidation-reduction process |
| Cluster-10108.15216 | -1.5049 | -2.5207 | -0.99635 | protein stabilization//photosynthesis |
| Cluster-10108.21626 | -1.0636 | -2.3045 | -1.2245 | cellular amino acid metabolic process//biosynthetic process//transsulfuration |
| Cluster-10108.10463 | -0.94362 | -1.3036 | -0.34149 | protein phosphorylation |
| Cluster-10108.16305 | -0.6599 | -1.2987 | -0.62143 | protein ubiquitination |
| Cluster-10108.8196 | 0.98969 | 2.2329 | 1.2632 | protein phosphorylation |
| Cluster-10108.2122 | -9.6663 | -6.5318 | 3.1508 | regulation of translational initiation//translational initiation |
| Cluster-10108.20844 | 1.3356 | 2.6856 | 1.366 | protein phosphorylation |
| Cluster-10108.24853 | 1.2545 | 2.1751 | 0.94113 | regulation of translation//conjugation |
| Cluster-10108.4003 | -0.66821 | -1.3051 | -0.61982 | cellular response to oxidative stress |
| Cluster-10108.10195 | -0.89462 | -1.3798 | -0.46791 | protein ubiquitination |
| Cluster-10108.20972 | 0.9577 | 1.8435 | 0.90403 | protein secretion//vesicle-mediated transport//intracellular protein transport |
| Cluster-10108.14170 | 0.17382 | 2.3159 | 2.1582 | translational elongation//peptide biosynthetic process//regulation of translational elongation |
| Cluster-10108.18761 | 0.040403 | 1.4173 | 1.3937 | protein phosphorylation |
| Cluster-10108.2690 | 0.079232 | -1.8694 | -1.9282 | protein methylation//signal transduction//lysine catabolic process |
| Cluster-10108.4649 | -0.72816 | -1.4473 | -0.70197 | protein folding |
| Cluster-10108.8834 | -0.53311 | -1.4811 | -0.92951 | protein autoprocessing//protein phosphorylation |
| Cluster-10108.15622 | 0.74937 | 1.2297 | 0.49912 | protein phosphorylation//centromere complex assembly |
| Cluster-10108.4032 | 1.1608 | 2.0465 | 0.90674 | regulation of translation |
| Cluster-10108.13587 | 0.21069 | 2.3575 | 2.1629 | protein phosphorylation |
| Cluster-10108.6122 | 1.093 | 1.9907 | 0.91722 | regulation of translation |
| Cluster-10108.8281 | 1.0725 | 2.2916 | 1.2395 | regulation of translation |
| Cluster-10108.16083 | -0.30359 | -1.3178 | -0.99534 | protein trimerization |
| Cluster-10108.4062 | 1.4435 | 1.7295 | 0.30797 | regulation of translation |
| Cluster-10108.2716 | 0.98579 | 2.1646 | 1.1978 | regulation of translation |
| Cluster-10108.11163 | 1.5113 | 2.3777 | 0.8879 | regulation of translation |
| Cluster-10108.17485 | -0.84819 | -1.5479 | -0.68202 | protein ubiquitination |
| Cluster-10108.14725 | 0.55805 | 1.2958 | 0.75498 | cellular amino acid biosynthetic process//methionine biosynthetic process |
| Cluster-10108.16410 | -0.72355 | -1.2089 | -0.46806 | protein ubiquitination |
| Cluster-10108.15554 | -0.74105 | -1.1657 | -0.40698 | regulation of translation//ATP synthesis coupled proton transport//protein phosphorylation |
| Cluster-10108.19238 | 1.3071 | 2.1408 | 0.8548 | regulation of translation//transcription, DNA-templated |
| Cluster-10108.2756 | 2.144 | 4.5984 | 2.4832 | protein ubiquitination//ubiquinone biosynthetic process |
| Cluster-10108.17287 | -0.80943 | -1.1287 | -0.29999 | protein ubiquitination |
| Cluster-6366.0 | -0.25018 | -4.1314 | -3.8422 | protein ubiquitination |
| Cluster-10108.21253 | -0.84019 | -1.3212 | -0.46398 | protein ubiquitination |
| Cluster-10108.4944 | 1.1871 | 1.6669 | 0.49888 | protein phosphorylation |
| Cluster-10108.12243 | -0.52286 | -1.2072 | -0.6676 | protein phosphorylation |
| Cluster-10108.1609 | -8.699 | -6.5965 | 2.1174 | protein phosphorylation |
| Cluster-10108.5343 | -0.98186 | -1.6654 | -0.66555 | protein phosphorylation |
| Cluster-10108.19913 | -0.50148 | -1.0901 | -0.57013 | protein phosphorylation |
| Cluster-10108.20350 | -0.31972 | -1.3798 | -1.041 | protein phosphorylation |
| Cluster-10108.21412 | -1.2226 | -2.7251 | -1.4851 | protein phosphorylation//recognition of pollen |
| Cluster-10108.12475 | -0.16934 | -1.4664 | -1.2792 | protein phosphorylation |
| Cluster-10108.20857 | -0.31226 | 1.6163 | 1.9473 | protein phosphorylation |
| Cluster-10108.22370 | 0.29147 | 2.368 | 2.094 | protein phosphorylation//cell adhesion involved in single-species biofilm formation |
| Cluster-10108.7399 | -0.61315 | -1.4399 | -0.8082 | protein phosphorylation//regulation of transcription, DNA-templated |
| Cluster-10108.29609 | -3.5407 | -7.2364 | -3.6854 | protein phosphorylation |
| Cluster-10108.15394 | 0.74491 | 2.8912 | 2.1657 | protein phosphorylation//regulation of transcription, DNA-templated |
| Cluster-10108.20085 | -0.6733 | -1.5849 | -0.8933 | protein phosphorylation |
| Cluster-10108.16923 | 0.97246 | 1.7494 | 0.7954 | protein phosphorylation |
| Cluster-10108.29203 | -1.9057 | -3.3928 | -1.4677 | protein phosphorylation |
| Cluster-10108.3714 | 0.9848 | 2.3759 | 1.4098 | protein phosphorylation |
| Cluster-10108.8116 | -0.92203 | -1.682 | -0.74186 | protein phosphorylation |
| Cluster-10108.10514 | -0.76346 | 0.3142 | 1.0955 | protein phosphorylation |
| Cluster-10108.18715 | -1.5299 | -1.9431 | -0.39505 | protein phosphorylation |
| Cluster-10108.806 | -1.8298 | -3.5165 | -1.6708 | protein phosphorylation |
| Cluster-10108.8411 | 0.89996 | 1.6552 | 0.77337 | protein phosphorylation |
| Cluster-10108.14915 | 0.72002 | 1.6244 | 0.92223 | protein phosphorylation |
| Cluster-10108.14086 | -0.66093 | -1.1262 | -0.44674 | protein phosphorylation//endoplasmic reticulum to Golgi vesicle-mediated transport//intracellular protein transport |
| Cluster-10108.672 | -1.7517 | -3.0445 | -1.2742 | protein phosphorylation |
| Cluster-8928.0 | 1.0373 | 1.6683 | 0.64945 | protein phosphorylation |
| Cluster-10108.9553 | -0.49893 | -1.1987 | -0.68192 | protein phosphorylation |
| Cluster-10108.153 | -0.62625 | -3.1706 | -2.5237 | protein phosphorylation |
| Cluster-10108.7405 | 0.063361 | -1.1608 | -1.2046 | protein phosphorylation |
| Cluster-10108.1302 | -7.7945 | -5.8213 | 1.9896 | protein phosphorylation |
| Cluster-10108.13564 | 0.84872 | 2.2663 | 1.4336 | protein phosphorylation |
| Cluster-10108.5927 | -0.46565 | -1.2007 | -0.71691 | protein phosphorylation |
| Cluster-10108.3942 | -1.3048 | -2.7425 | -1.419 | protein phosphorylation |
| Cluster-10108.10080 | 0.87488 | 1.082 | 0.22487 | protein phosphorylation |
| Cluster-10108.4393 | -0.613 | -1.7169 | -1.0869 | protein phosphorylation//signal transduction |
| Cluster-10108.16775 | -0.76366 | -1.0515 | -0.27042 | protein phosphorylation//DNA replication |
| Cluster-10108.11311 | 0.58015 | 1.2305 | 0.66911 | protein phosphorylation |
| Cluster-7850.0 | -1.6485 | -2.4727 | -0.80528 | protein phosphorylation |
| Cluster-10108.20930 | 1.9786 | 4.4739 | 2.5027 | protein phosphorylation |
| Cluster-10108.30046 | -0.88598 | -2.5376 | -1.6306 | protein phosphorylation |
| Cluster-10108.5966 | -2.1537 | -2.3067 | -0.13595 | protein phosphorylation |
| Cluster-10108.3102 | -0.67468 | -1.504 | -0.81065 | protein phosphorylation |
| Cluster-10108.6261 | -0.1865 | 1.0249 | 1.2275 | protein phosphorylation |
| Cluster-10108.4353 | 0.55311 | 1.367 | 0.83275 | protein phosphorylation |
| Cluster-10108.29467 | -5.3357 | -7.3845 | -2.0333 | protein phosphorylation |
| Cluster-10108.11826 | 0.81571 | 1.2675 | 0.47037 | protein phosphorylation |
| Cluster-10108.15766 | -0.59438 | -1.0172 | -0.40454 | protein phosphorylation |
| Cluster-10108.16389 | -0.8517 | -1.5703 | -0.70147 | protein phosphorylation |
| Cluster-10108.11859 | 0.20068 | 1.391 | 1.2051 | protein phosphorylation |
| Cluster-10108.25326 | -4.3001 | -5.2974 | -0.9726 | protein phosphorylation |
| Cluster-10108.5401 | -0.76156 | -1.8431 | -1.064 | protein phosphorylation |
| Cluster-10108.17510 | -0.95931 | -1.783 | -0.8055 | protein phosphorylation |
| Cluster-10108.9400 | -0.55424 | -1.1387 | -0.5663 | protein phosphorylation//oxidation-reduction process |
| Cluster-10108.4450 | 1.1661 | 1.4879 | 0.33785 | protein phosphorylation |
| Cluster-10108.14852 | 0.71325 | 1.3323 | 0.63675 | protein phosphorylation//viral process |
| Cluster-10108.9300 | -0.80364 | -1.2407 | -0.41891 | protein phosphorylation |
| Cluster-10108.21060 | -0.98456 | -1.0719 | -0.06907 | protein phosphorylation |
| Cluster-10108.11030 | -0.38446 | -1.0275 | -0.6242 | protein phosphorylation |
| Cluster-10108.10037 | -0.59555 | -1.1657 | -0.55268 | protein phosphorylation |
| Cluster-10108.20804 | -1.6833 | -2.8443 | -1.1434 | protein phosphorylation |
| Cluster-10108.17342 | 0.89822 | 1.5568 | 0.67731 | protein phosphorylation |
| Cluster-10108.1418 | -8.2989 | -5.6519 | 2.6672 | protein phosphorylation |
| Cluster-10108.10884 | -1.1267 | -2.2316 | -1.0858 | protein phosphorylation |
| Cluster-10108.4975 | -0.79707 | -1.6131 | -0.79713 | protein phosphorylation |
| Cluster-10108.22631 | -0.3225 | -1.2778 | -0.93628 | protein phosphorylation |
| Cluster-10108.7226 | 0.34962 | 1.0247 | 0.69273 | protein phosphorylation |
| Cluster-10108.10583 | -0.67672 | -1.1118 | -0.41722 | protein phosphorylation |
| Cluster-10108.17164 | -0.76555 | -1.3347 | -0.55168 | protein phosphorylation |
| Cluster-10108.28807 | -2.6371 | -3.2938 | -0.64692 | protein phosphorylation |
| Cluster-10108.9130 | -0.427 | -1.116 | -0.67013 | protein phosphorylation |
| Cluster-10108.23228 | 1.0649 | 1.8851 | 0.83985 | protein phosphorylation |
| Cluster-10108.12145 | -0.98454 | -1.4919 | -0.48933 | protein phosphorylation |
| Cluster-10108.18552 | -0.19 | 1.1226 | 1.3305 | protein phosphorylation//phosphorylation//serine family amino acid metabolic process |
| Cluster-10108.13066 | 0.18321 | 2.1262 | 1.9613 | protein phosphorylation |
| Cluster-10108.1970 | -24.707 | -6.4512 | 5.0749 | protein phosphorylation |
| Cluster-10108.14068 | -0.32376 | -1.058 | -0.71741 | protein peptidyl-prolyl isomerization |
| Cluster-10108.7229 | -0.77147 | -1.5728 | -0.78387 | protein peptidyl-prolyl isomerization |
| Cluster-10108.9087 | 0.58421 | 1.0101 | 0.44466 | protein peptidyl-prolyl isomerization |
| Cluster-6068.0 | -2.9903 | -3.4721 | -0.46386 | protein peptidyl-prolyl isomerization |
| Cluster-10108.12123 | 0.95384 | 1.0439 | 0.11016 | protein peptidyl-prolyl isomerization |
| Cluster-10108.11849 | 0.77656 | 1.5359 | 0.77851 | translation//ribosome biogenesis//oxidation-reduction process |
| Cluster-10108.8056 | 0.98532 | 1.6646 | 0.69755 | translation//serine family amino acid metabolic process//phosphorylation//glycosaminoglycan catabolic process//glycerolipid metabolic process//galactose metabolic process//glycosphingolipid metabolic process |
| Cluster-10108.2099 | -23.455 | -7.1081 | 3.1248 | translation//ribosome biogenesis |
| Cluster-10108.9695 | 0.93664 | 1.6354 | 0.71864 | translation//ribosome biogenesis |
| Cluster-10108.13151 | 0.65829 | 1.3709 | 0.73214 | translation//ribosome biogenesis |
| Cluster-9912.0 | -2.41 | -2.9409 | -0.50747 | ribosome biogenesis//translation |
| Cluster-10108.29377 | -6.2433 | -8.2858 | -2.0381 | ribosome biogenesis//translation |
| Cluster-10108.13664 | 0.8179 | 2.0596 | 1.2618 | ribosome biogenesis//translation |
| Cluster-10108.18750 | 0.99321 | 1.8234 | 0.84904 | ribosome biogenesis//translation |
| Cluster-10108.1877 | -22.875 | -7.7983 | 2.8361 | ribosome biogenesis//translation |
| Cluster-10108.13681 | 0.93675 | 1.6586 | 0.74107 | ribosome biogenesis//translation |
| Cluster-10108.6845 | -0.31596 | -1.0537 | -0.71785 | ribosome biogenesis//translation |
| Cluster-10108.11971 | 0.93498 | 1.7063 | 0.79141 | ribosome biogenesis//translation |
| Cluster-10108.15807 | 0.85374 | 4.8892 | 4.0578 | ribosome biogenesis//translation |
| Cluster-10108.21238 | 0.9827 | 3.4424 | 2.4797 | ribosome biogenesis |
| Cluster-10108.25689 | -22.849 | -6.6366 | 3.8093 | translation//ribosome biogenesis |
| Cluster-10108.1999 | -24.83 | -8.9784 | 2.6705 | translation//ribosome biogenesis |
| Cluster-10108.356 | -5.9755 | -8.8588 | -2.8819 | ribosome biogenesis//translation |
| Cluster-10108.9358 | 0.84667 | 1.3086 | 0.48181 | translation//ribosome biogenesis |
| Cluster-10108.2127 | -25.103 | -9.818 | 2.1155 | translation//ribosome biogenesis |
| Cluster-10108.10391 | 0.84266 | 1.4138 | 0.59089 | translation//ribosome biogenesis |
| Cluster-10108.1874 | -10.812 | -9.7809 | 1.0471 | ribosome biogenesis//translation |
| Cluster-10108.5887 | -0.47342 | -1.0714 | -0.57878 | ribosome biogenesis//translation |
| Cluster-10108.2010 | -24.47 | -8.1934 | 3.0885 | translation//ribosome biogenesis |
| Cluster-10108.7801 | -0.4832 | -1.1355 | -0.63453 | protein complex oligomerization//protein phosphorylation//ribosome biogenesis//phosphorylation//serine family amino acid metabolic process//translation |
| Cluster-10108.12482 | 0.98345 | 1.6788 | 0.71492 | ribosome biogenesis//translation |
| Cluster-10108.14017 | 0.67122 | 1.2266 | 0.57407 | ribosome biogenesis//translation |
| Cluster-10108.13366 | 0.88329 | 1.6039 | 0.7393 | translation//ribosome biogenesis//G protein-coupled receptor signaling pathway, coupled to cyclic nucleotide second messenger |
| Cluster-10108.13668 | 0.91251 | 2.2393 | 1.3467 | ribosome biogenesis//translation |
| Cluster-10108.12684 | 0.84724 | 1.9023 | 1.0742 | translation//ribosome biogenesis |
| Cluster-10108.1770 | -9.2069 | -8.3164 | 0.90732 | ribosome biogenesis//translation |
| Cluster-10108.14108 | 0.94529 | 1.812 | 0.88575 | ribosome biogenesis//translation |
| Cluster-10108.18413 | 0.82822 | 1.318 | 0.51 | translation//ribosome biogenesis |
| Cluster-10108.16846 | 0.43102 | 1.206 | 0.79461 | translation//ribosome biogenesis |
| Cluster-10108.2090 | -10.942 | -8.9443 | 2.0159 | ribosome biogenesis//translation |
| Cluster-10108.13557 | 0.66087 | 1.3157 | 0.67445 | ribosome biogenesis//translation |
| Cluster-10108.10928 | 0.76788 | 1.487 | 0.73926 | translation//ribosome biogenesis |
| Cluster-10108.18375 | 0.41736 | 1.1453 | 0.74652 | translation//ribosome biogenesis |
| Cluster-10108.14136 | 1.005 | 1.7975 | 0.81213 | translation//ribosome biogenesis |
| Cluster-10108.4268 | 0.95117 | 1.4991 | 0.56761 | translation//ribosome biogenesis//signal transduction |
| Cluster-10108.11744 | 0.63007 | 1.2317 | 0.61997 | translation//ribosome biogenesis |
| Cluster-10108.16006 | 0.56888 | 1.1615 | 0.60983 | ribosome biogenesis//translation |
| Cluster-10108.8941 | 0.86843 | 1.3592 | 0.50949 | translation//ribosome biogenesis |
| Cluster-10108.44 | -1.2953 | -5.2465 | -3.9335 | ribosome biogenesis//translation |
| Cluster-10108.13211 | 0.91934 | 1.7413 | 0.84121 | translation//ribosome biogenesis//modification-dependent protein catabolic process//proteasomal protein catabolic process |
| Cluster-10108.6662 | -0.49846 | -1.0345 | -0.51758 | translation//ribosome biogenesis |
| Cluster-10108.9364 | -0.94536 | -1.6435 | -0.67998 | ribosome biogenesis//translation |
| Cluster-10108.11632 | -0.62017 | 0.57205 | 1.2103 | translation//ribosome biogenesis//autophagosome assembly |
| Cluster-10108.13723 | 0.96276 | 1.7959 | 0.85283 | translation//ribosome biogenesis |
| Cluster-10108.18202 | 0.92161 | 1.28 | 0.37666 | ribosome biogenesis//intracellular protein transport//translation//vesicle-mediated transport |
| Cluster-10108.14256 | 0.7518 | 1.4414 | 0.70851 | ribosome biogenesis//translation//regulation of transcription, DNA-templated |
| Cluster-10108.13487 | 0.92675 | 1.7544 | 0.84733 | translation//ribosome biogenesis |
| Cluster-10108.12347 | 1.098 | 2.4481 | 1.3707 | ribosome biogenesis//translation |
| Cluster-10108.13463 | 0.68224 | 1.3437 | 0.68072 | translation//ribosome biogenesis |
| Cluster-10108.8821 | 0.93797 | 1.5956 | 0.67705 | ribosome biogenesis//translation |
| Cluster-10108.17052 | 0.73867 | 1.4091 | 0.68952 | translation//ribosome biogenesis |
| Cluster-10108.15114 | 0.80022 | 1.2448 | 0.46374 | ribosome biogenesis//translation |
| Cluster-10108.12492 | 0.96566 | 1.3624 | 0.41664 | translation//ribosome biogenesis |
| Cluster-10108.18264 | 0.78287 | 1.6972 | 0.93373 | ribosome biogenesis//translation |
| Cluster-10108.20104 | 0.97997 | 1.5591 | 0.59783 | ribosome biogenesis//translation |
| Cluster-10108.13598 | 0.81637 | 1.3217 | 0.52481 | ribosome biogenesis//translation//heme transport |
| Cluster-10108.12729 | 0.91389 | 1.1464 | 0.25236 | translation//ribosome biogenesis |
| Cluster-10108.10376 | 0.4631 | 1.2211 | 0.77367 | protein glycosylation |
| Cluster-10108.16115 | 0.57595 | 1.3958 | 0.83742 | protein glycosylation |
| Cluster-10108.28306 | -6.6367 | -6.4595 | 0.19979 | protein glycosylation |
| Cluster-10108.22864 | -1.6695 | -4.0847 | -2.3984 | protein glycosylation//oxidation-reduction process//ubiquinone biosynthetic process//mitochondrial electron transport, NADH to ubiquinone//sodium ion transport |
| Cluster-10108.8425 | 0.6365 | 1.2758 | 0.65871 | ribosome biogenesis//translation |
| Cluster-10108.15355 | 0.18132 | 1.3766 | 1.2135 | protein glycosylation |
| Cluster-10108.15498 | -0.82634 | -2.0315 | -1.1874 | protein glycosylation |
| Cluster-10108.1169 | -22.128 | -5.7231 | 3.131 | protein glycosylation |
| Cluster-10108.7490 | -0.7944 | -1.1025 | -0.2904 | aromatic amino acid family biosynthetic process//L-phenylalanine biosynthetic process//tyrosine biosynthetic process//oxidation-reduction process//porphyrin-containing compound biosynthetic process//tryptophan biosynthetic process |
| Cluster-10108.19523 | -0.07948 | -1.5028 | -1.4048 | protein maturation |
| Cluster-10108.23677 | 2.5938 | 6.9369 | 4.3556 | protein ubiquitination |
| Cluster-10108.6147 | -0.54386 | -1.098 | -0.53426 | protein folding |
| Cluster-10108.14727 | 0.64625 | 1.4566 | 0.82819 | protein folding |
| Cluster-10108.1916 | -9.0786 | -6.0534 | 2.9402 | protein folding |
| Cluster-10108.16525 | 0.86989 | 1.2905 | 0.43993 | protein folding |
| Cluster-10108.11304 | -0.88701 | -1.4426 | -0.53839 | protein folding |
| Cluster-10108.19773 | 0.03135 | 2.7474 | 2.7308 | protein ubiquitination//methionine biosynthetic process |
| Cluster-10108.22346 | 0.53666 | 2.3132 | 1.7954 | protein ubiquitination |
| Cluster-10108.1802 | -1.4705 | -2.2892 | -0.80145 | protein ubiquitination |
| Cluster-10108.15231 | 0.59079 | 1.0052 | 0.43247 | protein ubiquitination |
| Cluster-10108.11833 | 0.94239 | 1.4191 | 0.49521 | protein ubiquitination//viral process//proteolysis//symbiotic process |
| Cluster-10108.5782 | -0.74716 | -1.1273 | -0.36259 | protein ubiquitination |
| Cluster-10108.6233 | 0.75863 | 1.6235 | 0.8825 | protein ubiquitination |
| Cluster-10108.11653 | 0.71399 | 1.096 | 0.39965 | protein ubiquitination |
| Cluster-10108.13802 | -0.8489 | -1.398 | -0.5313 | protein ubiquitination//protein maturation |
| Cluster-10108.5021 | -0.62634 | -1.2183 | -0.57337 | protein ubiquitination |
| Cluster-10108.22487 | -0.37736 | -1.1895 | -0.79362 | protein ubiquitination |
| Cluster-10108.19952 | -0.81702 | -1.5499 | -0.71506 | protein ubiquitination |
| Cluster-10108.3021 | -1.213 | -2.4753 | -1.2431 | protein ubiquitination |
| Cluster-10108.18708 | 0.64678 | 1.4129 | 0.78451 | protein ubiquitination |
| Cluster-10108.7738 | 0.77931 | 2.4564 | 1.6955 | protein ubiquitination |
| Cluster-10108.11403 | 0.6086 | 3.9773 | 3.3838 | protein ubiquitination//oxidation-reduction process |
| Cluster-10108.19915 | -0.73187 | -1.2954 | -0.5449 | protein ubiquitination |
| Cluster-10108.8584 | 0.82578 | 1.4194 | 0.61214 | translational initiation//mRNA splicing, via spliceosome//DNA damage checkpoint//regulation of translational initiation |
| Cluster-10108.11504 | 0.78098 | 1.208 | 0.44494 | translational initiation//transmembrane transport//regulation of translational initiation//pyrimidine nucleotide-sugar transmembrane transport |
| Cluster-10108.2059 | -22.906 | -5.7086 | 3.9521 | translational initiation//regulation of translational initiation |
| Cluster-10108.17144 | -0.45844 | -1.1382 | -0.66178 | translational initiation//mitigation of host defenses by virus//viral transcription//purine nucleobase metabolic process//transcription, DNA-templated//regulation of translational initiation//pyrimidine nucleobase metabolic process |
| Cluster-10108.14705 | 0.91083 | 1.3141 | 0.42173 | protein glycosylation//interstrand cross-link repair |
| Cluster-10108.18374 | 0.90307 | 1.9581 | 1.0741 | protein glycosylation |
| Cluster-10108.26299 | -8.6499 | -5.3755 | 3.3009 | protein glycosylation |
| Cluster-10108.9474 | 0.41789 | 1.9892 | 1.5879 | protein glycosylation//base-excision repair |
| Cluster-10108.17642 | 0.66799 | 2.6074 | 1.9568 | protein glycosylation |
| Cluster-10108.1921 | -10.069 | -5.8313 | 4.1703 | protein glycosylation |
| Cluster-10108.8928 | 0.5103 | 2.0154 | 1.5237 | protein glycosylation//base-excision repair |
| Cluster-10108.10408 | 0.79018 | 1.1507 | 0.37883 | protein glycosylation |
| Cluster-10108.12376 | 0.52739 | 1.0041 | 0.49572 | arginine metabolic process//tRNA aminoacylation for protein translation//proline metabolic process//prolyl-tRNA aminoacylation |
| Cluster-10108.14766 | 0.80176 | 1.3358 | 0.55187 | arginine metabolic process//pyruvate metabolic process//sodium ion transport//proline metabolic process//sodium ion export across plasma membrane |
| Cluster-10108.19646 | -0.64422 | -1.3163 | -0.65571 | arginine metabolic process//pyruvate metabolic process//sodium ion transport//G protein-coupled receptor signaling pathway, coupled to cyclic nucleotide second messenger//proline metabolic process//DNA replication//sodium ion export across plasma membrane |
| Cluster-10108.7657 | 0.76746 | 1.4252 | 0.67602 | arginine metabolic process//pyruvate metabolic process//sodium ion transport//proline metabolic process//sodium ion export across plasma membrane//proteolysis//asexual sporulation |
| Cluster-10108.19095 | -0.84999 | -1.2682 | -0.40003 | arginine metabolic process//proteolysis |
| Cluster-10108.8574 | 0.64322 | 1.0126 | 0.3876 | arginine metabolic process//DNA replication//tRNA threonylcarbamoyladenosine modification//DNA repair//DNA recombination |
| Cluster-10108.7476 | -0.96621 | -1.2473 | -0.2636 | arginine metabolic process//pyruvate metabolic process//sodium ion transport//proline metabolic process//protein targeting//protein phosphorylation//intracellular protein transport//sodium ion export across plasma membrane |
| Cluster-10108.22036 | -0.62114 | -1.6457 | -1.0064 | arginine metabolic process//peptidyl-proline hydroxylation to 4-hydroxy-L-proline//DNA repair//proline metabolic process//oxidation-reduction process//DNA recombination |
| Cluster-10108.8216 | 0.46468 | 1.0836 | 0.63652 | arginine metabolic process//sodium ion transport//pyruvate metabolic process//regulation of store-operated calcium entry//proline metabolic process//G protein-coupled receptor signaling pathway//sodium ion export across plasma membrane |
| Cluster-10108.15835 | -0.85115 | -1.2834 | -0.41438 | arginine metabolic process//ATP-dependent chromatin remodeling//pyruvate metabolic process//sodium ion transport//kinetochore assembly//transcription by RNA polymerase I//tRNA splicing, via endonucleolytic cleavage and ligation//sodium ion export across plasma membrane//pathogenesis//protein transport//regulation of translational initiation//DNA repair//proline metabolic process//quorum sensing |
| Cluster-10108.1247 | -2.4523 | -4.3799 | -1.9095 | arginine metabolic process//pyruvate metabolic process//sodium ion transport//proline metabolic process//intracellular protein transport//sodium ion export across plasma membrane |
| Cluster-10108.275 | -4.5034 | -5.5216 | -1.0098 | cellular amino acid metabolic process//cell redox homeostasis//signal transduction//obsolete electron transport |
| Cluster-10108.12548 | 0.41222 | 1.5398 | 1.146 | cellular amino acid metabolic process |
| Cluster-10108.3140 | -0.95782 | -1.9095 | -0.93408 | protein N-linked glycosylation |
| Cluster-10108.15115 | 0.8983 | 1.2301 | 0.35025 | protein N-linked glycosylation via asparagine |
| Cluster-10108.9080 | 0.2514 | 1.2475 | 1.016 | protein N-linked glycosylation |
| Cluster-10108.14493 | 0.95993 | 1.4778 | 0.53653 | protein N-linked glycosylation via asparagine |
| Cluster-10108.11082 | 0.78812 | 1.1406 | 0.37137 | protein N-linked glycosylation//DNA repair |
| Cluster-10108.25738 | -6.4899 | -4.1427 | 2.3676 | translation//ribosome biogenesis |
| Cluster-10108.8670 | 0.91949 | 1.4897 | 0.58823 | translation//ribosome biogenesis |
| Cluster-10108.1549 | -3.065 | -3.4977 | -0.41275 | translation//purine nucleobase metabolic process//ribosome biogenesis//pyrimidine nucleobase metabolic process//transcription, DNA-templated//kinetochore assembly |
| Cluster-10108.6417 | 0.65341 | 1.0472 | 0.41315 | translation//serine family amino acid metabolic process//ribosome biogenesis//phosphorylation |
| Cluster-10108.6146 | 1.4221 | 2.0757 | 0.6721 | translation//regulation of pH//ribosome biogenesis//mitotic sister chromatid cohesion//sodium ion transport |
| Cluster-10108.15868 | 0.20033 | 1.871 | 1.6876 | translation//ribosome biogenesis//regulation of GTPase activity |
| Cluster-10108.2222 | -11.653 | -9.4592 | 2.2111 | ribosome biogenesis |
| Cluster-10108.15227 | -0.52445 | -1.2849 | -0.7424 | ribosome biogenesis//translation//intracellular protein transport//DNA replication |
| Cluster-10108.11034 | 0.23222 | 1.0378 | 0.82464 | ribosome biogenesis//translation//regulation of transcription, DNA-templated |
| Cluster-10108.13561 | -0.7137 | -1.0381 | -0.30682 | ribosome biogenesis//pseudouridine synthesis//obsolete acyl-carrier-protein biosynthetic process//lipid metabolic process |
| Cluster-10108.2027 | -22.983 | -6.5341 | 3.2121 | ribosome biogenesis//translation |
| Cluster-10108.3103 | 1.5645 | 2.2332 | 0.69039 | regulation of translation |
| Cluster-10108.20627 | 1.2821 | 2.7424 | 1.4794 | regulation of translation//Group I intron splicing |
| Cluster-10108.3034 | 1.6508 | 2.3332 | 0.69871 | regulation of translation |
| Cluster-10108.20291 | 1.7646 | 2.8041 | 1.0599 | regulation of translation//regulation of apoptotic process |
| Cluster-10108.21020 | 0.53017 | 1.367 | 0.85532 | regulation of translation |
| Cluster-10108.7545 | 1.03 | 2.4831 | 1.4705 | regulation of translation |
| Cluster-10108.15540 | 0.69488 | 1.302 | 0.62511 | regulation of translation//regulation of apoptotic process |
| Cluster-10108.22845 | 0.87854 | 1.4987 | 0.63785 | regulation of translation |
| Cluster-10108.9752 | 0.65388 | 1.6899 | 1.0559 | regulation of translation |
| Cluster-10108.4797 | 1.1739 | 1.9188 | 0.76741 | regulation of translation |
| Cluster-10108.10306 | -0.52808 | -1.0081 | -0.46168 | protein-containing complex assembly |
| Cluster-10108.29953 | -1.337 | -1.7873 | -0.43026 | regulation of translational initiation//translational initiation |
| Cluster-10108.10182 | -0.61402 | -1.2035 | -0.5716 | regulation of translational initiation |
| Cluster-10108.16325 | 0.79869 | 1.2053 | 0.42618 | regulation of translational initiation |
| Cluster-10108.10816 | 0.96098 | 1.3359 | 0.39382 | regulation of translational initiation |
| Cluster-508.0 | -3.238 | -6.5891 | -3.3506 | regulation of translational initiation//maturation of SSU-rRNA from tricistronic rRNA transcript (SSU-rRNA, 5.8S rRNA, LSU-rRNA)//translational initiation |
| Cluster-10108.21063 | 0.82451 | 1.3195 | 0.51517 | regulation of translational initiation//positive regulation of mitochondrial translation |
| Cluster-10108.8033 | -0.62862 | -1.2333 | -0.58674 | cellular amino acid metabolic process//protein folding |
| Cluster-10108.18566 | 0.83457 | 1.1072 | 0.29301 | translational elongation//peptide biosynthetic process//regulation of translational elongation |
| Cluster-10108.8005 | -0.78749 | -1.1388 | -0.33363 | protein-phycocyanobilin linkage |
| Cluster-10108.21376 | -0.82498 | -1.8029 | -0.95882 | asparagine metabolic process |
| Cluster-10108.4301 | 0.91879 | 2.186 | 1.2877 | asparagine metabolic process |
| Cluster-10108.7897 | 0.37795 | 1.8067 | 1.4485 | aspartate metabolic process//alanine metabolic process//asparagine biosynthetic process |
| Cluster-10108.6008 | 0.73868 | 1.212 | 0.49238 | protein neddylation |
| Cluster-10108.8815 | 0.83162 | 1.2275 | 0.41421 | protein deubiquitination |
| Cluster-10108.9418 | 0.83005 | 1.0801 | 0.26902 | protein deubiquitination |
| Cluster-7749.0 | 3.4424 | 4.8346 | 1.393 | protein deubiquitination//proteolysis//smoothened signaling pathway//ubiquitin-dependent protein catabolic process |
| Cluster-10108.10327 | -0.92808 | -1.3071 | -0.36089 | protein dephosphorylation//tyrosine metabolic process//ATP-dependent chromatin remodeling |
| Cluster-10108.3044 | 2.7055 | 2.8083 | 0.12262 | protein processing//regulation of store-operated calcium entry//histone modification//transcription elongation from RNA polymerase II promoter |
| Cluster-10108.17850 | 0.87631 | 1.1525 | 0.29459 | protein processing |
| Cluster-10108.2983 | 0.4676 | 4.1951 | 3.7435 | regulation of protein kinase activity |
| Cluster-10108.11798 | -0.73164 | -1.4871 | -0.73776 | protein methylation//lysine catabolic process |
| Cluster-10108.11236 | 1.329 | 3.3722 | 2.0646 | protein methylation//C-terminal protein methylation |
| Cluster-10108.3595 | 0.84028 | 1.7599 | 0.93926 | protein methylation//C-terminal protein methylation |
| Cluster-10108.20163 | -0.95512 | -2.1859 | -1.2111 | protein methylation//oxidation-reduction process//lysine catabolic process |
| Cluster-10108.19265 | 0.90763 | 1.24 | 0.35093 | protein maturation |
| Cluster-10108.15573 | 0.93673 | 1.5093 | 0.59215 | translational elongation//regulation of translational elongation |
| Cluster-10108.13417 | 0.6643 | 1.0656 | 0.42003 | translational elongation//regulation of translational elongation |
| Cluster-10108.4693 | -0.88824 | -1.8852 | -0.97672 | protein dephosphorylation//tyrosine metabolic process//dephosphorylation |
| Cluster-10108.12035 | -0.50983 | -1.0269 | -0.49907 | protein complex oligomerization |
| Cluster-10108.13401 | 0.99339 | 2.7685 | 1.7926 | protein retention in ER lumen |
| Cluster-2487.0 | -1.2529 | -5.2982 | -4.0254 | protein trimerization//negative regulation of signal transduction |
| Cluster-10108.9344 | -0.88575 | -1.2954 | -0.39212 | protein homooligomerization |

# Table S8 Genes Related to Polysaccharide, Cell Wall and Cytoskeleton Metabolism

| gene_id | T1VST3 | T1VST4 | T3VST4 | BP Description |
| --- | --- | --- | --- | --- |
| Cluster-10108.13783 | 4.0505 | 2.6969 |  | chitin catabolic process//cell wall macromolecule catabolic process |
| Cluster-10108.15665 | 1.0816 | 1.9753 |  | chitin catabolic process//cell wall macromolecule catabolic process |
| Cluster-10108.14388 | 1.4128 | 1.7269 |  | -- |
| Cluster-10108.13804 | 1.4604 | 2.0262 |  | -- |
| Cluster-10108.13560 | 1.6101 | 2.556 |  | -- |
| Cluster-10108.10862 | 1.5663 | 1.6528 |  | cell wall macromolecule catabolic process//chitin catabolic process |
| Cluster-10108.8739 | 2.997 | 4.0683 |  | microtubule-based process//microtubule-based movement |
| Cluster-10108.9116 | 2.5647 | 2.2713 |  | microtubule-based process//microtubule-based movement |
| Cluster-10108.8299 | 2.4526 | 3.5017 |  | microtubule-based movement//microtubule-based process |
| Cluster-10108.15870 | 3.3739 | 5.5886 | 2.2381 | microtubule-based movement//microtubule-based process//attachment of spindle microtubules to kinetochore |
| Cluster-10108.9536 | 3.2122 | 5.1265 |  | microtubule-based movement//microtubule-based process |
| Cluster-10108.20021 | 3.4067 | 3.273 |  | microtubule-based process//microtubule-based movement//tRNA threonylcarbamoyladenosine modification//DNA repair//DNA recombination//SRP-dependent cotranslational protein targeting to membrane//viral genome replication |
| Cluster-10108.9889 | 2.6788 | 3.792 |  | plant-type cell wall organization//DNA replication//DNA replication initiation//cell wall biogenesis |
| Cluster-10108.9187 | 1.1647 | 1.9669 |  | electron transport chain//cytoskeleton organization |
| Cluster-10108.9511 | 2.7901 | 3.1901 |  | negative regulation of phosphoprotein phosphatase activity//telomere maintenance |
| Cluster-10108.14627 | 2.2907 | 3.0232 |  | microtubule-based process//microtubule-based movement |
| Cluster-10108.9992 | 1.3414 | 1.0825 |  | microtubule-based process//microtubule-based movement |
| Cluster-10108.7414 | 3.2053 | 5.234 |  | microtubule-based process//microtubule-based movement |
| Cluster-10108.11824 | 3.5514 | 5.4631 |  | microtubule-based process//microtubule-based movement |
| Cluster-10108.20440 | 3.2907 | 3.8339 |  | mitotic cell cycle |
| Cluster-10108.12410 | 1.1922 | 2.6207 | 1.4477 | mitotic cell cycle |
| Cluster-10108.12127 | 1.7473 | 1.612 |  | -- |
| Cluster-10108.16636 | 2.7945 | 3.2765 |  | mitotic spindle assembly checkpoint//cristae formation//response to antibiotic//attachment of mitotic spindle microtubules to kinetochore |
| Cluster-10108.9327 | 4.2687 | 4.8918 |  | transformation of host cell by virus |
| Cluster-10108.9499 | 2.9704 | 2.4865 |  | transformation of host cell by virus |
| Cluster-10108.14622 | 4.3324 | 8.6644 | 4.3538 | cell wall biogenesis//plant-type cell wall organization |
| Cluster-10108.6307 | 3.1529 | 5.0039 |  | -- |
| Cluster-10108.7930 | 1.5608 | 1.7412 |  | -- |
| Cluster-10108.13989 | 3.5176 | 4.9121 |  | microtubule-based movement//microtubule-based process//telomere maintenance//DNA repair |
| Cluster-10108.3669 | 4.7417 | 9.4092 |  | bacterial-type flagellum-dependent cell motility//pyrimidine nucleobase metabolic process//signal transduction//DNA integration//transcription, DNA-templated//purine nucleobase metabolic process |
| Cluster-10108.4989 | 1.5297 | 2.4429 |  | pathogenesis//viral process//quorum sensing |
| Cluster-10108.9920 | 1.7128 | 1.9523 |  | -- |
| Cluster-10108.12074 | 1.4126 | 1.7699 |  | -- |
| Cluster-10108.18198 | 1.2753 | 1.826 |  | nucleocytoplasmic transport |
| Cluster-10108.6082 | 1.6879 | 2.2141 |  | -- |
| Cluster-10108.12218 | 2.1246 | 2.8169 |  | plasma membrane fusion involved in cytogamy |
| Cluster-10108.7015 | 1.5878 | 1.6468 |  | cellular metabolic process//protein trimerization//signal transduction//platelet activation//spindle pole body duplication//protein polymerization |
| Cluster-10108.9367 | 3.3544 | 4.1595 |  | attachment of spindle microtubules to kinetochore |
| Cluster-10108.6951 | 3.3272 | 4.1159 |  | -- |
| Cluster-10108.16001 | 3.4665 | 8.4435 |  | -- |
| Cluster-10108.10516 | 3.518 | 5.0434 |  | microtubule-based movement//axon guidance//microtubule-based process |
| Cluster-10108.12765 | 3.7609 | 5.6381 |  | microtubule-based process//microtubule-based movement//cristae formation |
| Cluster-10108.14207 | 3.6138 | 6.109 |  | microtubule-based movement//microtubule-based process |
| Cluster-10108.14636 | 2.9975 | 4.6204 |  | microtubule-based movement//microtubule-based process |
| Cluster-10108.8090 | 1.2844 | 2.5693 | 1.3043 | microtubule-based movement//cell septum assembly//DNA replication//septin ring assembly//nitrogen compound metabolic process//transport//FtsZ-dependent cytokinesis//cell adhesion//microtubule-based process//transmembrane transport//protein import into nucleus//Golgi organization//metal ion transport |
| Cluster-10108.12417 | 1.7151 | 3.6174 | 1.9215 | microtubule-based movement//microtubule-based process |
| Cluster-10108.16211 | 1.6756 | 3.1851 | 1.5293 | microtubule-based movement//microtubule-based process//DNA replication |
| Cluster-10108.17281 | 1.1206 | 1.3406 |  | microtubule-based process |
| Cluster-10108.17733 | 1.011 | 1.6405 |  | microtubule-based movement//microtubule-based process//obsolete electron transport//oxidative phosphorylation//protein import into nucleus//oxidation-reduction process |
| Cluster-10108.18503 | 1.0192 | 1.2889 |  | microtubule-based process |
| Cluster-10108.13559 | 3.4309 | 5.0285 |  | -- |
| Cluster-10108.11386 | 2.1551 | 2.45 |  | cortical actin cytoskeleton organization//intracellular protein transport//gamma-tubulin complex localization |
| Cluster-10108.6643 | 3.4948 | 4.4515 |  | transport of virus in host, cell to cell//DNA replication//cellulose biosynthetic process//sucrose metabolic process//starch metabolic process//DNA repair//UDP-glucose metabolic process//DNA recombination |
| Cluster-10108.7346 | 2.1603 | 4.5746 |  | alpha-tubulin acetylation//obsolete acyl-carrier-protein biosynthetic process |
| Cluster-10108.11299 | 1.2469 | 1.2734 |  | FtsZ-dependent cytokinesis//regulation of transcription, DNA-templated//cell septum assembly//DNA replication//microtubule-based movement//microtubule-based process |
| Cluster-10108.18764 | 2.5089 | 2.9298 |  | viral process//microtubule-based movement//microtubule-based process//regulation of transcription, DNA-templated//rRNA processing |
| Cluster-10108.8260 | 1.7832 | 3.7207 | 1.9536 | transcription, DNA-templated//starch metabolic process//cell wall modification//pyrimidine nucleobase metabolic process//sucrose metabolic process//purine nucleobase metabolic process |
| Cluster-10108.10571 | 3.0808 | 4.9296 |  | cell wall modification//sucrose metabolic process//starch metabolic process |
| Cluster-10108.13545 | 3.8824 | 7.2859 | 3.4289 | cell wall biogenesis//plant-type cell wall organization//feeding behavior//neuropeptide signaling pathway |
| Cluster-10108.22423 | 2.6381 | 2.5579 |  | cell wall macromolecule biosynthetic process |
| Cluster-10108.4635 | 2.2656 | 5.0487 |  | cell wall modification//sucrose metabolic process//starch metabolic process |
| Cluster-10108.4427 | 3.8911 | 6.2948 |  | -- |
| Cluster-10108.7770 | 1.7301 | 2.0886 |  | protein phosphorylation//recognition of pollen//phosphorylation//serine family amino acid metabolic process |
| Cluster-10108.19183 | 2.7125 | 5.665 |  | serine family amino acid metabolic process//phosphorylation |
| Cluster-10108.18066 | 2.8571 | 4.6934 | 1.8583 | cellular metabolic process//spindle pole body duplication//microtubule-based process//microtubule-based movement//cell septum assembly//FtsZ-dependent cytokinesis |
| Cluster-10108.14592 | 3.5943 | 6.0157 |  | pyrimidine nucleobase metabolic process//purine nucleobase metabolic process//microtubule-based movement//microtubule-based process//regulation of transcription, DNA-templated//transcription, DNA-templated |
| Cluster-10108.9114 | 3.8321 | 5.9982 |  | regulation of autophagy//microtubule-based movement//microtubule-based process//potassium ion transport |
| Cluster-10108.14236 | 1.2815 | 1.6955 |  | ATP biosynthetic process//bacteriocin immunity |
| Cluster-10108.18784 | 2.3295 | 3.832 |  | ATP synthesis coupled proton transport |
| Cluster-10108.3695 | 3.0726 | 4.1064 |  | ATP synthesis coupled proton transport//regulation of DNA-templated transcription, termination//transcriptional attenuation by ribosome |
| Cluster-10108.21774 | 1.8682 | 3.2465 |  | ATP synthesis coupled proton transport |
| Cluster-10108.4768 | 2.351 | 4.8243 |  | ATP synthesis coupled proton transport |
| Cluster-10108.12414 | 1.0606 | 1.7696 |  | ATP synthesis coupled proton transport |
| Cluster-10108.19479 | 1.6963 | 2.5496 |  | ATP synthesis coupled proton transport |
| Cluster-10108.19754 | 1.2034 | 3.4257 | 2.2402 | ATP synthesis coupled proton transport//sucrose metabolic process//cell wall modification//starch metabolic process |
| Cluster-10108.18977 | 1.4416 | 2.2191 |  | ATP synthesis coupled proton transport |
| Cluster-10108.3610 | 1.2448 | 1.4945 |  | telomere maintenance//G protein-coupled receptor signaling pathway//mannose metabolic process//fructose metabolic process |
| Cluster-10108.9740 | 3.3007 | 6.4769 | 3.1971 | -- |
| Cluster-10108.9697 | 3.0238 | 4.3217 |  | septin ring assembly |
| Cluster-10108.15260 | 1.2639 | 2.8111 | 1.5665 | septin ring assembly |
| Cluster-10108.19636 | 1.1697 | 1.2304 |  | -- |
| Cluster-10108.10382 | 2.1826 | 3.7838 | 1.6216 | cell motility//Golgi organization |
| Cluster-10108.15194 | 1.9674 | 3.5247 |  | bacterial-type flagellum-dependent cell motility |
| Cluster-10108.19395 | 2.1686 | 3.6417 |  | intracellular protein transport//vesicle-mediated transport |
| Cluster-10108.5399 | 3.1611 | 3.2987 |  | pathogenesis//metal ion transport |
| Cluster-10108.10796 | 3.6572 | 5.4372 |  | oxidation-reduction process//lipoprotein metabolic process//lipid transport//protein complex oligomerization//ribosome biogenesis//translation//bacterial-type flagellum-dependent cell motility//lysosome localization//DNA catabolic process//histidine biosynthetic process |
| Cluster-10108.17745 | 1.183 |  |  | -- |
| Cluster-10108.25100 | 1.4767 |  |  | pathogenesis |
| Cluster-10108.10305 | 1.4461 | 2.0887 |  | intracellular protein transport//vesicle-mediated transport |
| Cluster-10108.23184 | 3.1737 |  |  | -- |
| Cluster-10108.10536 | 1.3479 | 1.8712 |  | FtsZ-dependent cytokinesis//intra-Golgi vesicle-mediated transport//DNA replication//cell septum assembly//viral life cycle//cell motility//protein folding |
| Cluster-10108.5234 | 4.0453 | 7.1404 |  | actin cytoskeleton organization//regulation of transcription, DNA-templated |
| Cluster-10108.13693 | 1.4914 | 2.8483 | 1.3767 | starch metabolic process//sucrose metabolic process//cell wall modification |
| Cluster-10108.10250 | 1.3322 | 1.7776 |  | translation//ribosome biogenesis//nitrogen compound metabolic process//chemotaxis//cortical actin cytoskeleton organization |
| Cluster-10108.12473 | 1.149 | 2.1002 |  | actin filament organization//embryonic development via the syncytial blastoderm//endocytosis//receptor signaling pathway via JAK-STAT//defense response to virus |
| Cluster-10108.17877 | -2.6532 | -3.1439 |  | cellulose biosynthetic process//UDP-glucose metabolic process//starch metabolic process//obsolete acyl-carrier-protein biosynthetic process//sucrose metabolic process//fatty acid biosynthetic process//plasma membrane fusion involved in cytogamy |
| Cluster-10108.7839 | -4.4066 | -5.7013 | -1.2773 | UDP-glucose metabolic process//cellulose biosynthetic process//sucrose metabolic process//starch metabolic process |
| Cluster-10108.17085 | -3.0766 | -4.0318 |  | potassium ion transport//cytolysis//sucrose metabolic process//defense response to Gram-negative bacterium//cellulose biosynthetic process//UDP-glucose metabolic process//starch metabolic process |
| Cluster-10108.17109 | -2.0564 | -2.6622 |  | starch metabolic process//sucrose metabolic process//cellulose biosynthetic process//UDP-glucose metabolic process |
| Cluster-10108.10931 | -1.7034 | -2.0497 |  | sucrose metabolic process//starch metabolic process//UDP-glucose metabolic process//cellulose biosynthetic process |
| Cluster-10108.18023 | -1.3386 | -2.2964 |  | defense response to Gram-negative bacterium//starch metabolic process//UDP-glucose metabolic process//cellulose biosynthetic process//cytolysis//sucrose metabolic process |
| Cluster-10108.9077 | -1.9141 | -3.7525 | -1.82 | multicellular organism development//starch metabolic process//UDP-glucose metabolic process//cellulose biosynthetic process//sucrose metabolic process |
| Cluster-10108.18485 | -1.5155 | -2.3383 |  | sucrose metabolic process//starch metabolic process//UDP-glucose metabolic process//cellulose biosynthetic process//sporulation resulting in formation of a cellular spore |
| Cluster-10108.7859 | -1.0885 | -1.7352 |  | UDP-glucose metabolic process//cellulose biosynthetic process//sucrose metabolic process//starch metabolic process |
| Cluster-10108.20858 | -2.4193 | -2.2384 |  | sucrose metabolic process//viral capsid assembly//starch metabolic process//UDP-glucose metabolic process//cellulose biosynthetic process |
| Cluster-10108.19200 | -2.2055 | -2.8031 |  | -- |
| Cluster-10108.23073 | -1.9833 | -2.2239 |  | defense response//regulation of vasoconstriction//chitin catabolic process//cell wall macromolecule catabolic process |
| Cluster-10108.21924 | -4.0533 | -5.1276 |  | regulation of RNA metabolic process//UDP-glucose metabolic process//cellulose biosynthetic process//starch metabolic process//RNA processing//sucrose metabolic process |
| Cluster-10108.396 | -2.1863 | -1.9455 |  | regulation of RNA metabolic process |
| Cluster-10108.22585 | -6.7357 | -8.7226 | -1.9657 | microtubule-based process |
| Cluster-10108.8719 | -1.2581 | -1.5034 |  | microtubule-based process |
| Cluster-10108.3068 | -5.5072 | -6.8705 |  | microtubule-based process |
| Cluster-10108.4316 | -3.9058 | -5.0936 | -1.1698 | UDP-glucose metabolic process//cellulose biosynthetic process//mitochondrial electron transport, cytochrome c to oxygen//starch metabolic process//sucrose metabolic process |
| Cluster-10108.18253 | -1.5957 | -1.6752 |  | Wnt signaling pathway |
| Cluster-10108.10251 | -1.2117 | -1.9802 |  | regulation of transcription, DNA-templated |
| Cluster-10108.16376 | -2.3179 | -3.2428 |  | viral DNA genome replication//(1->3)-beta-D-glucan biosynthetic process//obsolete cytoskeletal anchoring at plasma membrane//sucrose metabolic process//proteolysis//starch metabolic process |
| Cluster-10108.18071 | -1.5179 |  |  | -- |
| Cluster-10108.21621 | -3.3371 | -4.3006 |  | plant-type cell wall organization//regulation of transcription, DNA-templated//cell wall biogenesis |
| Cluster-10108.4125 | -2.4235 | -2.5932 |  | post-chaperonin tubulin folding pathway//tubulin complex assembly |
| Cluster-10108.4657 | -3.2091 | -4.4768 | -1.251 | -- |
| Cluster-10108.4208 | -3.2684 | -5.2252 | -1.9384 | protein phosphorylation |
| Cluster-10108.6891 | -2.7201 | -3.2845 |  | -- |
| Cluster-10108.22113 | -1.2353 | -1.7363 |  | protein phosphorylation |
| Cluster-10108.14521 | -1.607 | -2.1212 |  | mannose metabolic process//SRP-dependent cotranslational protein targeting to membrane//fructose metabolic process |
| Cluster-10108.16729 | -4.1554 | -6.0555 | -1.881 | cell wall macromolecule catabolic process//chitin catabolic process |
| Cluster-10108.18275 | -3.0888 | -3.5852 |  | chitin catabolic process//cell wall macromolecule catabolic process |
| Cluster-10108.13182 | -1.5474 | -2.6265 | -1.0617 | cell wall macromolecule catabolic process//chitin catabolic process |
| Cluster-10108.4873 | -1.2848 | -2.406 |  | chitin catabolic process//cell wall macromolecule catabolic process |
| Cluster-10108.11887 | -1.0136 |  |  | mRNA processing//microtubule-based movement//regulation of RNA metabolic process//microtubule-based process//defense response |
| Cluster-10108.706 | -2.1399 | -2.154 |  | regulation of RNA metabolic process |
| Cluster-10108.25986 | -1.6297 |  |  | regulation of RNA metabolic process//cell wall biogenesis//tRNA processing//plant-type cell wall organization |
| Cluster-10108.10834 | -2.059 | -3.9524 | -1.8766 | -- |
| Cluster-10108.11292 | -1.5292 |  | 1.2137 | -- |
| Cluster-10108.9347 | -1.8429 | -2.4926 |  | mRNA splice site selection//polysaccharide metabolic process//obsolete electron transport |
| Cluster-10108.7311 | -1.0534 | -1.483 |  | fungal-type cell wall organization |
| Cluster-10108.7821 | -1.0153 |  |  | fungal-type cell wall organization |
| Cluster-10108.5474 | -1.1772 | -1.8554 |  | cytoskeleton organization |
| Cluster-10108.15800 | -1.0485 | -1.6492 |  | -- |
| Cluster-10108.21785 | -1.4727 | -2.1063 |  | -- |
| Cluster-10108.4539 | -1.5874 |  |  | growth//sucrose metabolic process//cell wall modification//cell population proliferation//starch metabolic process//signal transduction |
| Cluster-10108.2696 | -2.2146 | -3.9782 |  | -- |
| Cluster-10108.25296 | -1.9492 | -2.0344 |  | cell adhesion//tubulin complex assembly//post-chaperonin tubulin folding pathway |
| Cluster-10108.7434 | | 2.0103 |  | -- |
| Cluster-10108.2113 | | -5.8922 |  | bacterial-type flagellum organization |
| Cluster-10108.9952 | | 1.4975 |  | bacterial-type flagellum organization |
| Cluster-10108.7416 | |  | 1.2278 | bacterial-type flagellum-dependent cell motility |
| Cluster-10108.22312 | | 1.1952 |  | bacterial-type flagellum-dependent cell motility |
| Cluster-10108.3594 | | 2.08 |  | regulation of cell cycle |
| Cluster-10108.5554 | | 1.4308 |  | regulation of cell growth |
| Cluster-10108.8794 | | 1.9514 | 1.4681 | regulation of cell cycle//transcription initiation from RNA polymerase II promoter |
| Cluster-10108.14691 | | 1.9197 | 1.5013 | regulation of cell growth//lysosomal transport//D-alanine metabolic process//peptidoglycan biosynthetic process |
| Cluster-5627.0 | | -5.1631 |  | regulation of cell growth |
| Cluster-10108.32 | | -4.6233 |  | regulation of cell cycle |
| Cluster-10108.16192 | | 1.1862 |  | transformation of host cell by virus//pathogenesis |
| Cluster-10108.21312 | | -1.22 |  | -- |
| Cluster-10108.13279 | | 1.1614 |  | intracellular protein transport//purine nucleobase metabolic process//vesicle-mediated transport//viral genome replication//protein targeting |
| Cluster-10108.19105 | | 1.6559 |  | -- |
| Cluster-10108.8802 | | 1.221 |  | cell adhesion//DNA integration//signal transduction |
| Cluster-10108.18724 | | -1.0204 |  | FtsZ-dependent cytokinesis//protein phosphorylation//cell septum assembly//protein ubiquitination |
| Cluster-9664.0 | | -2.8315 |  | archaeal or bacterial-type flagellum-dependent cell motility |
| Cluster-10108.14544 | | -1.5594 | -1.075 | lipid metabolic process |
| Cluster-10108.23185 | | -1.5108 |  | -- |
| Cluster-10108.4646 | | 1.2703 |  | maturation of SSU-rRNA from tricistronic rRNA transcript (SSU-rRNA, 5.8S rRNA, LSU-rRNA)//bacterial-type flagellum-dependent cell motility//protein trimerization//mRNA processing |
| Cluster-10108.10063 | | -1.1281 |  | potassium ion transport//cation transport |
| Cluster-10108.10776 | | 1.9816 | 1.3201 | intracellular protein transport//vesicle-mediated transport |
| Cluster-10108.13091 | | 1.4093 |  | pathogenesis |
| Cluster-10108.14782 | | 1.1928 |  | serine family amino acid metabolic process//intracellular protein transport//phosphorylation//neuronal signal transduction//bacterial-type flagellum-dependent cell motility//protein phosphorylation |
| Cluster-10108.12089 | | | 1.0215 | -- |
| Cluster-10108.10809 | | 1.1713 |  | transmembrane transport//DNA-templated transcription, initiation |
| Cluster-10108.17740 | | 2.6749 |  | pathogenesis |
| Cluster-10108.9737 | | -1.2451 |  | potassium ion transport//positive regulation of apoptotic process |
| Cluster-10108.2027 | | -6.5341 |  | ribosome biogenesis//translation |
| Cluster-10108.10304 | | 1.3239 |  | -- |
| Cluster-10108.20595 | -3.25 | -3.825 |  | protein transport//chromosome organization |
| Cluster-10108.16536 | -1.7596 | -1.8398 |  | -- |
| Cluster-10108.20947 | -3.1419 | -4.3034 | -1.1443 | -- |
| Cluster-10108.20768 | -3.3948 | -5.1479 | -1.7358 | virion assembly//proteolysis |
| Cluster-10108.28960 | -4.2878 | -7.8718 | -3.5664 | -- |
| Cluster-10108.25719 | -2.1999 | -2.153 |  | regulation of transcription, DNA-templated |
| Cluster-10108.19880 | -1.3172 |  |  | -- |
| Cluster-10108.6565 | -3.145 | -2.7918 |  | -- |
| Cluster-10108.15761 | -1.3206 | -1.7473 |  | -- |
| Cluster-10108.14473 | | 1.9945 | 1.1835 | sucrose metabolic process//pathogenesis//quorum sensing//starch metabolic process//cell wall modification |
| Cluster-10108.5813 | |  | 1.2287 | sucrose metabolic process//starch metabolic process//carbohydrate metabolic process |
| Cluster-8721.0 | | -5.4486 |  | sucrose metabolic process//starch metabolic process//carbohydrate metabolic process |
| Cluster-10108.13654 | | -1.0327 |  | sucrose metabolic process//starch metabolic process//polysaccharide catabolic process |
| Cluster-10108.4247 | |  | 1.3633 | sucrose metabolic process//signal transduction//starch metabolic process//UDP-glucose metabolic process//cellulose biosynthetic process//archaeal or bacterial-type flagellum-dependent cell motility |
| Cluster-10108.21236 | | 1.7396 |  | sucrose metabolic process//starch metabolic process//carbohydrate metabolic process |
| Cluster-10108.10836 | | 4.1216 |  | sucrose metabolic process//starch metabolic process//carbohydrate metabolic process |
| Cluster-10108.22676 | | 1.0425 |  | regulation of actin filament polymerization//Arp2/3 complex-mediated actin nucleation |
| Cluster-323.0 | | -6.5891 |  | starch metabolic process//sucrose metabolic process//glycolytic process//pentose-phosphate shunt//gluconeogenesis |
| Cluster-10108.10519 | | 1.7864 | 1.3486 | starch metabolic process//sucrose metabolic process//cellulose biosynthetic process//UDP-glucose metabolic process |
| Cluster-10108.10639 | | | 1.0789 | starch metabolic process//DNA repair//(1->3)-beta-D-glucan biosynthetic process//sucrose metabolic process |
| Cluster-10108.9142 | | -1.265 |  | starch metabolic process//galactose metabolic process//streptomycin biosynthetic process//protein phosphorylation//glucose 6-phosphate metabolic process//phosphatidylinositol metabolic process//sucrose metabolic process//gluconeogenesis//glycolytic process |
| Cluster-10108.20800 | | 1.9385 |  | starch metabolic process//glycogen biosynthetic process//potassium ion transport//potassium ion transmembrane transport//sucrose metabolic process |
| Cluster-10108.7563 | | 1.1457 |  | starch metabolic process//sucrose metabolic process//polysaccharide catabolic process |
| Cluster-10108.11745 | | -1.0797 |  | starch metabolic process//sucrose metabolic process//carbohydrate metabolic process |
| Cluster-10108.21335 | | 3.7996 |  | starch metabolic process//sucrose metabolic process//carbohydrate metabolic process |
| Cluster-10108.5326 | | 1.7278 |  | starch metabolic process//sucrose metabolic process//cell wall modification |
| Cluster-10108.21173 | | 2.2719 | 1.5016 | -- |
| Cluster-10108.13798 | | 2.2096 | 1.5297 | folic acid biosynthetic process//mitochondrial electron transport, NADH to ubiquinone//methanogenesis//cell motility//oxidation-reduction process |
| Cluster-10108.8530 | | 1.4462 |  | -- |
| Cluster-10108.16518 | | 2.0255 |  | -- |
| Cluster-10108.2093 | | -4.6936 |  | -- |
| Cluster-10108.29726 | | -4.6162 |  | cytoskeleton organization |
| Cluster-10108.11414 | | -1.2331 |  | cytoskeleton organization |
| Cluster-10108.22293 | | 1.3533 |  | DNA integration//cytoskeleton organization |
| Cluster-10108.13620 | | 1.11 |  | microtubule-based process//obsolete acyl-carrier-protein biosynthetic process//microtubule-based movement |
| Cluster-10108.13872 | | 1.5378 | 1.075 | cellulose microfibril organization |
| Cluster-10108.5290 | | 1.6979 |  | cellulose microfibril organization |
| Cluster-10108.14511 | | 1.7682 |  | cellulose microfibril organization//intracellular signal transduction |
| Cluster-10108.22499 | | -3.6679 |  | cellulose microfibril organization//carbohydrate metabolic process |
| Cluster-10108.17100 | | 1.999 |  | cellulose microfibril organization//viral process |
| Cluster-10108.16830 | | 1.5064 | 1.3061 | cellulose microfibril organization |
| Cluster-10108.10199 | | 1.6781 |  | positive regulation of Golgi to plasma membrane protein transport |
| Cluster-10108.3507 | | 1.9385 |  | positive regulation of Golgi to plasma membrane protein transport//DNA repair//DNA recombination |
| Cluster-10108.13362 | | 1.2942 |  | microtubule-based movement//microtubule-based process//carbohydrate metabolic process |
| Cluster-10108.12606 | | 1.6646 |  | microtubule-based process//microtubule-based movement//ATP synthesis coupled proton transport |
| Cluster-10108.7985 | | 1.1679 |  | microtubule-based process//microtubule-based movement |
| Cluster-10108.22225 | | 1.8863 |  | microtubule-based process//proton transmembrane transport |
| Cluster-10108.2168 | | -5.8996 |  | galactose metabolic process//pentose-phosphate shunt//fructose metabolic process//gluconeogenesis//mannose metabolic process//glycolytic process |
| Cluster-10108.10357 | | -1.1795 |  | galactose metabolic process//glycosphingolipid metabolic process//glycerolipid metabolic process//glycosaminoglycan catabolic process//carbohydrate metabolic process |
| Cluster-10108.17695 | | 1.2132 |  | galactose metabolic process//lipopolysaccharide biosynthetic process//nucleotide-sugar metabolic process//nucleotide metabolic process |
| Cluster-10108.8484 | | 1.4642 |  | galactose metabolic process//pentose-phosphate shunt//fructose metabolic process//gluconeogenesis//mannose metabolic process//glycolytic process |
| Cluster-10108.13624 | | 2.9065 | 2.1036 | galactose metabolic process//starch metabolic process//protein phosphorylation//sucrose metabolic process//transmembrane transport |
| Cluster-10108.10383 | | -1.3793 |  | epidermis development |
| Cluster-10108.13818 | | 1.3417 |  | epidermis development |
| Cluster-10108.19676 | | 3.0839 | 2.4957 | cell wall modification//sucrose metabolic process//starch metabolic process |
| Cluster-10108.14205 | | 1.7509 |  | cell wall modification//sucrose metabolic process//starch metabolic process |
| Cluster-10002.0 | | 6.3956 |  | cell wall modification//starch metabolic process//sucrose metabolic process |
| Cluster-10108.29415 | | -5.5978 |  | cell wall modification//starch metabolic process//sucrose metabolic process |
| Cluster-10108.843 | | -5.8815 | -3.5409 | cell wall modification//sucrose metabolic process//starch metabolic process |
| Cluster-10108.3234 | | 2.4821 |  | cell wall modification//starch metabolic process//sucrose metabolic process |
| Cluster-10108.15760 | | 1.055 |  | vesicle docking involved in exocytosis//gas vesicle organization |
| Cluster-10108.18792 | | 1.5528 | 1.8965 | -- |
| Cluster-9151.0 | | -4.9412 |  | glycosphingolipid metabolic process |
| Cluster-5275.0 | | -5.8487 |  | oxidation-reduction process |
| Cluster-10108.17481 | | 2.7404 |  | -- |
| Cluster-10108.15204 | | 3.0681 |  | -- |
| Cluster-10108.12156 | | 3.1058 | 2.3549 | -- |
| Cluster-10108.29371 | | -2.5725 |  | -- |
| Cluster-10108.4716 | | 8.7981 | 6.9805 | modulation by virus of host cellular process |
| Cluster-10108.19009 | | -6.5014 |  | Arp2/3 complex-mediated actin nucleation//regulation of actin filament polymerization |
| Cluster-10108.25756 | | -10.646 |  | Golgi vesicle transport |
| Cluster-10108.13095 | | 1.604 |  | inositol metabolic process//glycolytic process//mannose metabolic process//gluconeogenesis//fructose metabolic process//immune response//antigen processing and presentation//pentose-phosphate shunt//carbon utilization |
| Cluster-10108.20544 | | -2.1901 | -1.2028 | -- |
| Cluster-10108.2764 | | -2.4914 |  | defense response//G protein-coupled receptor signaling pathway |
| Cluster-10108.1348 | | -3.6039 | -2.1768 | defense response |
| Cluster-10108.329 | | -3.4905 |  | defense response to fungus//defense response to bacterium |
| Cluster-10108.17766 | | -1.0601 |  | defense response |
| Cluster-10108.9672 | | 1.3066 |  | defense response to bacterium |
| Cluster-10108.16740 | | -1.0431 |  | defense response |
| Cluster-10108.202 | | -10.609 | -5.9676 | defense response |
| Cluster-10108.3879 | | 5.7975 |  | defense response |
| Cluster-10108.11577 | | 1.274 |  | defense response |
| Cluster-10108.17820 | | -1.4685 |  | defense response |
| Cluster-10108.29487 | | 4.3588 |  | defense response |
| Cluster-10108.27828 | | -3.9939 |  | defense response to bacterium//proteolysis |
| Cluster-10108.12518 | | -1.4809 |  | defense response//transmembrane transport |
| Cluster-10108.23263 | | 5.461 |  | defense response to fungus//defense response to bacterium |
| Cluster-10108.14689 | | 1.222 |  | defense response to other organism |
| Cluster-10108.1414 | | -3.0491 |  | defense response to bacterium//defense response to fungus |
| Cluster-10108.17765 | | -1.2687 |  | defense response |
| Cluster-10108.29809 | | -2.6919 |  | defense response |
| Cluster-9981.0 | | 5.6788 |  | cell wall macromolecule catabolic process//chitin catabolic process |
| Cluster-10108.16910 | | 1.1912 |  | response to pheromone//G protein-coupled receptor signaling pathway |
| Cluster-10108.9306 | | 1.1231 |  | protein trimerization//microtubule-based movement//microtubule-based process |
| Cluster-10108.15084 | | 1.643 | 1.2564 | regulation of DNA-templated transcription, termination//cytolysis//cell motility//microtubule-based process//defense response to Gram-negative bacterium//microtubule-based movement//transcriptional attenuation by ribosome |
| Cluster-10108.13811 | | 1.3978 |  | potassium ion transport//cytoskeleton organization |
| Cluster-10108.7457 | | 1.6845 | 1.0349 | -- |
| Cluster-10108.4797 | | 1.9188 |  | regulation of translation |
| Cluster-10108.13381 | | -1.3118 |  | sensory organ development//cell wall biogenesis//plant-type cell wall organization |
| Cluster-10108.25090 | | 5.4667 |  | chorion-containing eggshell formation |
| Cluster-10108.17115 | | 1.1901 | 1.0557 | chorion-containing eggshell formation//nodule morphogenesis//smoothened signaling pathway//kinetochore assembly//intracellular transport of virus//chromosome segregation |
| Cluster-10108.24484 | | 5.7594 | 8.3699 | chorion-containing eggshell formation |
| Cluster-10108.15608 | | -1.207 |  | mycotoxin biosynthetic process//chorion-containing eggshell formation |
| Cluster-10108.17800 | | -1.5284 |  | chorion-containing eggshell formation |
| Cluster-10108.8287 | | 1.8961 |  | cell wall biogenesis//photosynthesis//plant-type cell wall organization |
| Cluster-10108.22840 | | 2.4226 | 1.6078 | plant-type cell wall organization//photosynthesis//cell wall biogenesis//rRNA processing |
| Cluster-10108.19175 | | 1.0056 |  | cell wall biogenesis//rRNA processing//intracellular protein transport//plant-type cell wall organization//vesicle-mediated transport |
| Cluster-10108.8120 | | -1.1193 |  | chitin catabolic process//cell wall macromolecule catabolic process |
| Cluster-10108.1893 | | -5.7211 |  | -- |
| Cluster-10108.3795 | | 5.6356 |  | -- |
| Cluster-10108.20906 | | 2.1693 |  | -- |
| Cluster-10108.20146 | | 1.7225 |  | -- |
| Cluster-10108.3102 | | -1.504 |  | protein phosphorylation |
| Cluster-10108.22646 | | 2.053 |  | -- |
| Cluster-10108.10010 | | | -1.0366 | fat cell differentiation |
| Cluster-10108.18552 | | 1.1226 | 1.3305 | protein phosphorylation//phosphorylation//serine family amino acid metabolic process |
| Cluster-10108.20920 | | 1.266 |  | copper ion transport//copper ion transmembrane transport |
| Cluster-10108.10848 | | 1.3666 | 1.0267 | telomere maintenance//DNA repair//microtubule-based process//proton transmembrane transport//microtubule-based movement//proteasome assembly |
| Cluster-10108.18079 | | 1.3815 |  | regulation of mitotic metaphase/anaphase transition//microtubule-based movement//microtubule-based process |
| Cluster-10108.3081 | | -3.297 |  | hemolymph coagulation//defense response to bacterium |
| Cluster-10108.7089 | | 3.4642 |  | -- |
| Cluster-10108.552 | | -5.8913 |  | -- |
| Cluster-10108.1324 | | -5.7691 |  | -- |
| Cluster-852.0 | | -6.4869 |  | -- |
| Cluster-10108.6169 | | 1.2819 |  | -- |
| Cluster-10108.2584 | | -1.7426 |  | -- |
| Cluster-10108.19080 | | 1.0499 |  | -- |
| Cluster-10108.29663 | | -4.9798 |  | -- |
| Cluster-8057.0 | | -4.3471 |  | -- |
| Cluster-10108.29744 | | -3.8208 |  | alpha-tubulin acetylation//obsolete acyl-carrier-protein biosynthetic process |
| Cluster-10108.12128 | | -1.8159 |  | -- |
| Cluster-5715.0 | | -5.212 |  | -- |
| Cluster-10108.16493 | | 1.3464 |  | barbed-end actin filament capping |
| Cluster-10108.9423 | | -1.4824 |  | barbed-end actin filament capping |
| Cluster-10108.13634 | | 1.1538 |  | -- |
| Cluster-10108.9026 | | -1.6715 | -1.2038 | -- |
| Cluster-10108.38 | | -4.1128 |  | cilium assembly//negative regulation of centrosome duplication//sucrose metabolic process//starch metabolic process//cell wall modification |
| Cluster-10108.7272 | | -1.4826 |  | -- |
| Cluster-10108.16407 | | -1.2408 |  | Actinobacterium-type cell wall biogenesis |
| Cluster-10108.10392 | | -2.0923 |  | polysaccharide metabolic process |
| Cluster-10108.4975 | | -1.6131 |  | protein phosphorylation |
| Cluster-10108.8852 | | 1.0666 |  | -- |
